# Unveiling Glycerolipid Fragmentation by Cryogenic Infrared Spectroscopy

**DOI:** 10.1021/jacs.1c06944

**Published:** 2021-09-02

**Authors:** Carla Kirschbaum, Kim Greis, Lukasz Polewski, Sandy Gewinner, Wieland Schöllkopf, Gerard Meijer, Gert von Helden, Kevin Pagel

**Affiliations:** † Institut für Chemie und Biochemie, Freie Universität Berlin, 14195 Berlin, Germany; ‡ Fritz-Haber-Institut der Max-Planck-Gesellschaft, 14195 Berlin, Germany

## Abstract

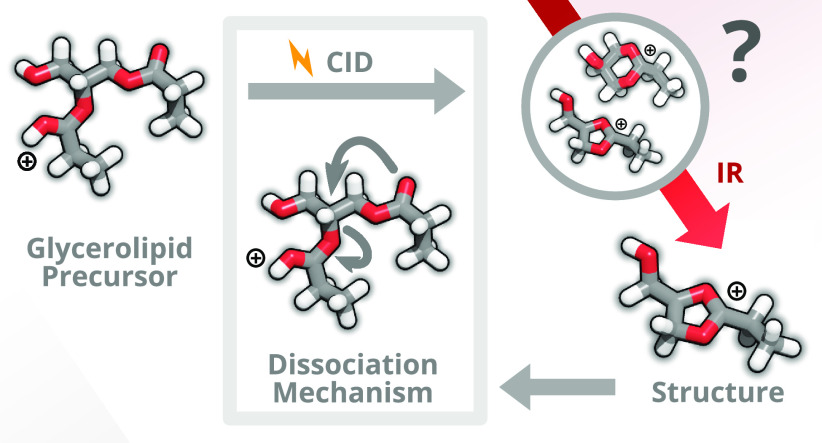

Mass spectrometry is routinely employed for structure elucidation of molecules. Structural information can be retrieved from intact molecular ions by fragmentation; however, the interpretation of fragment spectra is often hampered by poor understanding of the underlying dissociation mechanisms. For example, neutral headgroup loss from protonated glycerolipids has been postulated to proceed via an intramolecular ring closure but the mechanism and resulting ring size have never been experimentally confirmed. Here we use cryogenic gas-phase infrared (IR) spectroscopy in combination with computational chemistry to unravel the structures of fragment ions and thereby shed light on elusive dissociation mechanisms. Using the example of glycerolipid fragmentation, we study the formation of protonated five-membered dioxolane and six-membered dioxane rings and show that dioxolane rings are predominant throughout different glycerolipid classes and fragmentation channels. For comparison, pure dioxolane and dioxane ions were generated from tailor-made dehydroxyl derivatives inspired by natural 1,2- and 1,3-diacylglycerols and subsequently interrogated using IR spectroscopy. Furthermore, the cyclic structure of an intermediate fragment occurring in the phosphatidylcholine fragmentation pathway was spectroscopically confirmed. Overall, the results contribute substantially to the understanding of glycerolipid fragmentation and showcase the value of vibrational ion spectroscopy to mechanistically elucidate crucial fragmentation pathways in lipidomics.

## Introduction

Mass spectrometry (MS) is one of the most-widely used analytical techniques. It provides a high informational content from small amounts of sample, which makes it particularly useful for biomolecular analysis. Of high importance for structural analysis in all omics disciplines are tandem MS techniques, in which molecular ions dissociate into smaller fragments that can yield valuable information about the original molecular structure. However, structure determination is frequently thwarted by unexpected rearrangement reactions including the migration of atom groups^1,2^ or intramolecular cyclization.^3^ In the field of proteomics, gas-phase infrared (IR) spectroscopy has been successfully applied in the past to determine peptide fragment structures and thereby establish pivotal dissociation mechanisms.^4−6^ In spite of significant progress in the understanding of peptide fragmentation, no spectroscopic studies on lipid fragmentation exist to date. The knowledge on crucial dissociation mechanisms in lipidomics is therefore much less substantiated.

**Figure 1:**
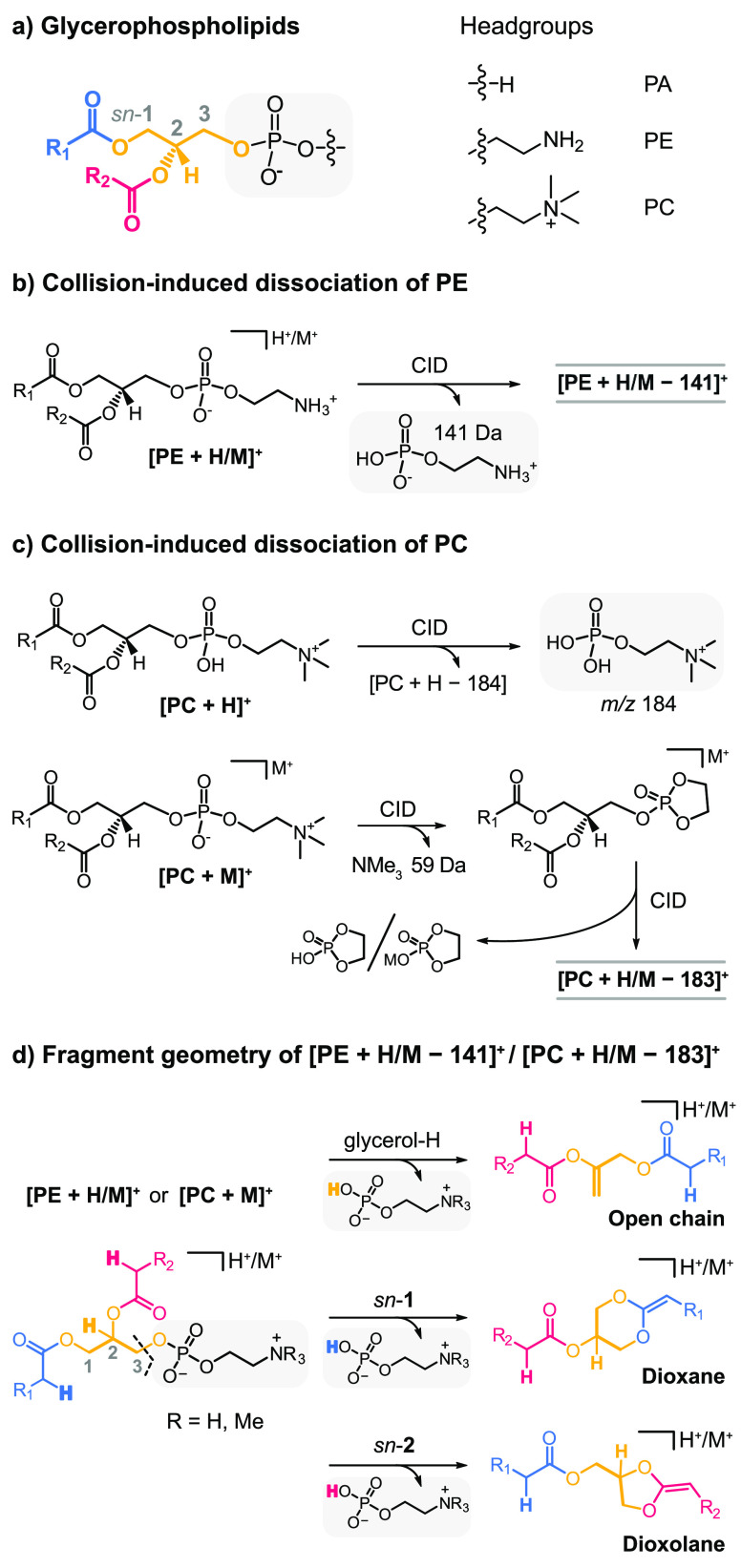
Nomenclature of glycerophospholipids and CID in positive ion mode. a) Glycerophospholipids feature a universal glycerol backbone (yellow) esterified with fatty acids (red and blue) and are classified according to their headgroup (gray). (b) CID of singly charged PE cations induces neutral loss of phosphoethanolamine. c) Protonated PC generates phosphocholine fragments, whereas alkali metal adducts follow a different fragmentation mechanism. d) Proposed fragment structures resulting from neutral headgroup loss from PE or PC. Participation of the glycerol hydrogens leads to a beta-elimination. Participation of the fatty acyl alpha hydrogens at *sn*-1 or *sn*-2 induces cyclization yielding six-membered dioxane or five-membered dioxolane rings, respectively. In the case of PC, the generation of these fragments requires alkali metal adduction of the precursor.

Glycerolipids are ubiquitous in every mammalian cell and fulfill various functions including energy storage, maintenance of cell membranes, and signaling.^7^ As a common structural feature, glycerolipids share a glycerol backbone consisting of three carbon atoms that are referred to using stereospecific numbering (*sn*) from *sn*-1 to *sn*-3 (Figure 1a). Glycerophospholipids are the most abundant lipids in mammals and carry a phosphate-containing headgroup at the *sn*-3 position, whereas the other two positions are occupied by fatty acids.^8^ According to the nature of the headgroup, phospholipids are divided into different classes. The structurally simplest representative is phosphatidic acid (PA), which serves as a precursor for all other glycerophospholipids including phosphatidylcholine (PC) and phosphatidylethanolamine (PE), which are both highly abundant in cell membranes.^9^ Dephosphorylation of PA leads to another important class of glycerolipids called diacylglycerols (DAG). DAGs are important signaling molecules and occur as *sn*-1,2- and *sn*-1,3-isomers.^10^

The most established fragmentation method in lipidomics is collision-induced dissociation (CID). In general, CID spectra of glycerophospholipids are straightforward to interpret because only few abundant product ions result from loss of the headgroup or cleavage at ester bonds.^11^ CID in positive ion mode usually leads to headgroup loss and enables straightforward identification of the lipid class. In negative ion mode, fatty acids are cleaved off as carboxylate anions, which reveal the fatty acyl chain composition, i.e., the number of carbon atoms and degree of unsaturation.^12^ Even though PE and PC are structurally very similar, their fragmentation behavior differs because PC carries a permanent positive charge on the quaternary amine, contrary to neutral PE. Both protonated and metal-adducted PE yield abundant fragments resulting from neutral loss of phosphoethanolamine (141 Da) in positive ion mode (Figure 1b). Upon fragmentation of protonated PC, however, the charge remains on the headgroup and results in a single fragment at *m*/*z* 184 corresponding to phosphocholine (Figure 1c). In contrast, CID of alkali metal adducts yields [PC + M – 59]^+^ ions resulting from the neutral loss of trimethylamine and [PC + H/M – 183]^+^ ions at higher collision energies, which exhibit identical *m*/*z* as [PE + H/M – 141]^+^ ions.^12^

The dissociation mechanism and exact structure of these fragments have been a matter of debate for decades. Before an alternative fragmentation pathway was proposed, headgroup loss from glycerophospholipids was thought to occur via a simple beta-elimination involving a glycerol hydrogen at the *sn*-2 carbon, which is eliminated together with the headgroup.^13,14^ The resulting fragment features a C=C bond between the *sn*-2 and *sn*-3 carbons of the glycerol backbone (Figure 1d). In 2003, Hsu and Turk proposed an alternative fragmentation mechanism for PC, supported by deuteration experiments.^15^ They showed that hydrogens on the glycerol backbone do not participate in the fragmentation process and suggested that an alpha hydrogen of one fatty acyl is transferred to the leaving headgroup instead, accompanied by cyclization. The resulting ring is either a six-membered 1,3-dioxane ring if the fatty acyl at the *sn*-1 position participates or a five-membered 1,3-dioxolane ring in the case of *sn*-2 acyl participation. The experiments suggested a ratio of 2:3 (dioxane/dioxolane) resulting from more labile alpha hydrogens at the *sn*-2 position. Different stabilities of fatty acyls depending on their *sn*-position have been observed in other studies and are being exploited for the distinction of *sn*-isomers in glycerophospholipids.^16,17^ The crucial role of alpha hydrogens in glycerolipid fragmentation was later confirmed for triacylglycerols lacking hydrogens at the α position.^18^

Despite their merits in elucidating glycerophospholipid fragmentation, deuteration experiments only yield indirect evidence of fragment structures by monitoring the deuteration ratio of phosphocholine. Recently, complementary experimental evidence was provided for the structures of sodiated and lithiated glycerophospholipid fragments resulting from neutral headgroup loss using ozone-induced dissociation (OzID),^19^ Paternò-Büchi (PB) reaction,^20^ ultraviolet photodissociation (UVPD),^21^ and CID.^22^ All four approaches unanimously suggested the exclusive formation of dioxolane-type fragments for various glycerophospholipid classes by monitoring MS^3^ fragments. The structure of alkali metal-adducted glycerolipid fragments is therefore well-established, and the knowledge is being employed in routine lipidomics analyses to distinguish *sn*-isomers. The structure of protonated fragments, however, cannot be studied by any of these techniques, and CID of protonated phospholipid fragments is known to yield few informative fragments for structure elucidation.^15,17^ Investigating protonated fragments therefore requires an orthogonal analytical technique and thus constitutes a suitable example to showcase the value of IR ion spectroscopy in studying lipid fragmentation mechanisms.

In this work, we present the first mechanistic study of glycerolipid fragmentation using cryogenic IR ion spectroscopy. Protonated glycerolipid fragments resulting from neutral loss of the headgroup in PE and PC as well as complementary DAG fragments are investigated. Density functional theory (DFT) calculations substantiate the experimental findings and confirm fragment structures. The results provide direct and conclusive evidence that the protonated fragments of glycerolipids are dioxolane rings, in which the positive charge is stabilized between the two ring oxygens.

## Results and Discussion

### Protonated PE, PC, and DAG form Dioxolane-Type Fragments upon Neutral Headgroup Loss

**Figure 2:**
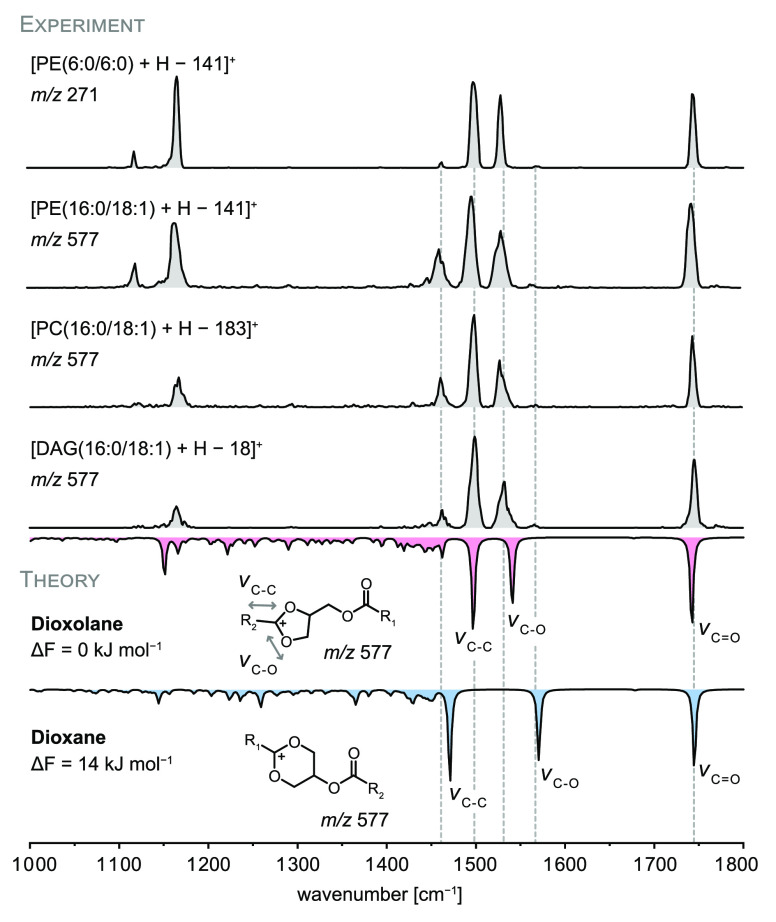
IR spectra of PE, PC, and DAG fragments resulting from neutral headgroup loss. The spectral signature is mainly determined by the core ring structure rather than the fatty acyl length and identical for PE, PC, and DAG (16:0/18:1) fragments. DFT calculations (PBE0+D3/6-311+G(d,p)) of dioxolane and dioxane structures show that the dioxolane structure is energetically favored and matches with the experimental spectra. Computed spectra are shown as inverted traces below the experimental spectra and relative free energies (Δ*F*) at 90 K are given in kJ mol^–1^.

The fragmentation of protonated PE was studied using the model lipid PE(6:0/6:0) bearing two hexanoic acid residues. The protonated species was dissociated by in-source fragmentation, which is based on collisions between the molecular ions and residual gas in the source region and thus equivalent to CID (Figure S1). As the neutral loss of phosphoethanolamine is the dominant fragmentation channel for PE, [PE + H – 141]^+^ ions are formed in high abundance (Figure S2). In the setup used for cryogenic IR ion spectroscopy described previously,^23,24^*m*/*z*-selected fragment ions thermalized at 90 K are encapsulated in superfluid helium droplets and their release induced by multiple photon absorption events is monitored as a function of the tunable photon energy to yield a high-resolution IR spectrum. The IR spectrum of [PE(6:0/6:0) + H – 141]^+^ is depicted in Figure 2. Throughout this work, experimental IR spectra are represented in gray, whereas computed spectra are colored (red = dioxolane, blue = dioxane). The IR spectrum of [PE(6:0/6:0) + H – 141]^+^ displays two diagnostic bands between 1500 and 1550 cm^–1^ and a well-defined band around 1750 cm^–1^ derived from the carbonyl stretching vibration (*v*_C=O_). The presence of only one carbonyl band instead of two provides a first indication that the fragment structure features one free carbonyl group, whereas the second has engaged in cyclization. In order to interpret the IR spectrum, DFT calculations at the PBE0+D3/6-311+G(d,p) level of theory were carried out for all three candidate structures depicted in Figure 1d: open chain, dioxane, and dioxolane structures. First, the preferred site of protonation was determined for each motif. Preliminary calculations on model structures unambiguously confirmed that protonation of the C=C bond is energetically most favorable for all structure motifs (Figure S3 and S4). The positive charge is thus located at the sp^2^ carbon between the two ring oxygens, where it is stabilized by mesomeric dislocation. This fixed charge location and concomitant absence of a C=C bond next to the ring explains the different dissociation behaviors of protonated glycerolipids versus metal adducts. The carbonyl group of the second fatty acid furthermore interacts with the charged site in the most stable structures. The dioxolane model structure featuring a noncovalent carbonyl oxygen–ring carbon interaction of 2.6 Å yields a very good match with the experiment. In the computed IR spectra of dioxane structures, the frequency of the carbonyl stretching vibration is in good agreement with the experiment but the main vibration bands arising from the dioxane ring are significantly shifted. The computed dioxane structures are furthermore destabilized by ≥10 kJ mol^–1^ (free energy at 90 K) compared to the most stable dioxolanes. The open chain structure was discarded due to the unsatisfactory spectral match. The computed IR spectra of [PE(6:0/6:0) + H – 141]^+^ are depicted in the Supporting Information (Figure S5). Combined with the computed data, the experimental spectrum provides clear evidence that dioxolane structures are almost exclusively formed.

In order to study the impact of the lipid chain length and degree of unsaturation on the spectral signature, the IR spectrum of [PE(16:0/18:1) + H – 141]^+^ was recorded (Figure 2). The position of the main absorption bands is not affected by the extended lipid chains compared with the fragment generated from PE(6:0/6:0). Differences are only discernible on the level of relative band intensities. In particular, the intensity of the band at 1450 cm^–1^ increases with increasing chain length because it arises mainly from CH_2_ bending vibrations of the aliphatic chains. Overall, the fatty acids have no major impact on the IR spectrum. The positions of the main absorption bands remain largely unaffected by variation of the lipid chains, demonstrating that the dioxolane core structure determines the IR signature. As shown in the computed spectra in Figure 2, the two absorption bands at 1500 and 1540 cm^–1^ are originating from diagnostic C–C and C–O stretching vibrations of the dioxolane ring and dominate the spectrum. The weak band around 1570 cm^–1^ in all experimental spectra, which coincides with one of the two main bands in the computed dioxane spectra, points toward the presence of minor dioxane-type fragments. Computed spectra of dioxolane and dioxane structures suggest that the main band in the dioxolane spectra (1500 cm^–1^) is of equal intensity as the band at 1570 cm^–1^ in the dioxane spectra. The relative abundance of dioxane was thus determined to be 2–3% by dividing the area under the peak at 1570 cm^–1^ by the area under the main peak at 1500 cm^–1^. CID of PE(d31–16:0/18:1) yielded approximately the same ratio of dioxolanes as a control experiment (Figure S9).

In the next step, the structures of fragments generated from PE were compared with the corresponding fragments obtained from PC and DAG. [PE + H – 141]^+^, [PC + H – 183]^+^, and [DAG + H – 18]^+^ ions, resulting from neutral loss of the headgroup from PE and PC or loss of water from DAG, exhibit identical *m*/*z* values if the fatty acid composition is identical for all precursors. As shown in Figure 2, the overall spectral signature of the fragment at *m*/*z* 577 is independent of the precursor, which suggests identical fragment structures (Figures S6 and S7). Slight differences concerning relative band intensities and bandwidths arise from day-to-day fluctuations of the laser power and bandwidth as well as varying abundance of the precursor ions depending on the lipid class. The [PC + H – 183]^+^ ions, for example, yield a very low ion signal because metal-adducted fragments dominate the mass spectrum (Figure S2). Especially the peaks at 1150 and 1450 cm^–1^ are susceptible to the laser power.

In conclusion, the formation of protonated dioxolane-type structures can be regarded as a universal phenomenon in glycerolipid fragmentation. However, the results do not agree with the deuteration experiments conducted on protonated PC, which suggested a considerable fraction of dioxanes (40%).^15^ Several explanations are possible. First, a different fragmentation mechanism has been studied in that work. Instead of probing protonated fragments generated from sodiated PC, protonated precursor ions with deuterated hydrocarbon chains were fragmented (cf. Figure 1c). The fraction of dioxane structures was thus indirectly determined by monitoring the H/D ratio in phosphocholine fragments, whereas the lipid portion was lost as neutral species. It is imaginable that the fragmentation of protonated PC occurs via an increased participation of *sn*-1 alpha hydrogens. The exact structure of the fragment, however, cannot be probed directly by MS-based methods due to the absence of a charge. A second explanation might be that initially both dioxolanes and dioxanes are formed, which then interconvert in the gas phase and finally converge toward the more stable dioxolane structure. This hypothesis was tested by computing transition states between dioxane and dioxolane model structures. As shown in Figure S8, the activation energy required for a dioxane–dioxolane conversion is well above 150 kJ mol^–1^ for model structures. Even though energy can in principle be taken up by the fragments via ion–molecule collisions in the source region following dissociation of the precursor ions, it is rather unlikely that such high conversion barriers can be overcome. We therefore assume that dioxolanes are exclusively formed in the initial fragmentation process.

### Alkali Metal-Adducted PC Fragments via a Cyclic Phosphate Intermediate

**Figure 3:**
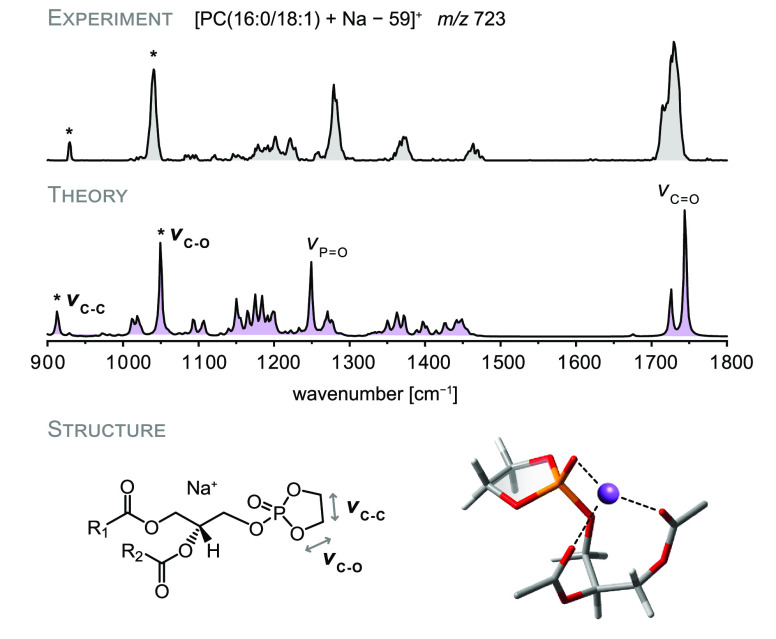
IR spectrum of the [PC(16:0/18:1) + Na – 59]^+^ ion generated by neutral loss of trimethylamine from sodiated PC. The experimental spectrum features two diagnostic vibrations predicted by DFT calculations (PBE0+D3/6-311+G(d,p)) for a cyclic phosphate structure. The depicted 3D structure is truncated to two carbon atoms per fatty acid to enhance visibility.

Alkali metal-adducted PC readily loses trimethylamine, resulting in the [PC + M – 59]^+^ fragment, which is postulated to exhibit a cyclic phosphate (cf. Figure 1c).^15^ To confirm the structure of this intermediate fragment, which can further dissociate to yield the dioxolane fragment discussed in the previous section, [PC(16:0/18:1) + Na]^+^ ions (*m*/*z* 782) were generated by the addition of sodium acetate. The sodiated precursor ion undergoes neutral loss of trimethylamine upon appropriate source conditions (Figure S2). The IR spectrum of the [PC(16:0/18:1) + Na – 59]^+^ ion at *m*/*z* 723 is depicted in Figure 3 together with a computed spectrum of the suggested cyclic phosphate structure. Overall, the spectral signature matches well with the computed IR spectrum. The symmetric and antisymmetric stretching vibrations of the two carbonyl groups are at the correct position and partly resolved. Another characteristic vibration is the P=O stretching vibration between 1200–1300 cm^–1^, which is slightly shifted relative to the computed frequency. The most important vibrations providing evidence for the cyclic phosphate are located at lower wavenumbers: the C–C stretching vibration of the two ring carbons yields a characteristic, narrow band between 900–950 cm^–1^. The C–O stretching vibrations of the ring are located around 1050 cm^–1^ and of high relative intensity. The presence of these two characteristic bands as well as the overall good match between theory and experiment provide first direct experimental evidence that the sodiated intermediate fragment occurring in the phosphatidylcholine fragmentation pathway indeed features a five-membered phosphate ring. 3D structures of the full-length computed conformers are depicted in Figure S10.

### Protonated DAG Forms Dioxolane- and Dioxane-Type Fragments Depending on the Initial Geometry

**Figure 4:**
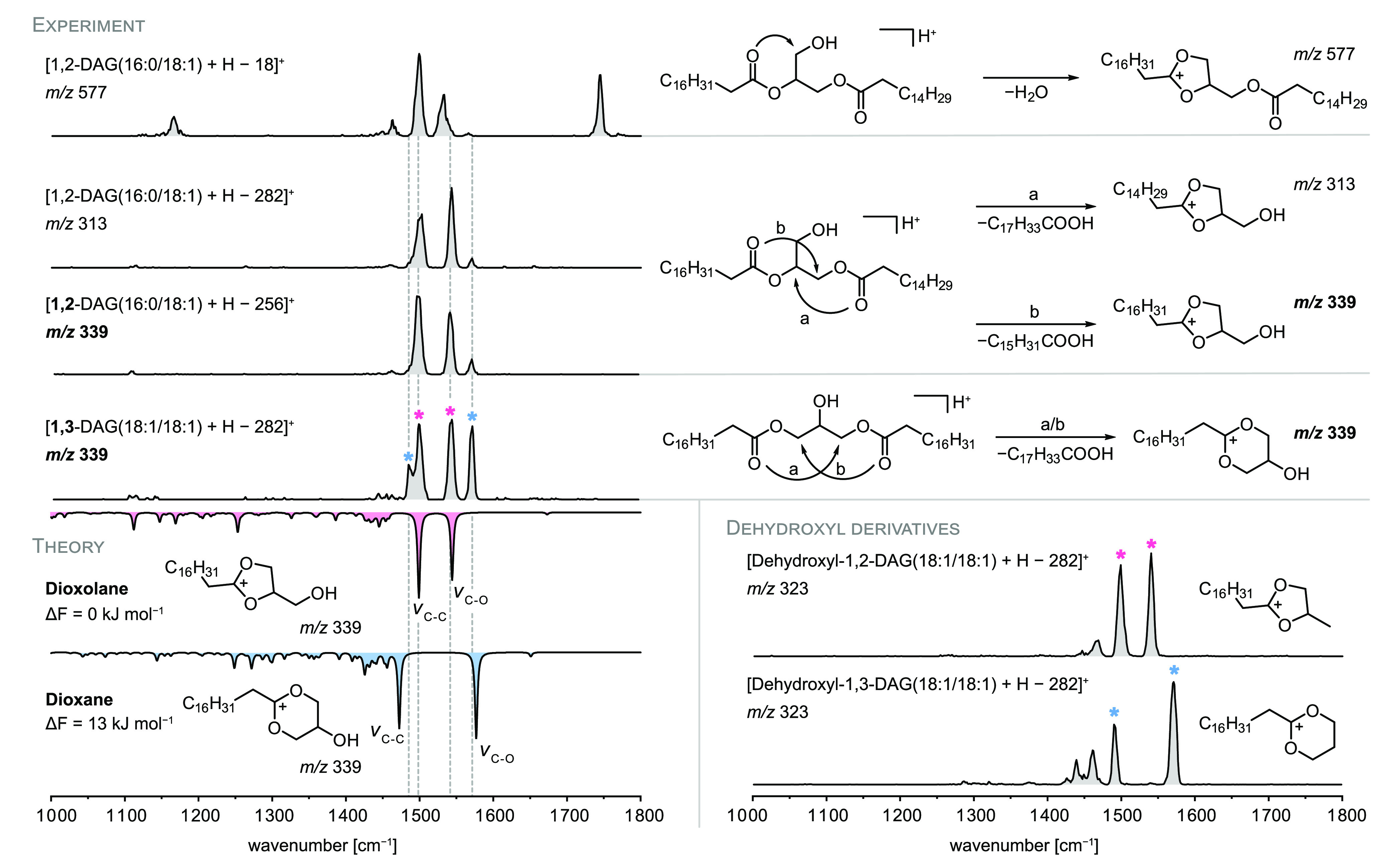
IR spectra and structures of fragments generated from 1,2- and 1,3-DAGs. DAGs readily lose one fatty acid to form fragment structures resembling the previously observed dioxolane ring resulting from water loss. In theory, 1,2-DAGs can only form dioxolane rings, whereas 1,3-DAGs must yield dioxane rings upon neutral fatty acid loss. Both ring sizes are observed in both spectra due to acyl chain migration, as confirmed by DFT calculations at the PBE0+D3/6-311+G(d,p) level of theory. Prevention of acyl chain migration in tailor-made dehydroxyl derivatives leads to pure dioxolane and dioxane spectra.

The enzymatic removal of the phosphate headgroup from glycerophospholipids results in 1,2-DAGs bearing a hydroxyl group at the *sn*-3 position (Figure 4). However, a smaller pool of DAGs in the human body is constituted by 1,3-DAGs, which are distinguished by processing enzymes and by shape-sensitive analytical techniques such as ion mobility spectrometry.^10,25^ 1,2- and 1,3-DAGs can interconvert by acyl chain migration, a process catalyzed by acidic and basic conditions.^26^ Upon CID, protonated DAGs readily lose water, as shown previously. Another important fragmentation pathway involves the neutral loss of a fatty acid. The fragments formed via this second dissociation channel provide a wealth of additional information on the formation of dioxolane and dioxane structures. 1,2-DAG(16:0/18:1) can lose either palmitic acid or oleic acid, resulting in two different fragment ions at *m*/*z* 339 and *m*/*z* 313, respectively (Figure S2). Both fragments were studied by IR spectroscopy and yielded very similar spectra (Figure 4), which indicates identical core structures (Figures S11 and S12). The fragment at *m*/*z* 339 yielded the same spectral signature whether generated from 1,2-DAG(16:0/18:1) or 1,2-DAG(18:1/18:1) (Figure S12). The absence of absorption bands above 1600 cm^–1^ witnesses the absence of a carbonyl group, indicating that the remaining fatty acid has engaged in an intramolecular ring-closure reaction in the gas phase. The spectra display two main bands at 1500 and 1550 cm^–1^, which closely resemble the spectrum of [DAG(16:0/18:1) + H – 18]^+^ resulting from water loss. From these observations, it is reasonable to assume the formation of a dioxolane ring upon neutral loss of a fatty acid. As shown in Figure 4, the geometry of 1,2-DAGs indeed imposes the formation of dioxolane rings because the fatty acyls are located on neighboring positions on the glycerol backbone. The formation of dioxane rings, on the other hand, is precluded by the geometry of the precursor ion. The opposite is true for 1,3-DAGs, in which the two fatty acid substituents are separated by the *sn*-2 carbon on the glycerol backbone. From a geometrical point of view, the formation of dioxolanes is precluded in 1,3-DAGs, which raises the intriguing possibility to observe the less stable dioxane rings upon fragmentation. As shown in Figure 4, the IR spectra of the fragment ion at *m*/*z* 339 are indeed different for 1,2- and 1,3-DAG precursor ions. However, this difference only concerns the relative band intensities rather than band positions. As confirmed by DFT calculations, both dioxolane- and dioxane-type fragments are present in both spectra with the ratio of dioxane structures being significantly elevated in the spectrum of 1,3-DAG. The most likely explanation for the observation of both structures in both spectra despite the contradicting geometrical assumptions is acyl chain migration. The transfer of an acyl moiety to a different position on the glycerol backbone is initiated by the hydroxyl group attacking the electrophilic carbonyl carbon.^27^ This process can in principle occur in solution or in the source region of the instrument. As acyl chain migration of DAGs in organic solvents is, however, minimal at room temperature,^26^ the rearrangement is suspected to occur in the source region of the instrument, probably aided by methanol as protic solvent.^28^ Also the observed ratios of dioxolanes and dioxanes in the IR spectra do not correspond to the typical 1:2 equilibrium of 1,2:1,3-DAGs^26^ in solution.

In order to suppress acyl chain migration, the nucleophilicity of the free hydroxyl group in DAGs must be reduced. This can be achieved, for instance, by methylation. However, acyl chain migration can already occur before or during the methylation reaction, and therefore we chose to synthesize dehydroxyl derivatives of 1,2- and 1,3-DAG(18:1/18:1) to render acyl chain migration completely impossible. 1,2- and 1,3-Propylene glycol dioleate were synthesized by esterification of oleic acid with 1,2-propanediol and 1,3-propanediol, respectively, following published protocols (Scheme S1).^29,30^ The dehydroxyl derivatives were found to undergo neutral loss of one fatty acid analogous to DAGs. The fragments at *m*/*z* 323 correspond to the fragments at *m*/*z* 339 obtained from DAGs lacking the hydroxyl group. In perfect agreement with the expectations, the protonated fragments obtained from the dehydroxyl derivatives 1,2- and 1,3-propylene glycol dioleate yield clean, distinguishable IR spectra (Figure 4) matching the computed IR spectra of dioxolane and dioxane rings, respectively (Figure S13). Fragmentation of 1,3-propylene glycol dioleate thus allowed for the first time the observation of dioxane-type fragments, which are energetically less favored than dioxolane structures. The result demonstrates that the preference of glycerolipids to generate dioxolane-type fragments can be circumvented by rational design of the precursor ion structure.

## Conclusions

Cryogenic IR ion spectroscopy in combination with computational chemistry expands our understanding of tandem MS fragmentation mechanisms by investigating fragment structures. Contrary to other MS-based methods, IR spectroscopy does not depend on the presence of a specific structure motif, which renders the technique highly versatile and applicable to various molecular classes in both ion polarities. In this work, IR spectroscopy provided the first direct experimental evidence of protonated dioxolane-type fragments generated throughout different glycerolipid classes and fragmentation routes. Even though not all phospholipid classes were included, the consistency of their fragmentation behavior^12^ suggests that the results are generally valid for glycero(phospho)lipids. The results are consistent with recent studies on metal adducts and corroborate a previously proposed dissociation mechanism involving the participation of *sn*-2 alpha hydrogens while excluding other pathways resulting in open chain or dioxane fragments. As a second major finding, the formation of dioxolane- and dioxane-type fragments can be predicted and tuned via the original molecular geometry in diacylglycerol regioisomers and rationally designed dehydroxyl derivatives thereof. With these results, cryogenic IR spectroscopy breaks new ground in lipidomics, by enabling the characterization of fundamental tandem MS fragmentation channels to confirm, complement or discard long-standing hypothetical dissociation mechanisms.

## Methods

PE(6:0/6:0), PE(16:0/18:1(9*Z*)), PE(d31-16:0/18:1(9*Z*)), PC(16:0/18:1(9*Z*)), and 1,2-DAG(16:0/18:1(9*Z*)) were purchased from Avanti Polar Lipids (Alabaster, AL). 1,2-DAG(18:1(9*Z*)/18:1(9*Z*)) and 1,3-DAG(18:1(9*Z*)/18:1(9*Z*)) standards, methanol, and sodium acetate were obtained from Sigma-Aldrich (Taufkirchen, Germany). Stock solutions (10–100 mM) were prepared in methanol and diluted to 100 μM for measurements. DAG solutions were freshly prepared before each measurement. All solutions were stored at −25 °C.

The dehydroxyl derivatives 1,2-propylene glycol dioleate and 1,3-propylene glycol dioleate were prepared according to published protocols (Scheme S1).^29,30^ All reagents were purchased from Sigma-Aldrich, and the reaction was carried out under nitrogen atmosphere. 1,2- or 1,3-Propylene glycol (0.25 mmol) was dissolved in dry dichloromethane (DCM, 20 mL). *N*,*N*’-Dicyclohexylcarbodiimide (DCC, 0.6 mmol) and 4-dimethylaminopyridine (DMAP, 0.05 mmol) were added under nitrogen flow at 0 °C. Oleic acid (0.55 mmol) in dry DCM (2 mL) was added in excess, and the mixture was stirred at 0 °C for 5 min and then for 3 h at room temperature. The solution was washed with 0.5 M HCl (20 mL) and saturated sodium hydrogen carbonate solution (20 mL) and dried with sodium sulfate. After filtration, the remaining urea precipitate was removed by centrifugation, the solvent was evaporated under nitrogen flow, and the solid was redissolved in methanol. The products were stored at −25 °C until use.

IR spectra were measured on a custom-built instrument described previously.^23,24^ Analytes are ionized by nanoelectrospray ionization and are subjected to in-source fragmentation as shown in Figure S1. The fragments are mass-to-charge selected in a quadrupole and deflected into a hexapole ion trap filled with helium buffer gas. The trap is additionally cooled by liquid nitrogen (90 K). After thermalization of the ions, the helium buffer gas is pumped out and superfluid helium droplets coaxially traverse the trap to pick up trapped ions. The helium droplets are generated by the expansion of pressurized helium through a pulsed Even-Lavie valve (nozzle temperature = 21 K). Ions inside helium droplets are rapidly cooled to the equilibrium temperature of 0.4 K but can freely vibrate in the cryogenic helium matrix. The doped droplets travel toward the detection region, where the pulsed beam of helium droplets overlaps spatially and temporally with the pulsed beam of IR photons generated by the Fritz Haber Institute free-electron laser (FHI FEL)^31^ with a macropulse repetition rate of 10 Hz. The consecutive absorption of multiple photons by the ion leads to evaporation of the helium shell until the bare ion is released from the droplet and detected by time-of-flight MS. The MS signal is employed as an indirect measure for IR absorption, and IR spectra are generated by monitoring the ion count while scanning the tunable wavenumber of the FHI FEL in steps of 2 cm^–1^. All spectra shown in this work were averaged over two separate measurements. Peak areas in the experimental IR spectra were computed using the integration tool in OriginPro 2020 from 1485 to 1510 cm^–1^ and from 1560 to 1575 cm^–1^. The dioxane/dioxolane ratio was determined for all fragment spectra depicted in Figure 2 by dividing the second integral by the first. All obtained values consistently lie in between 0.02–0.03.

CID tandem MS spectra of deuterated PE were recorded on a Synapt G2-S HDMS ion mobility-mass spectrometer (Waters Corporation) modified by a drift tube instead of the commercial traveling wave ion mobility cell.^32^ Ions were generated by nanoelectrospray ionization, *m*/*z* selected in the quadrupole and fragmented in the trap collision cell.

Fragment structures were computed by sampling the conformational space and DFT optimization of selected structures, followed by harmonic frequency calculations and comparison with the experimental IR spectra. The conformational space was sampled using CREST^33^ with the semiempirical method GFN2-xTB^34^ and default settings. Before the actual sampling of conformers, protonation sites were identified using the protonation tool implemented in CREST, as further detailed in the Supporting Information. The protomers exhibiting the lowest relative free energy were subjected to conformer sampling in CREST, and selected conformers were optimized at the PBE0+D3/6-311+G(d,p)^35,36^ level of theory in Gaussian 16.^37^ Harmonic vibrational spectra were computed at the same level of theory and scaled by a scaling factor of 0.965, in accordance with previous works.^38,39^ Harmonic free energies (Δ*F*) were determined at a temperature of 90 K corresponding to the temperature in the ion trap. Transition states of model structures truncated to three carbon atoms were obtained by scanning the potential energy surface (PES) of the bond to be broken in Gaussian 16. The structure at the saddle point of the PES was optimized as a transition state at the PBE0+D3/6-311+G(d,p) level of theory. The existence of one imaginary frequency was confirmed by a frequency analysis of the optimized transition state. The transition states were linked to reactants and products by an intrinsic reaction coordinate calculation.

## Supporting Information Available

Conditions for in-source fragmentation, computed IR spectra, *xyz* coordinates of optimized fragment structures, and reaction scheme for the synthesis of propylene glycol dioleate (PDF)
