# Supplementary material for: Unveiling Glycerolipid Fragmentation by Cryogenic Infrared Spectroscopy
Source: J Am Chem Soc. 2021 Sep 2;143(36):14827–34. doi: 10.1021/jacs.1c06944 (PMC8447261; doi:10.1021/jacs.1c06944)
Supplement: Supplementary file 1 — ja1c06944_si_001.pdf [file ja1c06944_si_001.pdf]

Unveiling Glycerolipid Fragmentation by Cryogenic Infrared Spectroscopy

Supplementary Information

Carla Kirschbaum,[a],[b] Kim Greis,[a],[b] Lukasz Polewski,[a],[b] Sandy Gewinner,[b] Wieland Schöllkopf,[b] Gerard Meijer,[b] Gert von Helden,[b] and Kevin Pagel*[a],[b]

[a] Institut für Chemie und Biochemie, Freie Universität Berlin, 14195 Berlin, Germany

[b] Fritz-Haber-Institut der Max-Planck-Gesellschaft, 14195 Berlin, Germany

Correspondence to: kevin.pagel@fu-berlin.de

Table of Contents

Abbreviations 3

In-source Fragmentation 3

Figure S1. 3

Figure S2. 4

Neutral Headgroup Loss from Glycerolipids 5

Protomers of Model Structures 5

Figure S3. 6

Figure S4. 7

Computed IR Spectra 8

Figure S5. 8

Figure S6. 9

Figure S7. 10

Conversion Energy Barriers 10

Figure S8. 10

CID of PE(d31-16:0/18:1) 11

Figure S9. 11

Phosphatidylcholine Fragmentation Pathway 12

Figure S10. 12

Neutral Fatty Acid Loss from Diacylglycerols 13

Computed IR Spectra 13

Figure S11. 13

Figure S12. 14

Figure S13. 15

Synthesis of Propylene Glycol Dioleate 16

Scheme S1 16

XYZ Coordinates of Computed Conformers 17

PE(3:0/3:0) + H − 141]$^+$ 17

PE(3:0/3:0) + H − 141]$^+$ Transition States 19

PE(6:0/6:0) + H − 141]$^+$ 21

PE(16:0/18:1) + H − 141]$^+$ 27

PC(16:0/18:1) + Na − 59]$^+$ 38

DAG(16:0/18:1) + H − 282]$^+$ 40

DAG(16:0/18:1) + H − 256]$^+$ 45

Dehydroxyl-DAG(18:1/18:1) + H − 282]$^+$ 50

Abbreviations

PE = Phosphatidylethanolamine; PC = Phosphatidylcholine; DAG = Diacylglycerol

In-source Fragmentation

In-source fragmentation is comparable to collision-induced dissociation (CID) that is based on acceleration of molecular ions and subsequent collisions with a buffer gas. In the case of in-source fragmentation, the role of the buffer gas is adopted by residual gas molecules in the differentially pumped source region. In the present setup, fragmentation is induced by accelerating the ions generated by nano-electrospray ionization (nESI) in the region depicted below, which comprises a source block and two ring electrode ion guides (IG). The source block and offset of the first ion guide are set to the maximum voltage provided by the power supplies (150 V). The ions are thus strongly accelerated starting from a high potential towards the second ion guide and then decelerated by an elevated potential on the endcap of the second ion guide. Both the high offset voltage on the source block and first ion guide, and the higher potential at the end of the second ion guide compared to the IG 2 offset, are important to induce fragmentation. Optimal voltage combinations are manually tuned for each parent ion. Typical values are shown in the figure below.

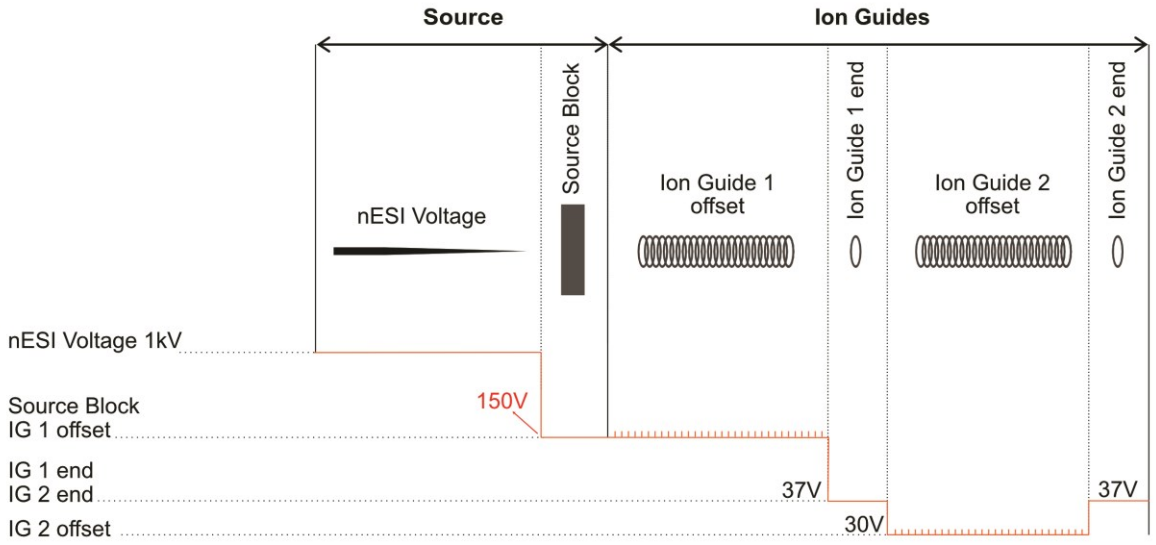

Figure S1. Voltage scheme of the source region where in-source fragmentation occurs. Adjustment of the individual voltages indicated on the scheme is crucial to promote fragmentation of precursor ions generated by nESI. Typical values employed for glycerolipid fragmentation are shown in the scheme. The drawing is not to scale.

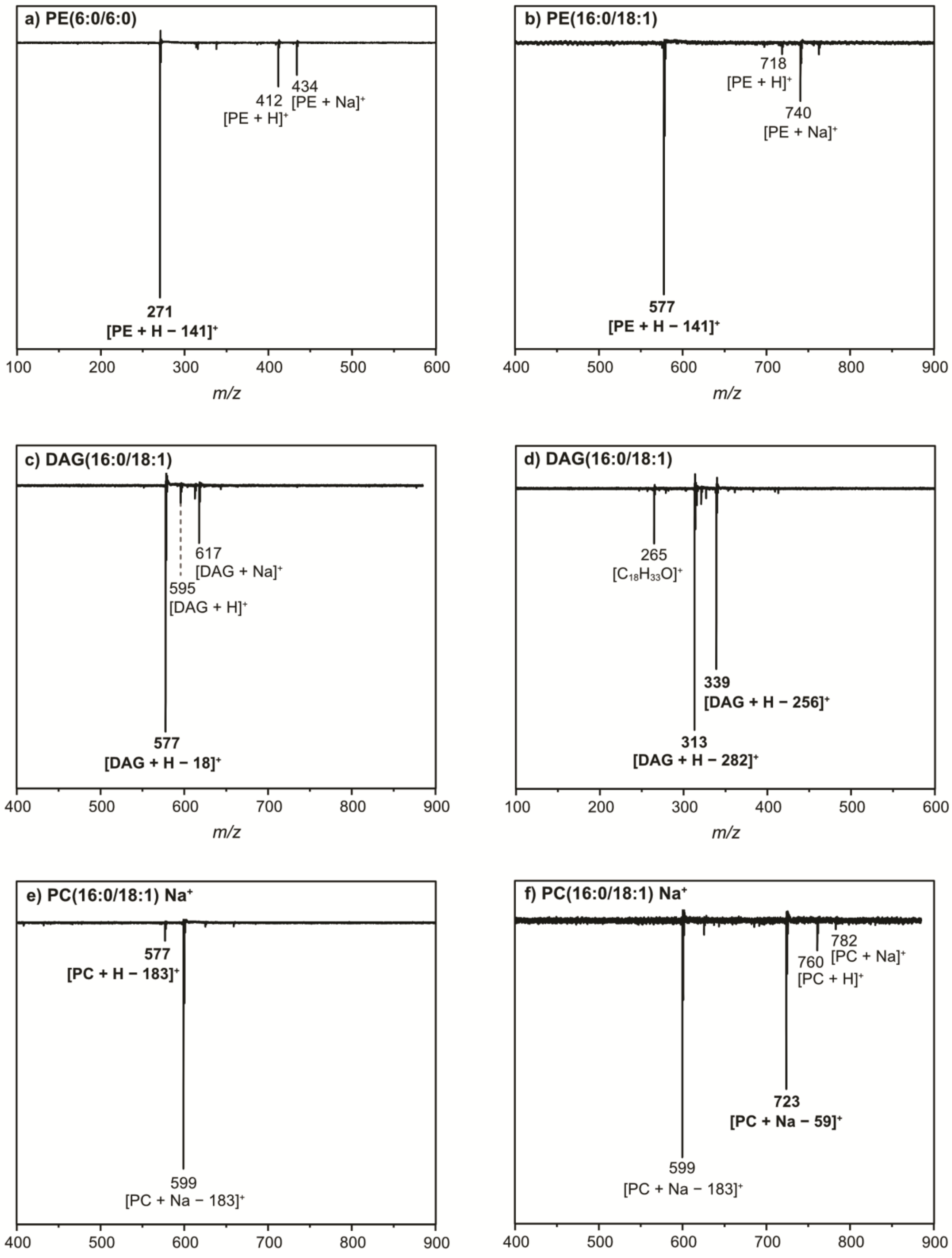

Figure S2. Mass spectra showing in-source fragmentation of glycerolipids. **a)/b)** Protonated PE(6:0/6:0) and PE(16:0/18:1) readily lose phosphoethanolamine (141 Da). **c)** Protonated DAG(16:0/18:1) eliminates water (18 Da). **d)** By applying harsher conditions than in (c), DAG(16:0/18:1) eliminates either of the two fatty acids. Oleic acid is found as acylium cation in the spectrum (*m/z* 265). **e)** Sodiated PC(16:0/18:1) mainly yields sodiated fragments upon neutral loss of the headgroup but also a smaller fraction of protonated fragments. **f)** Using softer conditions than in (e), sodiated PC(16:0/18:1) loses trimethylamine to yield $[PC(16:0/18:1) + Na - 59]^+$ fragments.

Neutral Headgroup Loss from Glycerolipids

Protomers of Model Structures

Protonation sites on glycerolipid fragments were identified using the protonation tool implemented in CREST (keyword *-protonate*) employing the semi-empirical method GFN2-xTB and default settings. The search was performed on model structures featuring three carbon atoms per acyl chain to speed up the calculations. Three different protomers were initially identified for the dioxolane structure. The energetically most favored protonation site is at the C=C bond adjacent to the ring, followed by protonation of the carbonyl oxygen and protonation of the C-O-C oxygen outside of the ring. The latter structure is energetically unfavored and was discarded during conformational sampling. When comparing the relative free energies of DFT-optimized conformers at the PBE0+D3/6-311+G(d,p) level of theory, C=O protonation can be ruled out because the conformers are disfavored by 100 kJ mol^{-1} relative to the lowest-energy conformer featuring C=C protonation. In the dioxolane structures protonated at the C=C bond, the positive charge is very well-stabilized by the two adjacent ring oxygens. The same result was obtained for dioxane structures. The initial search for protonation sites yielded only two protomers with either C=C or C=O protonation, and the latter was disfavored by 100 kJ mol^{-1} after the conformational sampling and DFT optimization. In the most stable dioxolane and dioxane structures protonated at the C=C bond, the carbonyl oxygen interacts with the positive charge in the ring. A lack of this interaction leads to destabilization by ca. 50–60 kJ mol^{-1} and a significant blueshift of the carbonyl stretching vibration, which is not supported by the experiment. The positive charge is thus stabilized by two ring-oxygens and the interacting carbonyl oxygen in both dioxolane and dioxane structures. The distance between the carbonyl oxygen and the ring carbon is between 2.5–2.6 Å.

For the open chain structure, three protonation sites were identified on the two different carbonyl oxygens and the C=C bond. C=C protonation is initially energetically unfavored because the positive charge is only stabilized by one adjacent oxygen. However, after the conformational sampling, C=C protonation yielded cyclic structures after an intramolecular rearrangement. The positive charge is stabilized by two oxygen atoms in the ring and by intramolecular interaction with the carbonyl oxygen at a distance of 1.6 Å. This structure is, among all sampled structures, the most energetically favored. However, the IR signature does not correspond to the experiment, which indicates that the open structure is not initially formed in the dissociation process. In particular, the covalent character of the carbonyl oxygen--sp^2-carbon interaction leads to a bicyclic character of the ion and concomitant absence of a C=O stretching vibration, which is clearly present in the experimental spectra.

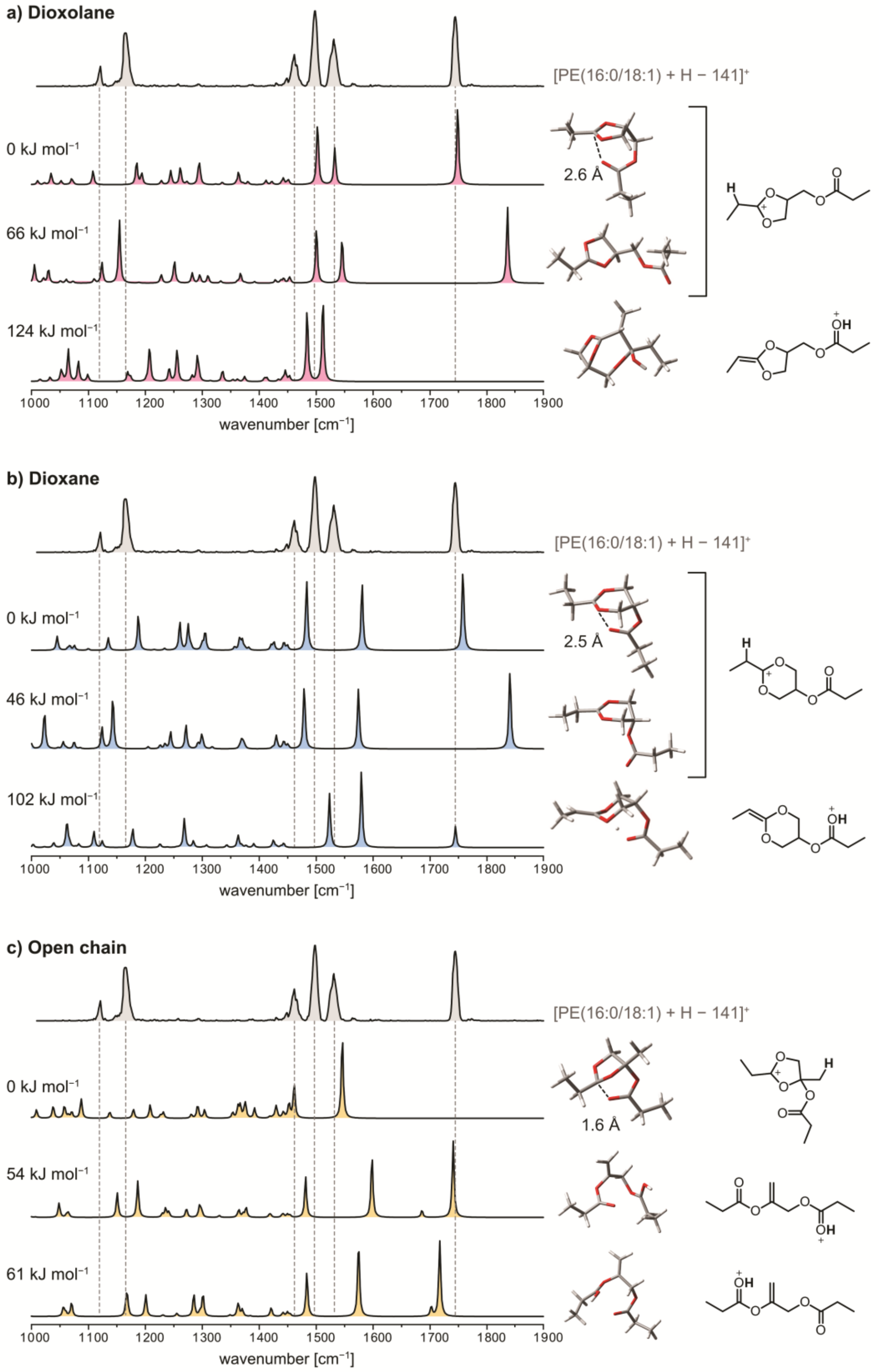

Figure S3. Computed IR spectra of protomers of **a)** dioxolane, **b)** dioxane and **c)** open chain model structures truncated to three carbons per fatty acid (3:0/3:0). Protonation of the C=C bond is in all cases energetically more favorable than protonation of a carbonyl oxygen. The best match with the experimental spectrum of $[PE(16:0/18:1) + H - 141]^+$ (gray) is obtained for dioxolanes protonated at the C=C bond and the carbonyl oxygen interacting with the positive charge (d = 2.6 Å). Spectra were computed at the PBE0+D3/6-311+G(d,p) level of theory, and relative free energies at 90 K refer to the most stable conformer of each structure motif. XYZ coordinates of all computed structures are listed on page 17ff.

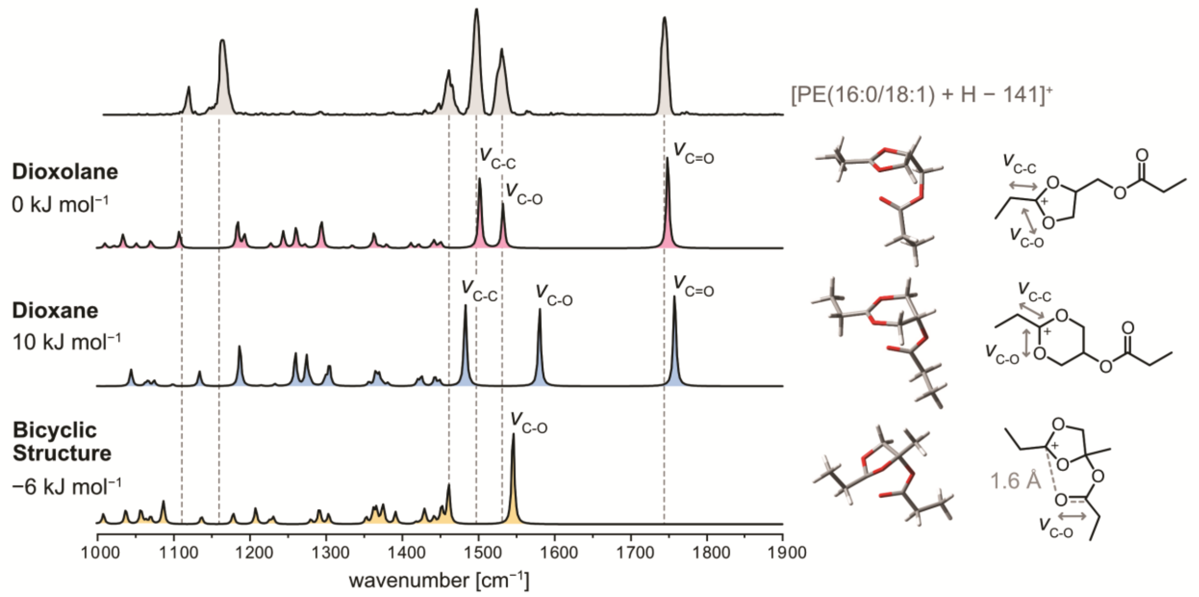

Figure S4. Computed IR spectra, structures and relative free energies at 90 K of the lowest-energy protomers from Figure S3 for each fragment type. All model structures are truncated to three carbons per fatty acid and protonated at the C=C bond. The bicyclic structure emerging from the initial open chain is the most energetically favored structure but the carbonyl stretching vibration at 1750 cm^{-1} in the experimental spectrum of $[PE(16:0/18:1) + H - 141]^+$ (gray) is missing in the computed IR spectrum due to the covalent character of the C=O--C^+ bond (d = 1.6 Å). The protonated dioxolane yields the best match with the experiment and is energetically favored by 10 kJ mol^{-1} compared to the protonated dioxane isomer. Spectra were computed at the PBE0+D3/6-311+G(d,p) level of theory.

Computed IR Spectra

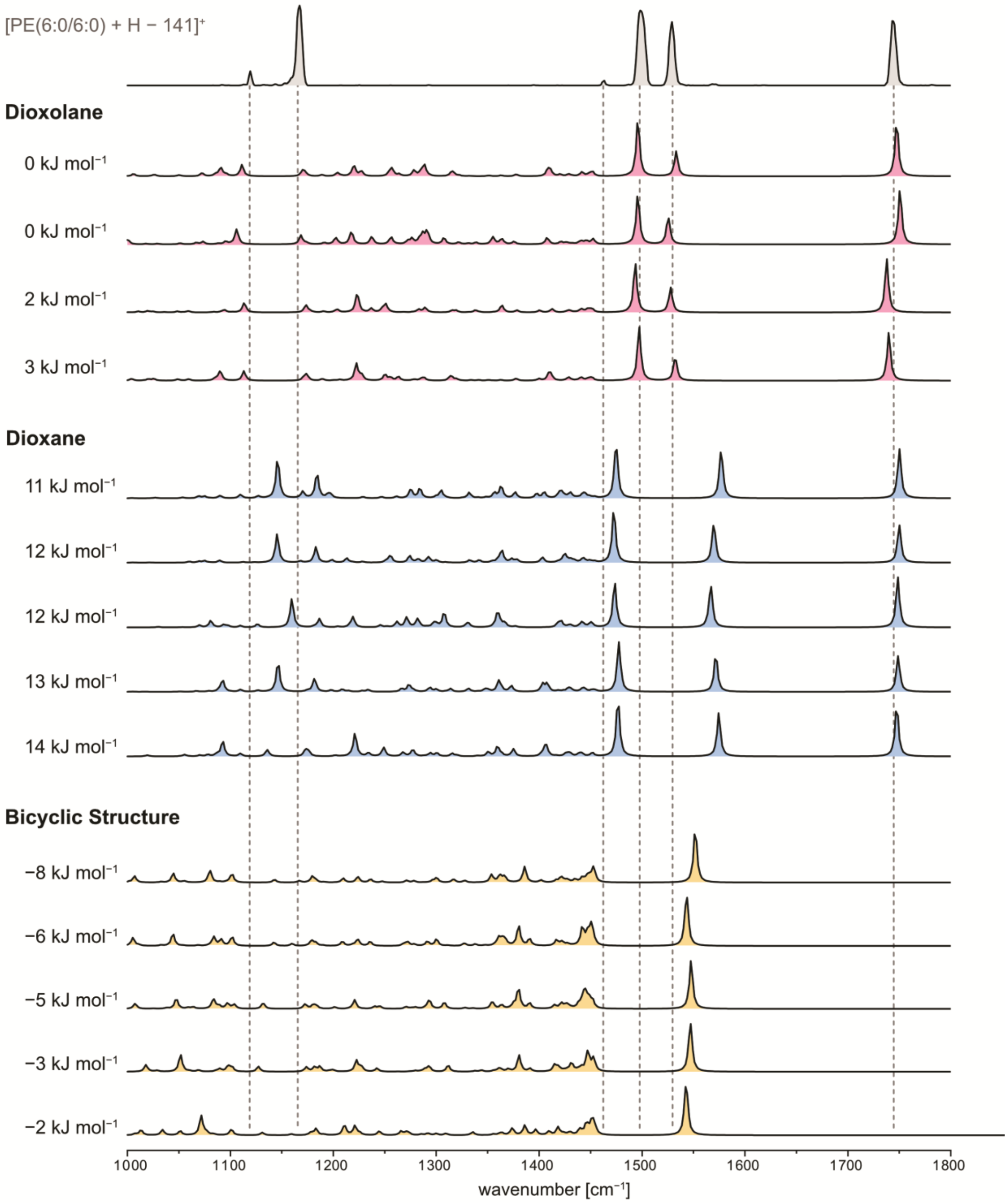

Figure S5. Computed IR spectra and relative free energies at 90 K of calculated conformers of dioxolanes, dioxanes and bicyclic structures equipped with hexanoic acid chains (6:0/6:0) and protonated C=C bond. The experimental spectrum of [PE(6:0/6:0) + H − 141]$^+$ (gray) is well-modelled by the computed spectra of dioxolane structures. Spectra were computed at the PBE0+D3/6-311+G(d,p) level of theory and XYZ coordinates of all computed structures are listed on page 21ff.

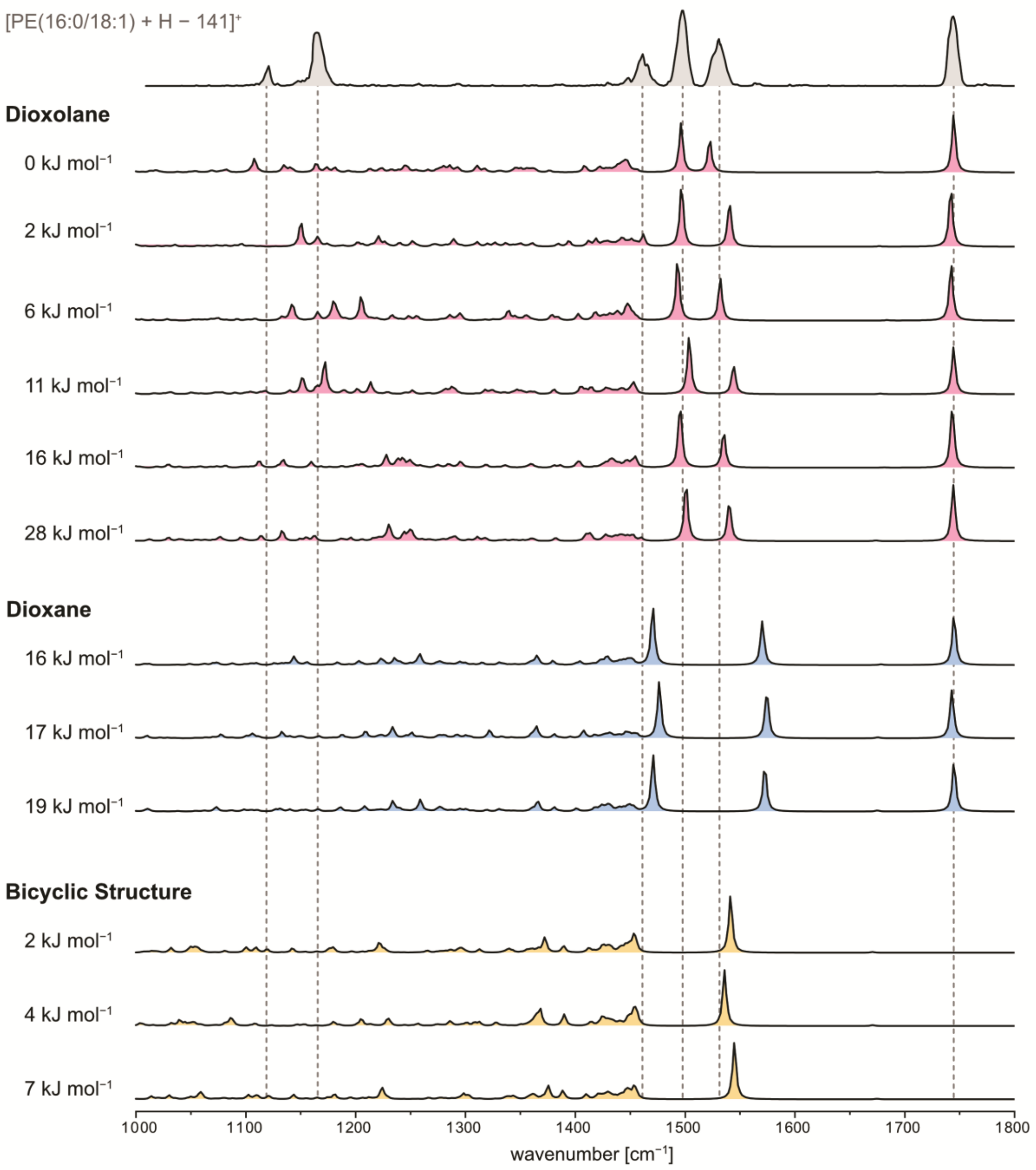

Figure S6. Computed IR spectra and relative free energies at 90 K of dioxolane, dioxane and bicyclic structures equipped with full-length lipid chains (16:0/18:1) and protonated C=C bond. The spectral signatures are largely unaffected by the lipid chains compared with the hexanoic acid residues (*cf*. Figure S5). The experimental spectrum of $[PE(16:0/18:1) + H - 141]^+$ (gray) is well modelled by the computed spectra of dioxolane structures, which also exhibit the lowest free energy. Spectra were computed at the PBE0+D3/6-311+G(d,p) level of theory and XYZ coordinates of all computed structures are listed on page 27ff.

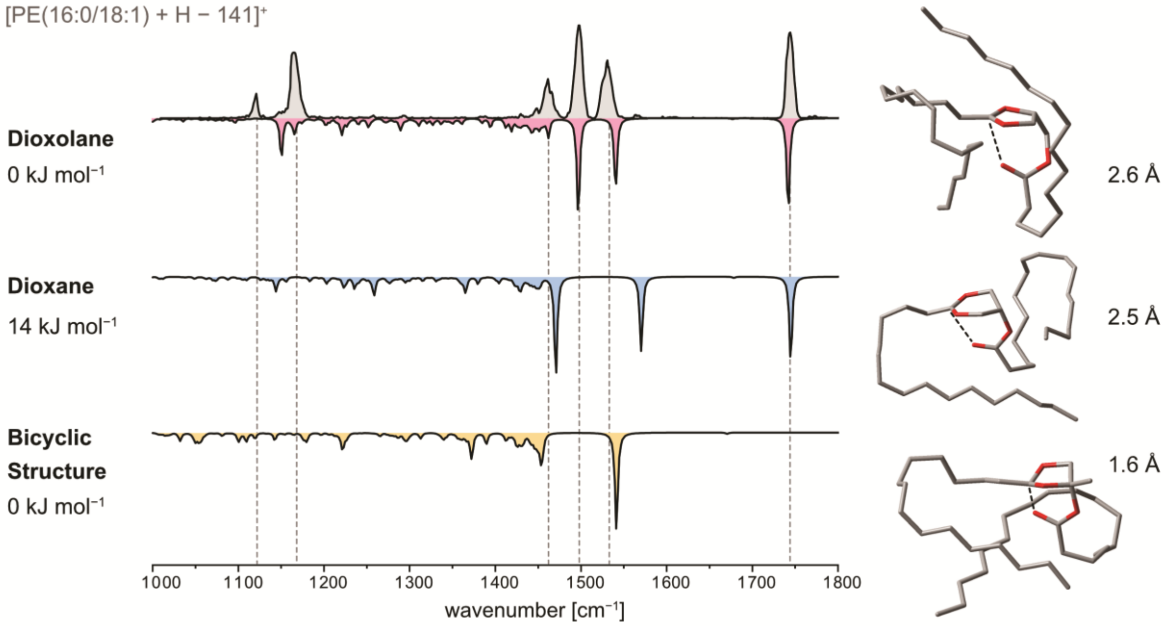

Figure S7. Computed IR spectra, structures and relative free energies of selected conformers with full-length lipid chains (16:0/18:1) and protonated C=C bond from Figure S6. The dioxolane ring and bicyclic structure are energetically comparable but only the dioxolane ring matches the experimental spectrum of [PE(16:0/18:1) + H − 141]$^{+}$ (gray). Dioxane structures are energetically disfavored relative to dioxolanes and the spectral signature does not match the main bands in the experimental spectrum. Computed spectra are depicted as inverted traces to allow for direct comparison with the experiment, and hydrogen atoms are omitted in the 3D structures for visibility of the core structure motifs. Distances between the carbonyl oxygen and sp^{2}-carbon are indicated.

Conversion Energy Barriers

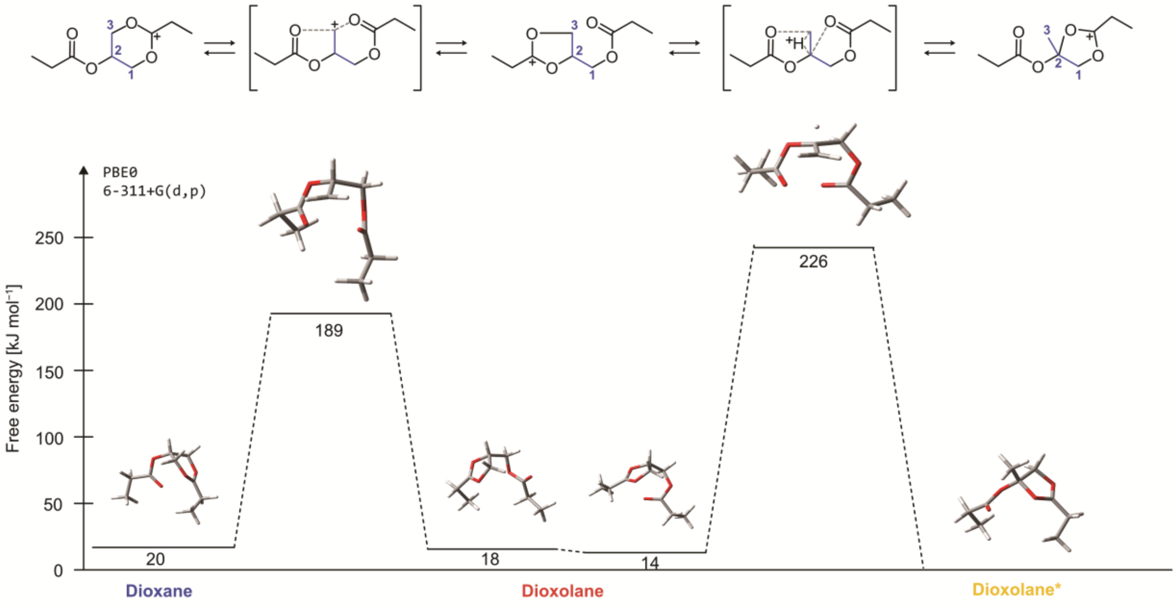

Figure S8. Energy diagram showing energy barriers for the interconversion between protonated dioxolane and dioxane rings computed at the PBE0+D3/6-311+G(d,p) level of theory. Bond rotation within the dioxolane marked by an asterisk yields the bicyclic structure. XYZ coordinates of all computed structures are listed on page 19ff.

CID of PE(d31-16:0/18:1)

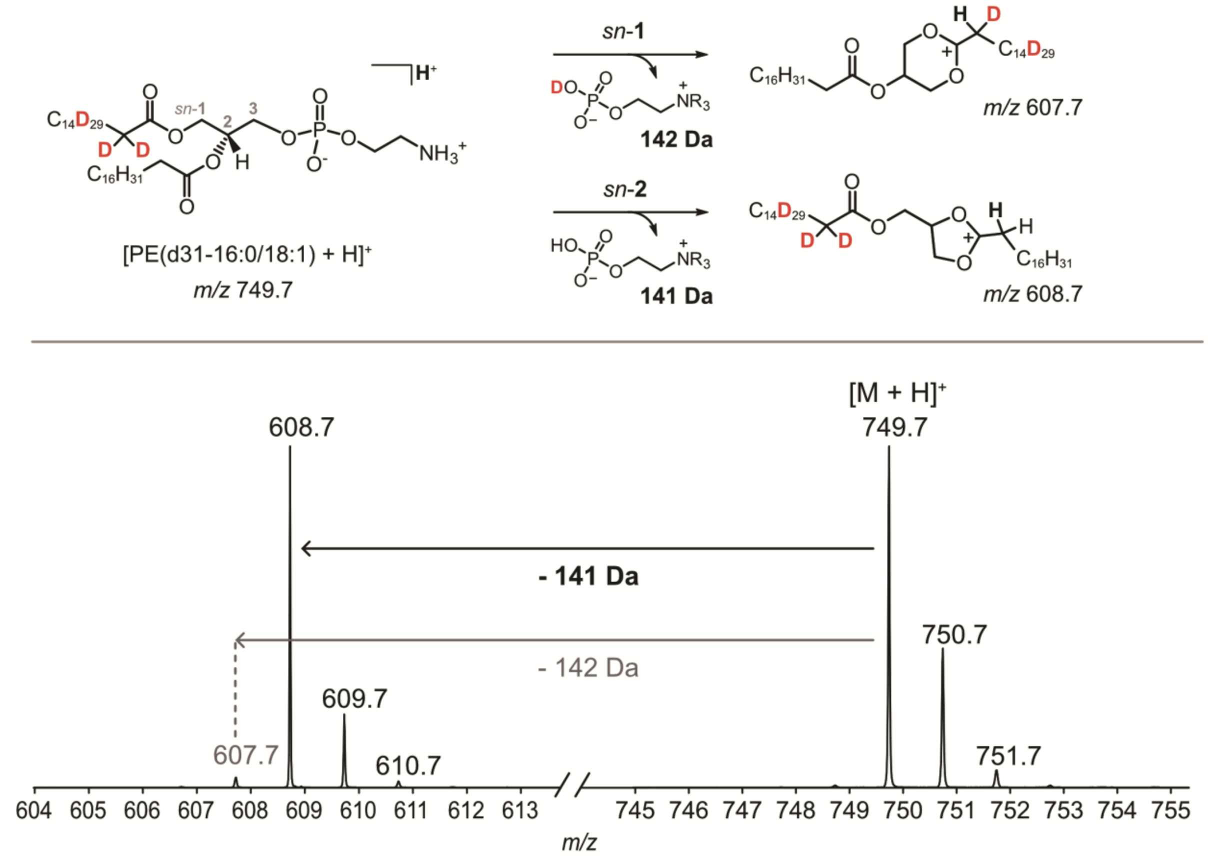

Figure S9. CID spectrum of protonated PE(d31-16:0/18:1) recorded on a Synapt G2-S HDMS instrument. The loss of 141 Da upon ion activation in the CID cell provides evidence that the alpha hydrogens on the fully deuterated *sn*-1 fatty acyl chain do not participate in the neutral headgroup loss. *Sn*-1 participation would result in a neutral loss of 142 Da, which is only observed to a negligible extent. The relative intensity of the peak at *m/z* 607 compared with the peak intensity at *m/z* 608 is approximately 0.03, in perfect accordance with the dioxane ratio of 2–3 % determined by IR spectroscopy.

Phosphatidylcholine Fragmentation Pathway

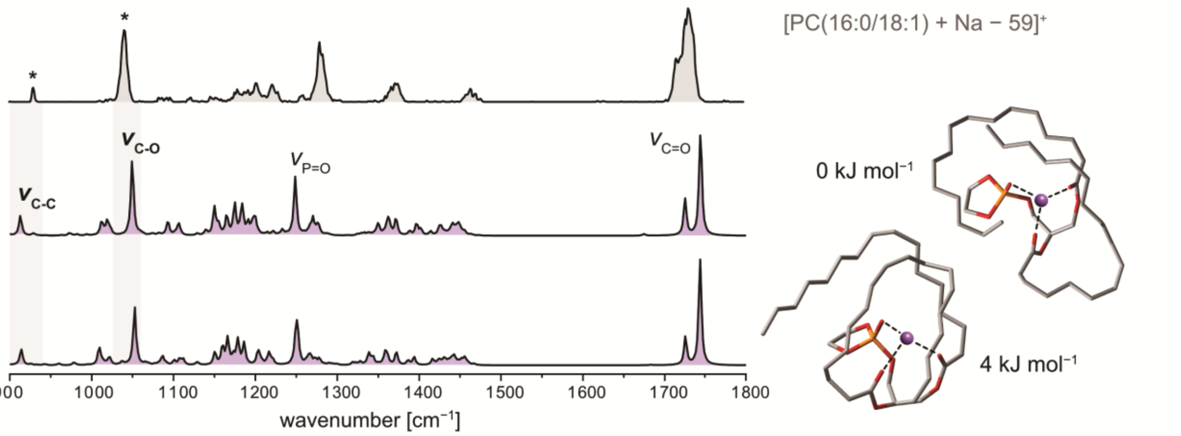

Figure S10. Experimental IR spectrum of [PC(16:0/18:1) + Na − 59]$^+$ (top) and computed spectra of two low-energy conformers featuring a cyclic phosphate. The C-C and C-O stretching vibrations marked by asterisks in the experimental spectrum are diagnostic for the phosphate ring. The corresponding 3D structures are depicted next to the computed spectra with hydrogens omitted for visibility. Relative free energies at 90 K are indicated in kJ mol^{-1} next to the corresponding structure. Spectra were computed at the PBE0+D3/6-311+G(d,p) level of theory and XYZ coordinates of all computed structures are listed on page 38ff.

Neutral Fatty Acid Loss from Diacylglycerols

Computed IR Spectra

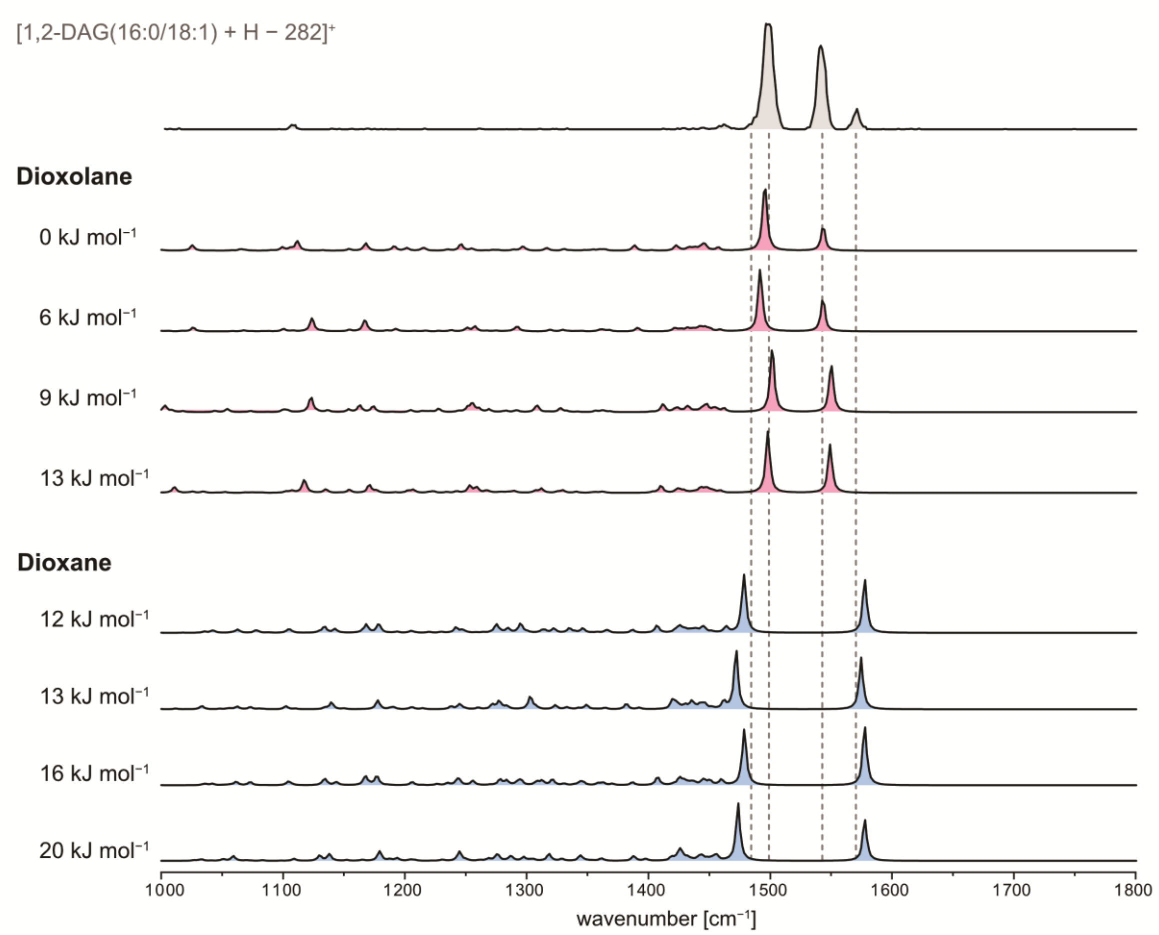

Figure S11. Computed IR spectra and relative free energies at 90 K of calculated conformers of protonated dioxolanes and dioxanes equipped with a palmitic acid chain (*m/z* 313). The 1,2-DAG(16:0/18:1) precursor yields mainly dioxolane-type fragments upon neutral loss of oleic acid. Dioxane-type fragments are discernible in the experimental IR spectrum at a low ratio. Spectra were computed at the PBE0+D3/6-311+G(d,p) level of theory and XYZ coordinates of all computed structures are listed on page 40ff.

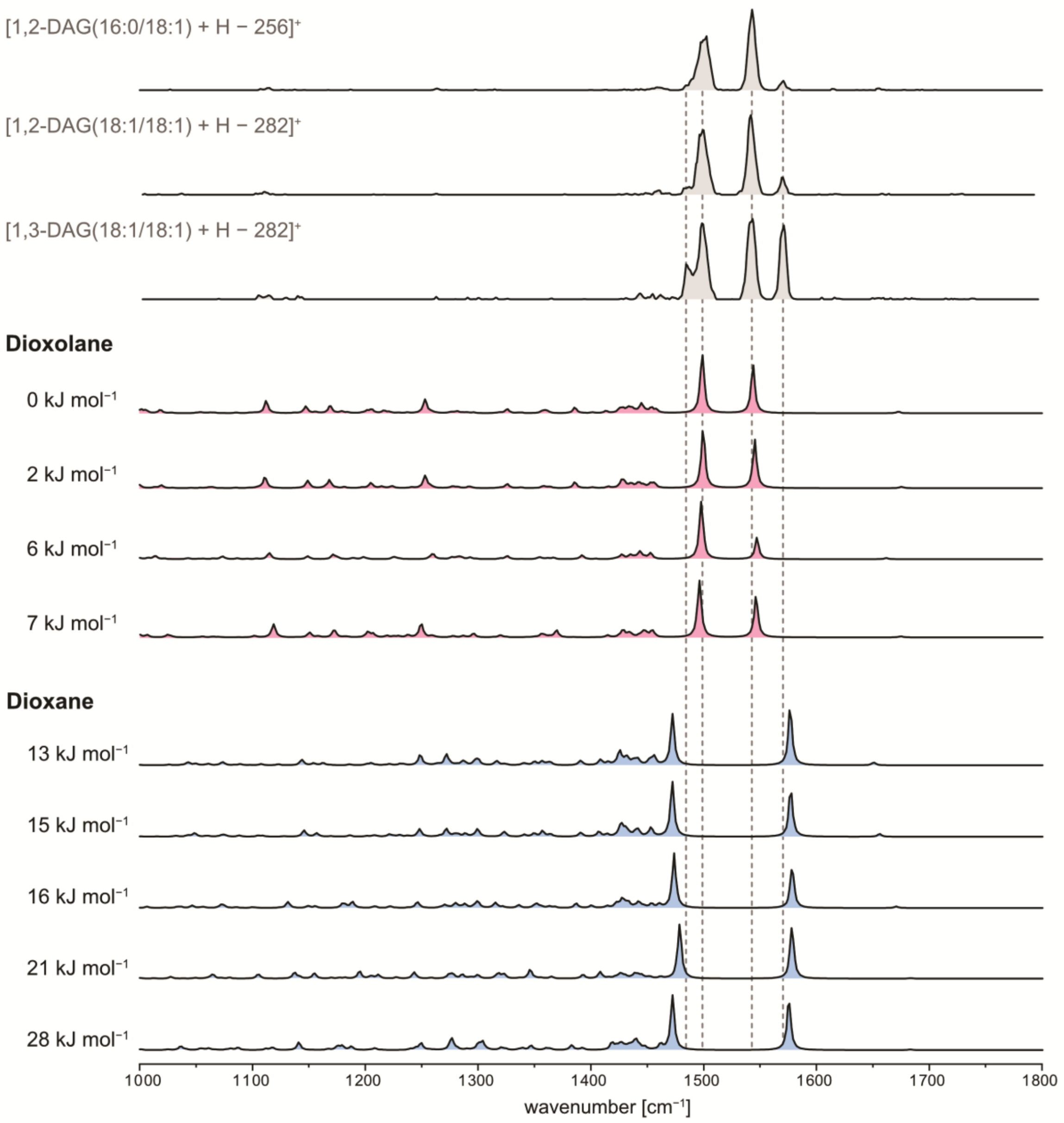

Figure S12. Computed IR spectra and relative free energies at 90 K of calculated conformers of protonated dioxolanes and dioxanes equipped with an oleic acid chain (*m/z* 339). The 1,2-DAG(16:0/18:1) and 1,2-DAG(18:1/18:1) precursors yield identical fragments. Fragmentation of the 1,3-DAG(18:1/18:1) regioisomer results in an increased ratio of dioxane fragments. Spectra were computed at the PBE0+D3/6-311+G(d,p) level of theory and XYZ coordinates of all computed structures are listed on page 45ff.

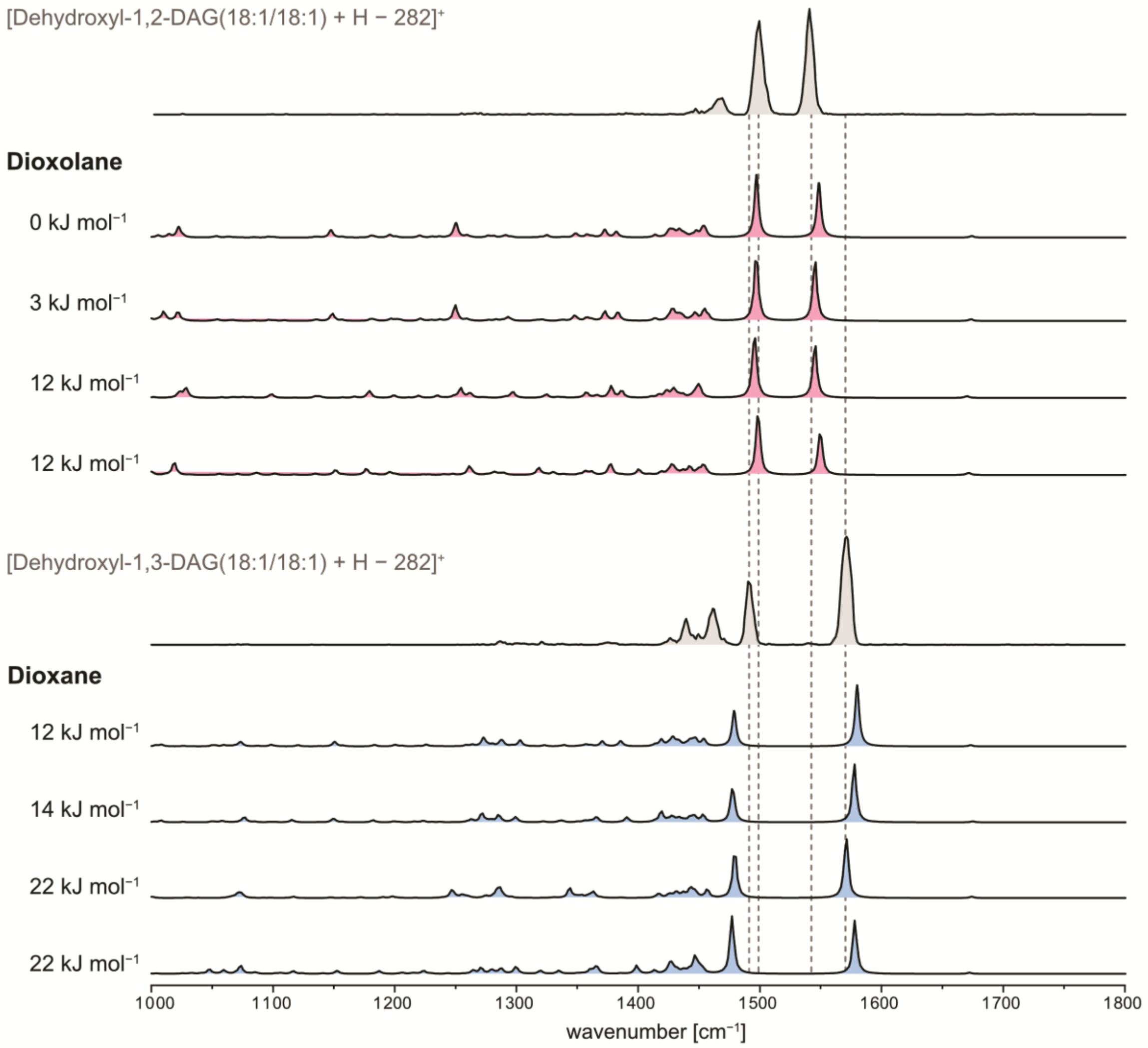

Figure S13. Computed IR spectra and relative free energies at 90 K of calculated conformers of protonated dioxolanes and dioxanes derived from dehydroxyl derivatives of 1,2- and 1,3-DAG equipped with an oleic acid chain (*m/z* 323). Dehydroxyl-1,2-DAG yields dioxolane structures, whereas fragments generated from dehydroxyl-1,3-DAG form dioxane rings. Spectra were computed at the PBE0+D3/6-311+G(d,p) level of theory and XYZ coordinates of all computed structures are listed on page 50ff.

Synthesis of Propylene Glycol Dioleate

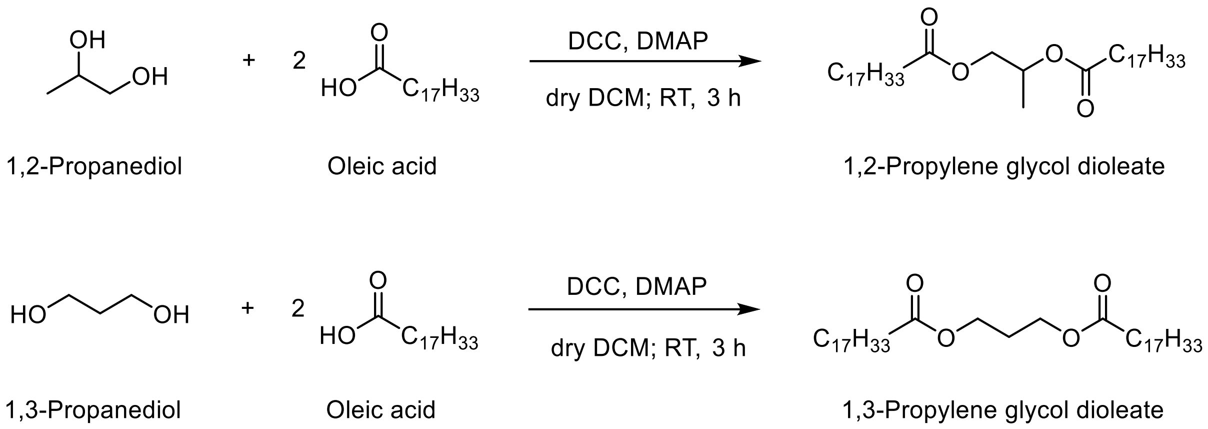

Scheme S1. Synthesis of 1,2- and 1,3-propylene glycol dioleate from propanediol and oleic acid, activated by *N*,*N′*-dicyclohexylcarbodiimide (DCC) and 4-dimethylaminopyridine (DMAP). The reaction was carried out in dry dichloromethane (DCM) under nitrogen atmosphere.

XYZ Coordinates of Computed Conformers

In the following, xyz coordinates of all computed conformers optimized at the PBE0+D3/6-311+G(d,p) level of theory and corresponding to the computed IR spectra shown in this work are listed. Each conformer is uniquely identifiable by its name and relative free energy (90 K). The name "conf_x" refers to the position x of the conformer in the CREST output file from the conformer sampling. The protomers are sorted and numbered by ascending free energy. The charge and multiplicity of all conformers is 1.

PE(3:0/3:0) + H − 141]$^+$

Figure S3

DIOXOLANE

protomer_0 0.0 kJ mol^{-1}

```
C  2.81517900  -0.73076400   0.82637100
C  1.54794300  -0.30726900   0.16291500
O  0.56622100  -0.98860200  -0.01452700
O  1.60532900   0.97635700  -0.26339000
C  0.61878100   1.41904900  -1.18556100
C -0.76840400   1.68465200  -0.61101600
H -1.25300900   2.47652600  -1.18087800
C -0.86423900   1.89143400   0.89976400
H -1.56935600   2.66681200   1.19665600
H  0.09824400   2.03129600   1.38753300
O -1.40791100   0.61883100   1.35869800
C -1.79588500  -0.05745100   0.33950600
O -1.62373200   0.50360800  -0.79560200
C -2.46167300  -1.35654000   0.48878500
C -2.52024300  -2.17789500  -0.78859000
H -3.02826800  -3.12030800  -0.58232100
H -1.51422900  -2.39897100  -1.14742200
H -3.07214700  -1.66085300  -1.57524400
H -3.46496100  -1.13169200   0.88013200
H -1.93805900  -1.87231200   1.30008800
H  1.01384600   2.35974300  -1.57105700
H  0.52221000   0.71116000  -2.01388300
H  3.15309900   0.07325700   1.48609300
C  3.89758900  -1.03913400  -0.21362200
H  4.13722700  -0.15565400  -0.80882400
H  3.58230000  -1.84136400  -0.88464500
H  4.80800500  -1.36299800   0.29305900
H  2.59155900  -1.61267400   1.42796400
```

protomer_1 65.6 kJ mol^{-1}

```
C  3.50150800  -0.17480100   0.81422800
C  2.97539900   0.00225600  -0.58410300
C  3.18330000  -1.57675800   1.34253400
O  1.67473500   0.45670200  -0.73750400
O  3.56994300  -0.26448000  -1.57726100
C  0.89177300   0.85492100   0.35125800
C -0.45436200   1.21442100  -0.22143200
C -1.48810400   1.71205500   0.79132800
O -1.10643500  -0.00222600  -0.74530600
O -2.61780000   0.81059000   0.58132700
C -2.28392000  -0.09291100  -0.26274900
C -3.20782500  -1.16022800  -0.67170900
C -4.39539100  -1.36599200   0.25613300
H  3.12320900   0.59257700   1.49563800
H  4.58258000  -0.04488200   0.74628200
H  2.10712400  -1.75769200   1.42726800
H  3.62330700  -1.71266500   2.33204000
H  3.59785400  -2.33647400   0.67717500
H  1.28833500   1.75362600   0.84471400
H  0.77970700   0.06591800   1.10642200
H -0.33444200   1.86722600  -1.08681400
H -1.84439600   2.72364300   0.60873100
H -1.18361400   1.59612800   1.83248900
H -2.60447500  -2.06361900  -0.81074400
H -4.99975900  -0.46194700   0.34347700
H -4.07417300  -1.66908500   1.25437100
H -5.02672700  -2.15722400  -0.14876400
H -3.53234300  -0.87799200  -1.68525800
```

protomer_2 124.3 kJ mol^{-1}

```
C  2.33095800   0.30507900  -0.44003300
C  1.02164900  -0.01061000   0.26113000
O  1.17463400  -0.66643800   1.46101900
H  1.94539900  -1.24319500   1.40898200
O  0.26369500  -0.81547900  -0.68671900
C -0.66118500  -1.79560800  -0.23633100
H -0.27167300  -2.34499800   0.62424800
H -0.80411800  -2.48765000  -1.07014400
C -1.99077500  -1.15539900   0.15728100
H -2.69284700  -1.83817200   0.63120500
C -2.59310400  -0.22576600  -0.88503500
H -2.45766000  -0.54775500  -1.91556300
O -1.74372700   0.95910100  -0.70465200
C -1.12452200   0.80173200   0.40404500
C  0.21126300   1.35009100   0.62682800
C  0.51730800   2.58444100  -0.19867700
H  1.54494400   2.90506700  -0.03225700
H  0.37234200   2.40953300  -1.26584900
H -0.13538900   3.40592200   0.10447900
H  0.38345100   1.49611400   1.69446700
O -1.55924700  -0.15514200   1.15327300
H -3.62537200   0.06299300  -0.68733400
H  2.10008500   0.77385300  -1.39877200
H  2.87261200   1.03837900   0.16473900
C  3.18880100  -0.93324300  -0.66437000
H  4.08155600  -0.66010700  -1.22971600
H  3.55065600  -1.37622700   0.27117100
H  2.65081600  -1.69440100  -1.23206100
```

Dioxane

protomer_0 0.0 kJ mol^{-1}

C	2.90292200	-1.17499800	-0.55973800
C	1.58048000	-0.52591600	-0.32153000
O	0.55803500	-1.07696100	0.00519300
O	1.64718100	0.81712100	-0.48446300
C	0.50297800	1.62729900	-0.23582400
H	0.89632900	2.63611700	-0.37955600
C	-0.62644800	1.45352500	-1.22965600
H	-0.26472900	1.16095000	-2.21365100
H	-1.20813900	2.37356600	-1.31507000
O	-1.57089300	0.41660700	-0.85514200
C	-1.69233400	0.03469700	0.35566100
O	-1.11953600	0.59901900	1.34878100
C	-0.04522900	1.56194700	1.17653500
H	0.71556400	1.26664600	1.89760300
H	-0.46753900	2.52408100	1.47038000
C	-2.58868000	-1.09945300	0.64874000
C	-3.54866300	-1.47328200	-0.46729200
H	-3.01548400	-1.74994300	-1.37777900
H	-4.14412700	-2.33043200	-0.15120500
H	-4.23381400	-0.65557500	-0.69985000
H	-1.89565200	-1.92344900	0.87402200
H	-3.09399000	-0.87396400	1.59301100
H	3.31393400	-0.79372100	-1.49875100
H	2.72053600	-2.24507300	-0.66486500
C	3.87665700	-0.89409500	0.58843800
H	4.08129100	0.17427700	0.68292900
H	3.48480900	-1.26512300	1.53872600
H	4.82260600	-1.40298700	0.39710100

protomer_1 45.5 kJ mol^{-1}

C	-3.07213500	0.50784800	0.62844900
C	-1.95306900	1.02470100	-0.23094100
O	-0.88882800	0.16166800	-0.49258200
O	-1.92086900	2.08042200	-0.77164400
C	-3.96725400	-0.46478900	-0.14521900
C	0.25949400	-0.55114100	1.47452400
C	0.22942800	-1.87329900	-0.57851900
O	1.49295500	0.04102400	0.96987900
O	1.48639100	-1.20710900	-0.90890400
C	1.96848200	-0.28353500	-0.16979500
C	3.14057500	0.45522600	-0.67962300
C	3.92516500	1.22790700	0.36763200
C	-0.59671300	-0.96573600	0.29773100
H	-3.64510400	1.38491700	0.93101500
H	-2.69389300	0.04207900	1.54510800
H	-4.37595200	0.01845500	-1.03468100
H	-4.80184300	-0.78125300	0.48249900
H	-3.43363800	-1.36277400	-0.47098200
H	0.54074900	-1.38916800	2.11505000
H	-0.18960700	0.23921600	2.07344100
H	-0.24223300	-2.05354100	-1.54211800
H	0.48627000	-2.81662200	-0.09431700
H	3.75330200	-0.26074000	-1.23596900
H	3.30505400	1.97492000	0.86464900
H	4.75125900	1.74581700	-0.12055600
H	4.34551800	0.56287000	1.12465400
H	-1.48243900	-1.50630000	0.64433500
H	2.72153700	1.13099100	-1.44153900

protomer_2 102.4 kJ mol^{-1}

C	-3.15557600	-0.99395300	-0.37766700
C	-1.82371800	-0.56748300	0.11561700
O	-1.04041300	-1.45852700	0.54965800
H	-0.09974300	-1.12913000	0.84534000
O	-1.57270800	0.69411600	0.07152300
C	-0.32589600	1.40979200	0.38772800
H	-0.69035200	2.41586200	0.59882900
C	0.42376200	0.86238700	1.58334800
O	1.06759900	-0.37173300	1.23284600
C	1.89335000	-0.24075500	0.10203700
C	3.16927800	-0.58305900	0.14299900
C	4.09280200	-0.54019700	-1.02165800
H	3.60384500	-0.15263100	-1.91557800
H	4.96364500	0.08174600	-0.79457800
H	4.46963900	-1.54406900	-1.24052500
H	3.53634600	-0.94481100	1.09848600
O	1.19481700	0.14737400	-0.99729800
C	0.57000100	1.41341200	-0.83565800
H	0.00065400	1.60773100	-1.74425700
H	1.32579900	2.19887800	-0.71081300
H	-0.22274000	0.65364800	2.43725900
H	1.18321300	1.59000900	1.88373100
C	-4.11743700	0.11794900	-0.75086900
H	-3.72116900	0.74192500	-1.55365700
H	-5.05383400	-0.32074100	-1.09773000
H	-4.34336400	0.75804300	0.10404300
H	-2.94297900	-1.65356300	-1.23051500
H	-3.56271900	-1.66129800	0.39085900

Open Chain

protomer_0 0.0 kJ mol^{-1}

C	2.19312600	-1.44924500	-0.31474000
C	1.13670100	-0.48006500	0.08540400
O	0.86843900	0.52775100	-0.81292800
O	1.23941100	0.04776400	1.31821100
C	0.54436100	1.30335900	1.30659700
C	-0.01361900	1.34835800	-0.10764000
O	-1.31824400	0.62264900	-0.10459400
C	-1.29085400	-0.66640600	-0.06010200
O	-0.21649500	-1.32001300	0.08498500
C	-2.56965300	-1.39658100	-0.20317700
C	-3.81836500	-0.57729000	0.08118900
H	-4.69729700	-1.20589000	-0.06573300
H	-3.89979900	0.27985300	-0.58852100
H	-3.83309700	-0.21635600	1.11161900
H	-2.56634500	-1.76746700	-1.23915400
H	-2.49412700	-2.29022700	0.42354000
C	-0.24894600	2.66420200	-0.76598500
H	-0.64332400	2.51324200	-1.77150900
H	-0.96206800	3.25071400	-0.18410300
H	0.69585200	3.20621100	-0.82844600
H	1.26236000	2.10778400	1.47464300
H	-0.22651700	1.31390800	2.07727000
H	1.90145700	-1.87766300	-1.27669400

C	3.56083500	-0.78157900	-0.38962200
H	3.57429200	0.01850200	-1.13256600
H	3.85329600	-0.37049900	0.57839300
H	4.30714500	-1.52190300	-0.68070400
H	2.18176500	-2.25392500	0.42503300

protomer_1 54.0 kJ mol^{-1}

C	2.48988500	-1.15626800	-0.55893400
C	1.85432000	0.14141500	-0.23920800
O	1.23433100	0.75510600	-1.16154200
C	0.42533600	1.94969900	-0.85389300
C	-0.70095900	1.62543200	0.06385000
C	-0.95714400	2.28514300	1.18176600
H	-1.85268900	2.07882000	1.75572000
H	-0.28632300	3.05870500	1.53429700
O	-1.59529200	0.67038500	-0.40887000
C	-1.34172300	-0.63449900	-0.13922100
O	-0.29977200	-0.99990700	0.35298100
C	-2.49475100	-1.50396900	-0.51491300
C	-3.72190000	-1.23558100	0.35904600
H	-4.07056200	-0.20764300	0.24470400
H	-4.53287800	-1.90377800	0.06522100
H	-3.50328200	-1.41806200	1.41401200
H	-2.73725900	-1.30696300	-1.56432100
H	-2.16265200	-2.53875000	-0.42422400
H	0.07237200	2.24488600	-1.84138000
H	1.07496000	2.71766100	-0.43604400
O	1.99558700	0.72767000	0.89432500
H	2.43449800	0.14464000	1.53425700
H	3.35568600	-0.91517400	-1.19207500
H	1.78782700	-1.70088900	-1.19332800
C	2.89767500	-1.95918300	0.66579500
H	2.03020400	-2.19510700	1.28722900
H	3.66478500	-1.45412300	1.26544300
H	3.34205100	-2.90338900	0.34961400

protomer_2 61.1 kJ mol^{-1}

C	2.79876600	-0.93887900	-0.69636900
C	1.54045000	-0.38410200	-0.11489300
O	0.58351000	-1.03563800	0.25447800
O	1.57734900	0.95309600	-0.01982000
C	0.53531300	1.59258200	0.72363200
C	-0.65793800	1.90932500	-0.11304200
C	-1.14980200	3.09320300	-0.41995000
H	-0.67089700	3.98665100	-0.03946100
H	-2.02659100	3.20209100	-1.04665600
O	-1.27001700	0.77404900	-0.73822800
C	-1.71776800	-0.20173900	-0.05114600
O	-1.91719800	-0.02037300	1.20332900
H	-2.21618300	-0.84103100	1.62759100
C	-2.12732400	-1.42996400	-0.76482500
C	-2.34485000	-2.62715700	0.14596600
H	-2.62982300	-3.49237100	-0.45330100
H	-3.16561800	-2.47273200	0.85698500
H	-1.42713700	-2.88433100	0.68073400
H	-1.36577800	-1.62132000	-1.52347900
H	-3.04433500	-1.16252100	-1.30975000
H	0.27661600	0.97421700	1.58556800
H	0.95585800	2.53337300	1.07606200
H	2.60762000	-1.98097100	-0.95528400
C	3.97012100	-0.81983600	0.28226800
H	4.86748400	-1.24720300	-0.16794300
H	3.76813200	-1.36299700	1.20855400
H	4.17622100	0.22415900	0.52529400
H	3.02346800	-0.38469000	-1.61281900

PE(3:0/3:0) + H − 141]$^+$ Transition States

Figure S8

Dioxane 20.0 kJ mol^{-1}

C	3.15145400	0.48487800	0.14865000
C	1.72146600	0.06863300	0.04181700
O	0.78477100	0.77559900	-0.23535200
O	1.57013100	-1.25317300	0.29823000
C	0.28073700	-1.85325000	0.23204600
H	0.50368800	-2.90487300	0.42572000
C	-0.68584200	-1.40623100	1.30884100
H	-0.17389600	-1.11540000	2.22430400
H	-1.40645200	-2.19582900	1.53087900
O	-1.47064900	-0.24324300	0.93430300
C	-1.64885000	0.07305700	-0.28836900
O	-1.29163100	-0.65147300	-1.27828900
C	-0.39906200	-1.78723500	-1.12176600
H	0.31777600	-1.69583200	-1.93630100
H	-1.01966600	-2.66990800	-1.28396400
C	-2.35669400	1.33072300	-0.59375400
C	-3.09953000	1.95739800	0.57328400
H	-2.42406600	2.19515300	1.39611700
H	-3.56568000	2.88534400	0.24036400
H	-3.88749400	1.30122900	0.94832100
H	-1.55936100	1.99209700	-0.96341700
H	-3.00001200	1.13537900	-1.45748000
H	3.72228800	-0.14491400	-0.54363100
H	3.49843700	0.19426200	1.14698000
C	3.36569900	1.96237700	-0.11998500
H	3.03896200	2.23431900	-1.12546600
H	2.81282000	2.57929600	0.59104200
H	4.42541400	2.20589700	-0.02919600

Transition state_1 188.5 kJ mol^{-1}

C	-2.47872100	1.43542900	-0.57414800
C	-1.55093000	0.27781900	-0.66819100
O	-0.54074600	0.21774100	-1.34505400
O	-1.90433800	-0.77531300	0.08805500
C	-0.89109300	-1.76393900	0.17110400
H	-1.41357700	-2.71856800	0.35912500
C	-0.17124800	-1.95328400	-1.08478300
H	-0.66193600	-1.83361100	-2.04321100
H	0.69202000	-2.60623200	-1.07151300
O	1.77937900	-0.88991500	-0.52283500
C	1.62463000	-0.08045800	0.37144700
O	0.68383600	-0.25120100	1.31509800
C	0.03399900	-1.50980200	1.36797800

H	-0.56160500	-1.48422600	2.27890100
H	0.76972900	-2.31813800	1.42395900
C	2.41286500	1.16895900	0.55406200
C	3.29267100	1.50793600	-0.63482400
H	2.69572600	1.66605800	-1.53522400
H	3.85275000	2.42167600	-0.43101400
H	4.00663400	0.70848000	-0.83920100
H	1.70275500	1.96880400	0.79163400
H	3.00218700	1.02955100	1.46965800
H	-3.48247800	1.06546900	-0.81046700
H	-2.18493500	2.15255600	-1.34104000
C	-2.46475700	2.06024500	0.82353900
H	-2.79083500	1.34643300	1.58168200
H	-1.46855400	2.42293600	1.08637600
H	-3.14742400	2.91089600	0.84111300

Dioxolane_1 17.7 kJ mol^{-1}

C	2.59261600	1.58738300	0.05801800
C	1.99272400	0.26369800	0.26337700
O	1.37251100	-0.03723400	1.34062100
O	2.02341100	-0.64144500	-0.63823000
C	1.11790600	-1.73806300	-0.23684400
H	1.66203400	-2.66632900	-0.40922100
C	0.90635500	-1.42223300	1.24386600
H	1.53087700	-2.00835800	1.91722000
H	-0.13794300	-1.44522200	1.55034900
O	-2.16799900	-1.18639300	0.51342000
C	-1.84343300	-0.29316100	-0.21739500
O	-0.71890700	-0.37694500	-1.00197800
C	-0.10902400	-1.65431100	-1.11217000
H	0.17294000	-1.78292300	-2.15864600
H	-0.80413400	-2.44575800	-0.81658000
C	-2.54463500	1.01049400	-0.42078800
C	-3.64223500	1.26198700	0.59630200
H	-3.24050600	1.31784000	1.61047400
H	-4.14653500	2.20460300	0.37726600
H	-4.38488400	0.46291800	0.57668500
H	-1.79085800	1.80508200	-0.42677000
H	-2.94283700	0.98797900	-1.44309200
H	3.36903300	1.49203800	-0.70287900
H	3.04082800	1.90939400	1.00079800
C	1.50457200	2.58287000	-0.38947700
H	1.02495800	2.25457400	-1.31300400
H	0.74738500	2.70982700	0.38599500
H	1.97845000	3.54849200	-0.56859600

Dioxolane_2 13.7 kJ mol^{-1}

C	-2.81499700	0.73049500	0.82649300
C	-1.54780800	0.30681400	0.16297200
O	-0.56597900	0.98814700	-0.01418800
O	-1.60536900	-0.97656200	-0.26358400
C	-0.61861400	-1.41954800	-1.18548700
C	0.76864900	-1.68455900	-0.61097400
H	1.25325200	-2.47651400	-1.18074800
C	0.86458500	-1.89125000	0.89986100
H	1.56975900	-2.66658700	1.19674000
H	-0.09780900	-2.03105500	1.38781800
O	1.40831700	-0.61860700	1.35861000
C	1.79600600	0.05759900	0.33927700
O	1.62371600	-0.50361000	-0.79577600
C	2.46205600	1.35653800	0.48827000
C	2.51808700	2.17900000	-0.78848200
H	3.02678600	3.12110000	-0.58244700
H	1.51135300	2.40060700	-1.14497100
H	3.06816300	1.66253300	-1.57679800
H	3.46623600	1.13111800	0.87714000
H	1.94051800	1.87163500	1.30130600
H	-1.01348300	-2.36059800	-1.57032600
H	-0.52242100	-0.71217800	-2.01430000
H	-3.15305700	-0.07350500	1.48618300
C	-3.89740300	1.03920000	-0.21337200
H	-4.13728600	0.15584500	-0.80866800
H	-3.58200200	1.84143800	-0.88433800
H	-4.80773000	1.36320300	0.29338900
H	-2.59113200	1.61228400	1.42817500

Transition state_2 225.7 kJ mol^{-1}

C	2.88851200	-0.63876400	0.87432600
C	1.62285500	-0.31875500	0.15428500
O	0.66199900	-1.02503500	0.00674100
O	1.65292800	0.93500800	-0.39595200
C	0.58208100	1.22528400	-1.26962400
C	-0.71506600	1.52703400	-0.51907500
O	-1.08460400	2.63093800	-0.85287800
C	-0.74480500	2.33404300	0.62689500
O	-1.68288200	2.54831000	1.12557600
C	0.13867300	2.91356000	0.89446600
C	-1.50036200	0.29395700	1.56379400
H	-1.96101000	-0.09559000	0.53106600
H	-1.73465400	0.64100400	-0.65508500
H	-2.82264900	-1.27895000	0.28013600
H	-2.30515700	-2.21042700	-0.81428700
H	-2.99132900	-3.05328200	-0.90933100
C	-1.31526800	-2.59308000	-0.56511200
H	-2.25457600	-1.70293000	-1.77856800
H	-3.80620000	-0.87764400	0.00165600
H	-2.93069000	-1.79021200	1.23737200
H	0.90182800	2.09395800	-1.84581600
H	0.37424400	0.39168800	-1.94321700
H	3.18637700	0.23010100	1.46774800
C	4.00259800	-1.00649700	-0.11118200
H	4.23263600	-0.17380500	-0.77901500
H	3.72407200	-1.87397100	-0.71369300
H	4.90927200	-1.25800000	0.44124900
H	2.67501300	-1.46936400	1.54834800

Open chain 0.0 kJ mol^{-1}

C	2.83772400	1.16720400	0.11301500
C	1.84662500	0.09520900	0.28933200
O	0.96845200	-0.17445100	-0.59124300
O	1.83815100	-0.63763900	1.34297000
C	0.82951700	-1.67369300	1.18503200
C	0.04014300	-1.23399300	-0.05137900
H	-0.80706900	-3.06258200	-0.69861400
C	-0.15625100	-2.28449400	-1.10637600
H	-0.61709400	-1.86049100	-1.99553800

H	0.80223300	-2.73489700	-1.37028600
O	-1.65390700	0.19442900	-1.64640400
C	-1.93559500	0.08402500	-0.49663900
O	-1.08910400	-0.58714600	0.40449800
C	-3.11851600	0.62504600	0.23263900
C	-2.73617000	1.77312500	1.17002700
H	-3.63266100	2.16232400	1.65502700
H	-2.27309300	2.59445400	0.61757800
H	-2.04926700	1.44014600	1.95099800
H	-3.57699000	-0.18880000	0.80249700
H	-3.82670900	0.95953900	-0.52640100
H	1.35739500	-2.61707000	1.04991800
H	0.21637300	-1.68216600	2.08318500
H	3.07570500	1.55184100	1.10946800
C	2.41322700	2.25151700	-0.86766800
H	1.52037600	2.77347100	-0.51832500
H	2.20887800	1.84011800	-1.85704200
H	3.21748700	2.98172300	-0.95996300
H	3.75147200	0.65717700	-0.22896600

PE(6:0/6:0) + H − 141]$^+$

Figure S5

DIOXOLANE

conf_219 0.0 kJ mol^{-1}

C	-1.14568500	-1.52509400	-1.35704100
C	0.33243700	-1.40157600	-1.22245500
O	1.00596100	-0.46334500	-1.57361000
O	0.87678200	-2.45190500	-0.56190900
C	2.29282400	-2.48446500	-0.44274500
C	2.86773200	-1.57644300	0.63711100
C	1.90385100	-1.14725600	1.74091200
O	1.57940900	0.22346300	1.35777100
C	2.39497600	0.60511600	0.44503600
O	3.24825700	-0.27336000	0.06946100
C	2.38511300	1.95624900	-0.12171300
C	1.51442000	2.98618900	0.59566300
C	0.00574000	2.78549800	0.46490300
C	-0.51041100	2.76454900	-0.96961400
C	-2.03115400	2.74501600	-1.03513200
H	-2.38456500	2.70858800	-2.06842400
H	-2.45860400	3.63793000	-0.56976200
H	-2.44163000	1.87535800	-0.51153600
H	-0.11710100	1.89003800	-1.50167800
H	-0.13424300	3.64704600	-1.50245700
H	-0.30444000	1.87063900	0.98327600
H	-0.47748800	3.60629800	1.00633400
H	1.78183600	3.95708700	0.16737800
H	1.79993400	3.03239200	1.65150600
H	2.07186700	1.80598200	-1.16487300
H	3.43553600	2.26550200	-0.18096400
H	2.35363400	-1.10098800	2.73194600
H	0.97204700	-1.70833300	1.75602800
H	3.79544300	-1.99993100	1.01988000
H	2.52226100	-3.51721500	-0.17682500
H	2.76875900	-2.24686800	-1.39788500
C	-1.82998100	-1.12349800	-0.04189200
C	-3.34385200	-1.05421200	-0.17859300
C	-4.03130500	-0.61898900	1.10880200
C	-5.54342400	-0.53189300	0.96753300
H	-6.01310400	-0.21805000	1.90277200
H	-5.82700600	0.18927400	0.19479900
H	-5.97088200	-1.50017700	0.69006200
H	-3.63343000	0.35661000	1.41762400
H	-3.77495900	-1.32153600	1.91177600
H	-3.60226700	-0.35508800	-0.98430600
H	-3.72849900	-2.03376800	-0.48894500
H	-1.56379400	-1.84314100	0.74160500
H	-1.45150000	-0.14422900	0.27720400
H	-1.41232500	-2.55131900	-1.62116600
H	-1.45643700	-0.85607700	-2.16173300

conf_87 0.4 kJ mol^{-1}

C	2.85850400	0.18247900	-1.44209700
C	1.58225000	0.89853300	-1.16208100
O	0.46791000	0.51418200	-1.42568700
O	1.78945200	2.05537300	-0.48869700
C	0.67544800	2.91282100	-0.28967800
C	-0.33573500	2.45879000	0.75887600
C	0.13311400	1.40111100	1.75818900
O	-0.53271200	0.19497700	1.28139000
C	-1.38493200	0.51923200	0.37889700
O	-1.46060200	1.76941400	0.11352000
C	-2.28360200	-0.45939800	-0.23001000
C	-3.71716300	-0.30084000	0.32144900
C	-4.65376900	-1.32548600	-0.30399100
C	-6.08018800	-1.19951900	0.21640000
C	-7.01865600	-2.22175700	-0.40732600
H	-8.03408100	-2.11214400	-0.01989700
H	-6.69043500	-3.24347900	-0.19365200
H	-7.06407800	-2.10666500	-1.49450200
H	-6.45037900	-0.18682400	0.01544800
H	-6.07909300	-1.31609900	1.30705100
H	-4.64868600	-1.20497800	-1.39458000
H	-4.27845900	-2.33642900	-0.10176800
H	-4.07492400	0.71242200	0.11180700
H	-3.70536000	-0.42438000	1.41020300
H	-1.89063400	-1.45623200	-0.02467300
H	-2.26860200	-0.28357400	-1.30918000
H	-0.20926600	1.57246100	2.77791000
H	1.20496000	1.21469200	1.73194200
H	-0.77785400	3.33025600	1.24036500
H	1.11536700	3.85368200	0.04273300
H	0.14631800	3.08266000	-1.23178500
C	3.42912000	-0.44574100	-0.16240800
C	4.70002100	-1.23859200	-0.43324400
C	5.28262500	-1.86979800	0.82451000
C	6.55294800	-2.66090000	0.55072100
H	6.95131100	-3.10312300	1.46701900
H	6.36731800	-3.47450100	-0.15718900
H	7.33240600	-2.02196100	0.12447200
H	4.53080300	-2.52623600	1.28074300
H	5.49007400	-1.08290700	1.56066700
H	4.48948900	-2.02260300	-1.17156500

H	5.44786600	-0.57876900	-0.89123400
H	3.64018400	0.34564500	0.56634300
H	2.67578200	-1.10747300	0.28429800
H	3.58251500	0.88772000	-1.85989500
H	2.64516700	-0.58833900	-2.18453800

conf_2 2.3 kJ mol^{-1}

C	2.53987400	0.22369600	-1.77961900
C	1.61546000	-0.29628200	-0.72868900
O	0.42285300	-0.10591300	-0.66866200
O	2.24955700	-1.08661000	0.16765400
C	1.57454400	-1.39482700	1.37762100
C	0.44618900	-2.41653300	1.28539400
C	0.43093000	-3.32317300	0.05542200
O	-0.64754800	-2.75631600	-0.74358600
C	-1.27752800	-1.88991200	-0.03619900
O	-0.84581200	-1.73127000	1.15722500
C	-2.46318700	-1.19526700	-0.54553300
C	-2.81088500	0.08881700	0.19899000
C	-4.03550800	0.76291800	-0.40407100
C	-4.40090800	2.05642900	0.31229100
C	-5.62033700	2.73669800	-0.29094000
H	-5.45219400	2.99247100	-1.34161400
H	-5.86155500	3.66039800	0.24008100
H	-6.50027800	2.08783000	-0.24346200
H	-3.54298100	2.73926200	0.28109200
H	-4.58354600	1.84219600	1.37251800
H	-3.85127900	0.97524300	-1.46522900
H	-4.89062800	0.07494400	-0.37189200
H	-1.94970000	0.76218200	0.15525300
H	-2.99184000	-0.13583400	1.25525000
H	-3.28603200	-1.92476000	-0.48892300
H	-2.28928300	-1.02664100	-1.61327800
H	0.16190300	-4.35653200	0.27040400
H	1.34095700	-3.27234500	-0.53895600
H	0.38088900	-2.96968900	2.22166500
H	2.35286700	-1.80512000	2.02237400
H	1.18313200	-0.48236000	1.83739900
C	3.82760500	0.87692200	-1.26763900
C	3.62755500	2.21391500	-0.56177400
C	2.90227400	2.14519900	0.77797200
C	2.85196000	3.49416200	1.48133100
H	2.33746700	4.23903100	0.86677500
H	2.32644600	3.43152300	2.43777600
H	3.85997000	3.86972600	1.67998300
H	3.40606200	1.41186400	1.42157900
H	1.87345500	1.78940000	0.63327000
H	4.61485700	2.66083100	-0.39859600
H	3.09370900	2.90348300	-1.22867700
H	4.35263700	0.17668700	-0.60970400
H	4.47452900	1.02759600	-2.13663500
H	1.95926500	0.91078400	-2.39886600
H	2.80053700	-0.63786900	-2.40797200

conf_0 3.3 kJ mol^{-1}

C	2.93460300	-0.05195400	-1.81199700
C	1.92013300	-0.25754300	-0.73566100
O	0.86867600	0.32796100	-0.62348500
O	2.27023400	-1.24820200	0.11820600
C	1.56514400	-1.34513500	1.34617100
C	0.15601700	-1.92343400	1.27532900
C	-0.19037900	-2.74301600	0.03286800
O	-1.03208800	-1.82803300	-0.72706300
C	-1.31798300	-0.81739300	0.01011100
O	-0.83347900	-0.84258900	1.19544200
C	-2.23965900	0.22735100	-0.43100200
C	-3.65360700	-0.03155800	0.13533500
C	-4.62678500	1.04414900	-0.32782100
C	-6.03382900	0.82348400	0.21334200
C	-7.00843100	1.89712000	-0.24733500
H	-8.00907100	1.71855600	0.15272800
H	-7.08647600	1.91984200	-1.33851300
H	-6.69049900	2.88999700	0.08491500
H	-6.00038100	0.80035900	1.30940900
H	-6.39325800	-0.16282400	-0.10458400
H	-4.26083600	2.02804000	-0.00866700
H	-4.65499200	1.06411600	-1.42440600
H	-3.60783200	-0.04947600	1.22926800
H	-4.00376000	-1.01624800	-0.19372300
H	-2.25224800	0.22967900	-1.52217100
H	-1.85137300	1.18344000	-0.07281800
H	-0.78766600	-3.63025800	0.23917600
H	0.67093900	-2.98698900	-0.58556100
H	-0.06889500	-2.44535700	2.20481800
H	2.17594300	-2.01301000	1.95518700
H	1.51845000	-0.36796500	1.83609700
C	4.38657500	0.08284400	-1.34294300
C	4.69741000	1.37398700	-0.59305700
C	4.03677800	1.51275200	0.77433600
C	4.49395800	2.76087000	1.51592000
H	5.57527900	2.74394900	1.68059900
H	4.26103600	3.66476500	0.94507100
H	4.01051200	2.85040600	2.49219500
H	4.26433900	0.62210200	1.37497300
H	2.94491300	1.55318000	0.66396400
H	5.78373200	1.43467600	-0.46103600
H	4.42340200	2.23450600	-1.21727100
H	4.65134400	-0.78419800	-0.72892800
H	5.01537900	0.03144000	-2.23648600
H	2.61388000	0.82114200	-2.38435300
H	2.85173400	-0.92210400	-2.47625100

Dioxane

conf_7 11.4 kJ mol^{-1}

C	3.63468400	0.80051200	-1.00550200
C	2.19880500	0.87715100	-0.60207800
O	1.30860900	0.17816000	-1.02099600
O	1.96719200	1.87307000	0.28614600
C	0.64986200	2.09836200	0.78034900
C	-0.42469300	2.33378300	-0.26453300
H	-0.01253300	2.69734800	-1.20463600
H	-1.16893200	3.04135000	0.10313900
O	-1.17563000	1.14188400	-0.62056200
C	-1.01210500	0.02745700	-0.01967500
O	-0.43079800	-0.09685300	1.11206000
C	0.17747900	1.04856000	1.76162700

H 0.99790100 0.63638600 2.34592500
H -0.57983900 1.46071600 2.43229300
C -1.58237400 -1.18205100 -0.63948300
H -0.90937800 -1.40688300 -1.47758100
C -3.01953600 -0.98784700 -1.13234000
C -3.99927500 -0.69537800 -0.00415800
C -5.43633600 -0.55593600 -0.48871600
C -6.41255500 -0.26075200 0.63965000
H -6.40417200 -1.05753900 1.38972700
H -7.43467300 -0.17057400 0.26522000
H -6.16294000 0.67692900 1.14675800
H -5.48756900 0.23905200 -1.24259800
H -5.73062800 -1.48013000 -1.00012000
H -3.94328600 -1.49481000 0.74627200
H -3.70898900 0.23139900 0.51436600
H -3.04572400 -0.18930900 -1.88017400
H -3.30832000 -1.90716600 -1.64977000
H -1.50037500 -1.99646900 0.08259100
H 0.76484200 3.02901300 1.34038400
H 4.24030600 1.39104100 -0.31443000
C 4.15132700 -0.63413800 -1.13764400
C 4.51899600 -1.28202600 0.19540700
C 3.36887900 -1.49350100 1.17771100
C 2.31782800 -2.49000600 0.70714100
H 1.83250400 -2.16566700 -0.21708600
H 2.77261500 -3.46693700 0.51844500
H 1.54059100 -2.63328100 1.46377500
H 3.79053300 -1.83881400 2.12763600
H 2.90081800 -0.52582300 1.41226300
H 4.98429500 -2.25253500 -0.01152700
H 5.29350900 -0.67378700 0.67795700
H 5.04308500 -0.60769500 -1.76942400
H 3.41107100 -1.23202800 -1.67805500
H 3.68439000 1.30098000 -1.98107900

conf_5 11.8 kJ mol^{-1}

C -2.99431900 1.37008300 -1.07404400
C -2.17046000 0.17222400 -0.73309100
O -1.01987100 -0.00816100 -1.04981100
O -2.87524000 -0.75417100 -0.04147100
C -2.25765700 -1.97423100 0.36094600
C -1.57202100 -2.77529500 -0.73069300
H -1.95778800 -2.54009600 -1.72153400
H -1.67592600 -3.84468500 -0.54427800
O -0.13814700 -2.54609500 -0.82217700
C 0.47856200 -1.76505100 -0.01958200
O 0.01420300 -1.40173700 1.11504200
C -1.31239900 -1.81304600 1.52977300
H -1.64246800 -1.03996000 2.22076400
H -1.19491600 -2.75846900 2.06457000
C 1.81587000 -1.29507100 -0.40835500
H 2.22733500 -2.01462700 -1.12201000
C 2.76850100 -0.99075900 0.74428700
C 4.08581700 -0.40072700 0.25093200
C 3.95197300 0.96899500 -0.40493200
C 5.29976100 1.56706300 -0.78085300
H 5.18342000 2.54603300 -1.25158300
H 5.83660600 0.92208600 -1.48311100
H 5.93318500 1.69516600 0.10190800
H 3.33058200 0.90124900 -1.30750700
H 3.42405000 1.64509000 0.27974900
H 4.76003900 -0.31917600 1.11035400
H 4.56939800 -1.09945500 -0.44400700
H 2.28994400 -0.29068600 1.43547300
H 2.96081400 -1.91238800 1.30343500
H 1.59877800 -0.38433900 -0.98772500
H -3.10623800 -2.56453700 0.71360200
H -3.89407100 1.38160100 -0.45461600
C -2.21941300 2.68605900 -0.98481300
C -2.07417900 3.22137400 0.43805700
C -1.24476300 2.36893500 1.39620600
C 0.22994200 2.27082300 1.03006700
H 0.78326900 1.69162200 1.77515700
H 0.37807700 1.79751400 0.05594100
H 0.68355300 3.26562100 0.98801900
H -1.33687600 2.79712300 2.39995700
H -1.68360300 1.36328100 1.47652900
H -1.62626300 4.22024600 0.38461600
H -3.07560000 3.36364300 0.86187700
H -2.75720300 3.42741700 -1.58168600
H -1.24283600 2.55913300 -1.46203700
H -3.32411500 1.20464900 -2.10794600

conf_217 12.4 kJ kJ mol^{-1}

C 1.27709200 -2.11640200 -0.46389000
C -0.14337600 -1.67285700 -0.37096100
O -0.62124700 -0.73175600 -0.95690100
O -0.87176500 -2.42788700 0.48517300
C -2.23835000 -2.11000700 0.74269100
C -2.40770300 -0.93552600 1.67841800
H -1.63245200 -0.90181100 2.44097500
H -3.39148800 -0.95631900 2.15348000
O -2.30353700 0.33174500 0.98107300
C -2.71680200 0.43476200 -0.22451700
O -3.28554800 -0.51689400 -0.86093000
C -3.12968300 -1.90983700 -0.46948000
H -2.74487500 -2.41019400 -1.35678900
H -4.14105000 -2.26076700 -0.26227000
C -2.64718200 1.75377100 -0.88420200
H -2.31079500 1.56105700 -1.90874000
C -1.78915100 2.80055300 -0.18214600
C -0.29383200 2.51430400 -0.24598400
C 0.54443800 3.60106900 0.41294800
C 2.03424900 3.29646500 0.36113700
H 2.26621400 2.36000600 0.87903700
H 2.61986700 4.08875000 0.83308700
H 2.38208400 3.19884000 -0.67218400
H 0.34593400 4.56151100 -0.07752800
H 0.22798800 3.72143300 1.45656000
H -0.07392800 1.55242100 0.22998000
H 0.00855500 2.40388100 -1.29520300
H -1.99849200 3.75888100 -0.66719000
H -2.11313700 2.91001400 0.85820100
H -3.69218500 2.08446800 -0.97480900
H -2.59614800 -2.99759600 1.26903700
H 1.45856600 -2.40195200 -1.50613700

C	2.21677000	-0.96819700	-0.07884800
C	3.68314800	-1.34512400	-0.22528900
C	4.62041900	-0.20593600	0.15487000
C	6.08832200	-0.57495000	0.00323100
H	6.34972000	-1.42758700	0.63757300
H	6.32288900	-0.84685300	-1.03055000
H	6.73869700	0.25752300	0.28244500
H	4.39289100	0.66982000	-0.46562500
H	4.42090600	0.09473700	1.19129300
H	3.90385700	-2.22232800	0.39635600
H	3.87840200	-1.64760900	-1.26234400
H	2.01857700	-0.66969000	0.95870600
H	1.98879500	-0.09989500	-0.70556600
H	1.43721600	-2.99492600	0.16302200

conf_0 12.6 kJ mol^{-1}

C	3.73228700	-0.06994500	-1.12974400
C	2.42600500	0.51741000	-0.70613700
O	1.33501900	0.13524100	-1.05289800
O	2.58705000	1.59021400	0.10564300
C	1.45300900	2.29315500	0.60651700
C	0.45145100	2.77562300	-0.42587800
H	0.89824200	2.89534800	-1.41181600
H	0.00287200	3.71916800	-0.11290600
O	-0.66681100	1.86956500	-0.62857700
C	-0.80857100	0.80150500	0.05855300
O	-0.22471200	0.58891600	1.17797100
C	0.72067100	1.55365900	1.70326400
H	1.39628200	0.97339300	2.32847500
H	0.15071400	2.24923300	2.32386700
C	-1.80883000	-0.17865800	-0.38569500
H	-1.77313100	-0.22650000	-1.47594300
C	-3.21695400	0.23556100	0.09040600
C	-4.26309300	-0.77885400	-0.35116000
C	-5.66753100	-0.39962100	0.10141400
C	-6.71413300	-1.41272800	-0.33782300
H	-6.74401800	-1.50438900	-1.42779500
H	-7.71171800	-1.11993200	-0.00254800
H	-6.50331300	-2.40428400	0.07417100
H	-5.68193900	-0.30375300	1.19403900
H	-5.92108000	0.59018000	-0.29747200
H	-4.24254200	-0.87092600	-1.44432500
H	-4.00400500	-1.76754100	0.04795200
H	-3.22079300	0.32067300	1.18295500
H	-3.46072900	1.22336200	-0.31527500
H	-1.53447200	-1.14998400	0.03041500
H	1.90301700	3.17863400	1.06087900
H	4.52952100	0.31312300	-0.48874800
C	3.72141900	-1.59925300	-1.17905800
C	3.88790300	-2.26143700	0.18697900
C	2.76291800	-2.02028300	1.19143200
C	1.42581300	-2.62944300	0.79124500
H	1.05322500	-2.21213500	-0.14801100
H	1.52066100	-3.71135100	0.65959800
H	0.66813500	-2.45989100	1.56237000
H	3.07201700	-2.43612700	2.15629300
H	2.65123700	-0.94083100	1.37217400
H	3.99277800	-3.34163900	0.03405900
H	4.83589200	-1.92634900	0.62489000
H	4.54749800	-1.91506400	-1.82169100
H	2.80414500	-1.93428600	-1.67286900
H	3.91295800	0.33023700	-2.13573400

conf_2 13.8 kJ mol^{-1}

C	3.32323900	-0.49604800	-1.73171700
C	2.07751900	-0.73541000	-0.94299500
O	0.96394600	-0.38039500	-1.24551400
O	2.31838900	-1.46389700	0.17291200
C	1.25347800	-1.80863300	1.05190900
C	0.69507400	-0.64398300	1.84002600
H	1.45627200	0.10262500	2.05667200
H	0.24176800	-0.99194300	2.77089300
O	-0.34025100	0.07610700	1.12292700
C	-1.05685300	-0.51404600	0.24274000
O	-0.99783100	-1.76561400	-0.00816100
C	0.11266700	-2.59193800	0.43119500
H	0.42514300	-3.13861000	-0.45733500
H	-0.31326900	-3.28865000	1.15448900
C	-2.10663500	0.26773700	-0.42663500
H	-1.86144400	1.32556100	-0.32084200
C	-3.48930800	-0.03904000	0.18324400
C	-4.58101500	0.77372700	-0.49920800
C	-5.96164500	0.49623700	0.08207600
C	-7.05417300	1.30666100	-0.59924900
H	-6.87430200	2.38095300	-0.49473200
H	-8.03362700	1.09108800	-0.16620600
H	-7.10705500	1.08034800	-1.66851400
H	-6.18397800	-0.57398400	-0.00924700
H	-5.95294900	0.71780300	1.15636100
H	-4.35334800	1.84305900	-0.40646700
H	-4.58346500	0.54981000	-1.57342100
H	-3.70033300	-1.10868100	0.08110800
H	-3.47338100	0.18946500	1.25515000
H	-2.09285900	-0.00136300	-1.48600900
H	1.74453500	-2.47006500	1.76927300
H	3.05056700	0.16786900	-2.55469700
C	4.52344500	0.04594800	-0.94828300
C	4.39258800	1.49849500	-0.50220700
C	3.34359500	1.76179200	0.57254300
C	3.34238200	3.20821800	1.04635700
H	3.13811900	3.89182500	0.21706500
H	2.58491500	3.38372300	1.81520800
H	4.31336100	3.48249400	1.46853600
H	2.34399400	1.51747800	0.18848600
H	3.53677100	1.09412800	1.42391600
H	5.36679400	1.82620000	-0.12202600
H	4.18312800	2.13129200	-1.37425900
H	5.39475700	-0.04369500	-1.60312800
H	4.71978300	-0.60057500	-0.08662400
H	3.58992800	-1.46607500	-2.16973800

Open Chain

conf_141 -7.9 kJ mol^{-1}

C	1.96121400	-0.54812100	-0.62605600
C	0.77500800	0.15755700	-0.07623600
O	0.37258800	-0.20322100	1.15522100

C	-0.35252600	0.90377700	1.71027100
C	-0.34781800	1.90113000	0.56101200
C	-0.40140800	3.36096700	0.85695700
H	-1.31499200	3.59754200	1.40514300
H	0.46312000	3.63530200	1.46314600
H	-0.38363400	3.93025800	-0.07306300
O	0.78956700	1.52885400	-0.16173000
O	-1.50388900	1.58957500	-0.31766200
C	-1.42171400	0.54697800	-1.08123000
O	-0.43393600	-0.23941400	-1.05363200
C	-2.56180400	0.28478300	-1.98248200
C	-3.84924300	0.02586800	-1.17994300
C	-3.74030800	-1.17048700	-0.24530600
C	-5.03614700	-1.45635100	0.50200500
C	-4.92144000	-2.64512500	1.44383300
H	-5.86436700	-2.83105300	1.96270600
H	-4.65732100	-3.55720800	0.90017400
H	-4.15266400	-2.47782400	2.20534000
H	-5.83472900	-1.63757000	-0.22699400
H	-5.33486000	-0.56289600	1.06408100
H	-2.93651200	-1.00669000	0.48963400
H	-3.44342900	-2.05880300	-0.81751700
H	-4.11364400	0.92874900	-0.62012000
H	-4.65045600	-0.13644400	-1.90655800
H	-2.30254800	-0.57078800	-2.60813500
H	-2.68477200	1.16707900	-2.62013600
H	-1.35457300	0.58813800	2.00175300
H	0.19343000	1.28808300	2.57342700
C	3.22352700	-0.24226800	0.17769000
C	4.43409300	-0.97865700	-0.38106900
C	5.70827100	-0.69556400	0.40387700
C	6.91684300	-1.43179900	-0.15405200
H	6.76461100	-2.51532900	-0.13350200
H	7.11214800	-1.14338600	-1.19153200
H	7.81681800	-1.21297400	0.42539400
H	5.90059700	0.38451800	0.40222800
H	5.55562700	-0.97783500	1.45289700
H	4.23768400	-2.05857500	-0.37900800
H	4.58296800	-0.69550100	-1.43111800
H	3.06587800	-0.53075900	1.22251400
H	3.41173400	0.83699400	0.16689000
H	1.73497800	-1.61815500	-0.60978000
H	2.07496900	-0.24735900	-1.67106600

conf_0 -6.0 kJ kJ mol^{-1}

C	1.59491800	-0.54581200	-0.47641200
C	0.44669900	0.28241000	-0.02307300
O	0.18092300	0.27021000	1.29664800
C	-0.50538400	1.49088400	1.60774900
C	-0.64543000	2.13805200	0.23866000
C	-0.71239100	3.62304700	0.13089700
H	0.20082800	4.05311500	0.54472400
H	-0.80620400	3.91678900	-0.91518500
H	-1.57214300	3.99938300	0.68790200
O	0.42115700	1.58259600	-0.47294700
O	-1.87904600	1.59740100	-0.39432900
C	-1.84107900	0.39049500	-0.86044400
O	-0.82847000	-0.35653000	-0.72966400
C	-3.01604900	-0.08591800	-1.61836900
C	-3.42753200	-1.53535800	-1.33179200
C	-4.16595700	-1.70673200	-0.00753900
C	-3.33982600	-1.44574300	1.24913100
C	-2.21880600	-2.44874100	1.48195600
H	-1.49651200	-2.46359100	0.66139700
H	-1.66896200	-2.22406500	2.39917000
H	-2.62524100	-3.45941400	1.58020800
H	-4.01573300	-1.45587200	2.11033300
H	-2.93810800	-0.41994900	1.23008700
H	-4.55227800	-2.73100600	0.03420800
H	-5.04638700	-1.05308500	-0.00839900
H	-4.08337100	-1.84284800	-2.14996100
H	-2.54776900	-2.18302400	-1.38587200
H	-2.71852300	0.01174400	-2.67260000
H	-3.83511300	0.61736100	-1.45025500
H	-1.46240500	1.27210700	2.08197200
H	0.12081600	2.09261600	2.26868000
C	2.92066300	-0.03168900	0.07968300
C	4.09041400	-0.89383500	-0.37663400
C	5.42752900	-0.40654600	0.16590600
C	6.59498800	-1.26869100	-0.28974600
H	7.54141500	-0.89942500	0.11214500
H	6.47551600	-2.30461800	0.04230400
H	6.67894200	-1.27722300	-1.38089200
H	5.58887300	0.63124200	-0.15116000
H	5.38695500	-0.38893900	1.26202200
H	3.92518200	-1.93147900	-0.05894000
H	4.12725700	-0.91079500	-1.47362300
H	2.87421700	-0.02127300	1.17397900
H	3.07689100	1.00288000	-0.24524800
H	1.40131300	-1.57018000	-0.14470500
H	1.59746900	-0.54481200	-1.56980700

conf_40 -5.2 kJ mol^{-1}

C	1.38355800	-1.46158300	-0.89210700
C	0.59963300	-0.37024600	-0.24979900
O	0.36593700	-0.48762200	1.06711900
C	0.10122800	0.82872500	1.57397600
C	0.16268200	1.67912500	0.31321500
C	0.59189000	3.10330400	0.41261400
H	1.60081500	3.14504000	0.82552200
H	0.58920200	3.56263200	-0.57650400
H	-0.08780400	3.65354000	1.06559700
O	0.98083300	0.91854000	-0.52909200
O	-1.18204600	1.66908600	-0.31342300
C	-1.55375400	0.59103500	-0.92961200
O	-0.84678600	-0.45495300	-0.95266800
C	-2.83516800	0.63617500	-1.66498000
C	-3.67714500	-0.64074400	-1.55372200
C	-4.40879500	-0.77808300	-0.22373300
C	-3.50839000	-0.84985300	1.00338300
C	-4.28685400	-1.08916800	2.28892400
H	-4.82990300	-2.03717300	2.24227400
H	-3.62615600	-1.13000000	3.15853100
H	-5.02023000	-0.29591200	2.46100600
H	-2.95167500	0.09480900	1.10726400
H	-2.76190400	-1.64205800	0.86768100

H	-5.02059800	-1.68569900	-0.26758000
H	-5.11470500	0.05424200	-0.10773500
H	-4.40473200	-0.61601400	-2.36862900
H	-3.03809700	-1.51060400	-1.73521200
H	-2.54541100	0.79988100	-2.71305300
H	-3.38164500	1.52540200	-1.34146500
H	-0.87308900	0.85143900	2.06254200
H	0.88955300	1.10199200	2.27720600
C	2.72388500	-1.72931600	-0.20559500
C	3.73201200	-0.58914800	-0.26970900
C	5.09349300	-0.98781600	0.28518800
C	6.10147200	0.15049000	0.24677900
H	7.06989600	-0.16227900	0.64395900
H	6.26186300	0.50324400	-0.77679200
H	5.76046900	1.00353100	0.84268800
H	4.97383900	-1.34241900	1.31645400
H	5.47780400	-1.84116900	-0.28629000
H	3.84522400	-0.25553400	-1.30969000
H	3.36125800	0.28053100	0.28708200
H	3.14927300	-2.61208400	-0.69405300
H	2.54216400	-2.01577500	0.83556200
H	0.75517700	-2.35550600	-0.85731500
H	1.51743700	-1.19186000	-1.94347400

conf_48 -3.3 kJ mol^{-1}

C	0.13117500	-1.79861600	1.44866000
C	-0.84339400	-1.24028800	0.46948200
O	-1.46868400	-2.11670400	-0.33652900
C	-2.70330300	-1.50973600	-0.74758600
C	-2.64344300	-0.15023900	-0.06366700
C	-3.91052200	0.52165800	0.34290200
H	-4.44682000	-0.11881900	1.04450900
H	-3.68765500	1.47477100	0.82390200
H	-4.53658100	0.69960000	-0.53319700
O	-1.77802700	-0.38289100	1.00747200
O	-1.92355200	0.78963900	-0.96443100
C	-0.63938500	0.65791400	-1.05736700
O	-0.01207200	-0.28368700	-0.49369700
C	0.08535300	1.61861600	-1.91518200
C	1.46042200	2.04093500	-1.38737100
C	1.39809000	3.03129500	-0.23059000
C	0.73402200	2.50685000	1.03646300
C	0.78150500	3.50975600	2.17973600
H	0.28114900	4.44342200	1.90636900
H	0.29409500	3.11933600	3.07632400
H	1.81509900	3.75322600	2.44150100
H	1.22235700	1.57283500	1.34261400
H	-0.31959000	2.25747400	0.83969100
H	0.88487800	3.94444900	-0.55851100
H	2.42350300	3.33436500	0.00786500
H	2.02761400	1.14941700	-1.10319600
H	1.99624700	2.49370200	-2.22541700
H	-0.57324300	2.47134000	-2.09809800
H	0.20257000	1.09517300	-2.87547100
H	-2.74650200	-1.44432800	-1.83506000
H	-3.53318600	-2.10882400	-0.36992100
C	1.05084000	-2.89262200	0.90599200
C	2.07684600	-2.46278000	-0.13919300
C	3.04129200	-1.37936600	0.32662300
C	4.08441500	-1.03543600	-0.72625100
H	4.69358500	-1.90857900	-0.97699700
H	3.61510400	-0.69083200	-1.65445300
H	4.76055000	-0.24912300	-0.38115000
H	2.48653700	-0.47138400	0.59518600
H	3.54077700	-1.71020100	1.24560200
H	2.65287500	-3.35178900	-0.41916000
H	1.57407000	-2.13825500	-1.05788400
H	0.43428700	-3.70426100	0.50850900
H	1.57722900	-3.30605600	1.77245000
H	0.68808600	-0.95983100	1.87319400
H	-0.49182800	-2.20530500	2.25236500

conf_36 -2.0 kJ mol^{-1}

C	0.00410900	-2.70724400	-0.08221200
C	-1.01270300	-1.62657700	-0.22675800
O	-2.18176600	-1.99614200	-0.78745400
C	-3.17874300	-1.06651900	-0.33923000
C	-2.37097100	-0.11353000	0.53213400
C	-3.03478800	0.56015800	1.68396000
H	-3.42277300	-0.19809000	2.36558600
H	-2.31604700	1.18637800	2.21267400
H	-3.86028200	1.17940600	1.32850200
O	-1.27567100	-0.89386800	0.90914900
O	-1.81706600	0.95516500	-0.34324900
C	-0.83140400	0.63750400	-1.11709400
O	-0.39824800	-0.54881100	-1.21201100
C	-0.23897800	1.70071100	-1.95338700
C	0.21618300	2.95517900	-1.18999100
C	1.45312100	2.74004200	-0.32303800
C	1.24132100	1.91580800	0.94458900
C	0.33040400	2.57739400	1.96857000
H	0.22282900	1.95490200	2.86099700
H	0.73886300	3.54028800	2.28833300
H	-0.67065700	2.76862700	1.56945800
H	2.22064600	1.73475100	1.39943900
H	0.86383900	0.91350400	0.70085300
H	1.83738000	3.72507700	-0.03623100
H	2.23852900	2.27924500	-0.93441300
H	0.43620400	3.70695100	-1.95219200
H	-0.61863700	3.35104900	-0.60446600
H	-1.02496700	1.97909900	-2.66801400
H	0.58101300	1.25361200	-2.51895400
H	-3.64966200	-0.57693100	-1.19215400
H	-3.92465200	-1.60404400	0.24829400
C	1.29061300	-2.32401600	0.64985900
C	2.33929400	-1.59372700	-0.18097800
C	3.60418600	-1.30107600	0.61522800
C	4.63241000	-0.51530300	-0.18368900
H	4.93433700	-1.05807300	-1.08464200
H	4.23175900	0.45407000	-0.50011900
H	5.53327500	-0.32343200	0.40377200
H	3.33731600	-0.74472500	1.52291300
H	4.04353200	-2.24508400	0.95858600
H	2.59129100	-2.19849400	-1.06164400
H	1.94145600	-0.65021300	-0.56861500
H	1.73049900	-3.25579000	1.01903300

H	1.04193500	-1.74178300	1.54438000
H	-0.52399000	-3.49585600	0.46364400
H	0.20466700	-3.09800400	-1.08428500

PE(16:0/18:1) + H − 141]$^+$

Figure S6

DIOXOLANE

conf_0 0.0 kJ mol^{-1}

C	-2.38728000	1.61683500	2.42984000
C	-1.42411000	0.53015900	2.76439900
O	-1.45286400	-0.59060300	2.31442000
O	-0.43349600	0.93067700	3.59549100
C	0.45702200	-0.06417900	4.09059300
C	1.52264400	-0.54443600	3.11322300
O	1.04561000	-1.73506100	2.39582000
C	0.72457700	-1.38716100	1.20745400
O	1.09709900	-0.20724300	0.85905600
C	1.85255600	0.38974800	1.95383100
H	2.90098000	0.37806500	1.65645200
H	1.49046500	1.40767600	2.08045800
C	0.03152000	-2.27141500	0.27155600
C	-0.71512600	-3.43068000	0.91775100
C	-1.39057400	-4.31026800	-0.13054500
C	-2.37396700	-3.58279100	-1.04443900
C	-3.49206700	-2.84326800	-0.31895200
C	-4.50279800	-2.24588700	-1.28786500
C	-5.69069700	-1.55062000	-0.61666400
C	-5.32360400	-0.38495900	0.25884100
C	-5.16340500	0.87637700	-0.14576600
C	-5.26280400	1.39860000	-1.54400100
C	-3.99382500	2.13575600	-1.98167900
C	-2.75320300	1.25452800	-2.00036500
C	-1.50062700	1.99563100	-2.44867900
C	-0.21114700	1.19963900	-2.28446800
C	-0.18216700	-0.13319700	-3.02171000
C	1.17124300	-0.82652400	-2.93658400
C	1.17253500	-2.22848100	-3.52593600
H	2.15751600	-2.69843000	-3.45469700
H	0.45304400	-2.87828100	-3.01393600
H	0.89124200	-2.21152000	-4.58293600
H	1.92530300	-0.21082100	-3.44169800
H	1.48768100	-0.86305100	-1.88591800
H	-0.44686700	0.02121300	-4.07583500
H	-0.95681700	-0.79987500	-2.61900100
H	0.63357200	1.81188100	-2.62213300
H	-0.03471400	1.01409200	-1.21545700
H	-1.41278100	2.93078500	-1.87996200
H	-1.61292100	2.29464100	-3.49866700
H	-2.59604600	0.82651600	-1.00028000
H	-2.93623700	0.40077700	-2.66435000
H	-3.82331700	2.99043700	-1.31249300
H	-4.15347500	2.56247600	-2.97863300
H	-5.47302100	0.58607700	-2.24629100
H	-6.10946100	2.09389800	-1.60761400
H	-4.93635100	1.63164800	0.60842200
H	-5.20162200	-0.59572200	1.32028100
H	-6.38674000	-1.22377500	-1.39526800
H	-6.23603600	-2.28598800	-0.01437200
H	-4.88725300	-3.03980100	-1.93978800
H	-3.99217200	-1.52915200	-1.94238800
H	-4.00330400	-3.53825000	0.36147400
H	-3.08176200	-2.04490900	0.31295800
H	-2.81560000	-4.32143300	-1.72288800
H	-1.83954300	-2.88080000	-1.69976800
H	-0.62317800	-4.80172000	-0.74143400
H	-1.91690600	-5.11242300	0.39775900
H	-0.01755800	-4.03263300	1.50755500
H	-1.45324100	-3.02320600	1.61236800
H	0.80164300	-2.62773700	-0.42980300
H	-0.62277200	-1.62466600	-0.32222800
H	2.40434600	-0.87529000	3.66088700
H	0.95829900	0.41664100	4.93170600
H	-0.10146200	-0.93029300	4.45625200
C	-1.79001900	2.58045300	1.38673000
C	-0.98118700	3.72543800	1.98475800
C	-0.19322400	4.50733700	0.93746500
C	1.08467300	3.80068900	0.50312500
C	1.84969500	4.53093600	-0.59480100
C	3.28219800	4.03946500	-0.77440900
C	3.41022900	2.58366700	-1.20352700
C	4.85960200	2.12177400	-1.28570900
C	5.04002000	0.69182200	-1.78130700
C	4.43019900	-0.37203900	-0.87670500
C	4.73977100	-1.78806800	-1.34765200
C	3.95610100	-2.88299900	-0.63390900
C	4.13385200	-2.91861900	0.87908400
C	3.44193500	-4.10803700	1.52851500
H	3.54771900	-4.10003400	2.61691400
H	3.86416400	-5.04879100	1.16432400
H	2.37109500	-4.12746400	1.29774200
H	3.76094200	-1.98280600	1.31952000
H	5.20459000	-2.94279800	1.11520200
H	4.24733200	-3.85735100	-1.04371700
H	2.88868600	-2.77245400	-0.87484900
H	5.81473500	-1.97739700	-1.23598000
H	4.53405800	-1.85377600	-2.42286400
H	3.34103700	-0.23853900	-0.83534500
H	4.80959300	-0.22750500	0.14530400
H	6.11082600	0.48494400	-1.89469400
H	4.60609500	0.59570400	-2.78540600
H	5.32948500	2.22654100	-0.29762800
H	5.40863800	2.80016000	-1.94917000
H	2.92822900	2.44656400	-2.18083000
H	2.85554200	1.94216700	-0.50745500
H	3.82630700	4.18455500	0.16949000
H	3.79094600	4.67117800	-1.51166800
H	1.87295500	5.60076000	-0.35588700
H	1.30060500	4.44333200	-1.54127500
H	0.85027500	2.78247100	0.16712000
H	1.74019900	3.70285300	1.38271400
H	0.07096100	5.49188000	1.33831600
H	-0.82860000	4.69854100	0.06269400
H	-0.28934600	3.34426000	2.74665700

H	-1.66972700	4.39306500	2.51460500
H	-1.18971600	2.01006700	0.66820200
H	-2.61864700	2.99434900	0.80445200
H	-2.66533600	2.16493000	3.33412900

conf_16 1.8 kJ mol^{-1}

C	5.56955100	-1.31851600	-0.06420800
C	4.54530300	-0.94869800	-1.08761000
O	3.49764600	-1.52427200	-1.26804600
O	4.90873300	0.10994300	-1.84216300
C	4.23726700	0.32886700	-3.07634300
C	2.74988500	0.67011300	-3.04262400
O	1.94779300	-0.53082500	-3.29926900
C	1.22390700	-0.77999100	-2.27280800
O	1.21822700	0.12033500	-1.36451300
C	2.15311400	1.17479900	-1.72785500
H	1.57574800	2.09310600	-1.81765700
H	2.87605000	1.25733400	-0.91960000
C	0.42491900	-2.00072700	-2.16872300
C	-1.05516300	-1.75075100	-1.84998700
C	-1.79648100	-3.08002200	-1.77312000
C	-3.29214200	-2.93695100	-1.51669300
C	-3.63732800	-2.30602100	-0.17484400
C	-5.12350400	-2.36563100	0.14681200
C	-5.46738700	-1.71907800	1.48961200
C	-5.25873700	-0.23715800	1.50721700
C	-4.45653200	0.45043100	2.32139700
C	-3.56369400	-0.11369500	3.38875400
C	-2.37533800	0.78778700	3.72308200
C	-1.50125400	1.15308700	2.52564200
C	-0.94153900	-0.03027900	1.74651700
C	0.00058000	-0.93740300	2.52530900
C	0.62706600	-2.00533500	1.63998400
C	1.54076600	-2.98061200	2.36709400
C	2.14258400	-4.02122400	1.43369200
H	2.71663800	-3.54696900	0.62979600
H	2.81247100	-4.70309400	1.96404800
H	1.35917800	-4.63006300	0.96903300
H	2.34050600	-2.42112300	2.86912000
H	0.97817000	-3.47893900	3.16519600
H	1.19907800	-1.51207200	0.84051600
H	-0.17406800	-2.57041300	1.14098100
H	0.79346700	-0.33449500	2.98860100
H	-0.53635700	-1.41945400	3.35113000
H	-0.40546800	0.35254400	0.86838200
H	-1.77279600	-0.62718300	1.35226500
H	-2.08452300	1.77300200	1.83525800
H	-0.67332900	1.78239600	2.87289100
H	-1.76921100	0.30330400	4.49658400
H	-2.74851900	1.71544100	4.17323000
H	-4.14614600	-0.26689900	4.30674600
H	-3.20888800	-1.10851400	3.09667600
H	-4.46322000	1.53723000	2.23516800
H	-5.85839200	0.32381000	0.79063900
H	-6.52287100	-1.91845400	1.71364800
H	-4.89213700	-2.20216100	2.28620300
H	-5.45060300	-3.41191600	0.15645600
H	-5.69362500	-1.87398000	-0.65225100
H	-3.07863600	-2.81896500	0.62136200
H	-3.31372700	-1.25833700	-0.14936400
H	-3.74340500	-3.93443000	-1.56978400
H	-3.75095800	-2.35546200	-2.32701600
H	-1.64180300	-3.62975500	-2.70946500
H	-1.34832600	-3.69189000	-0.97873200
H	-1.13239700	-1.20631700	-0.90591000
H	-1.50100100	-1.11847900	-2.62637300
H	0.88926200	-2.57453600	-1.35430900
H	0.55811200	-2.56541300	-3.09324100
H	2.52967600	1.33063800	-3.88172900
H	4.76983000	1.17392700	-3.51343300
H	4.36864900	-0.53824300	-3.73082200
C	5.01765400	-1.42589600	1.37002000
C	5.46360000	-0.27415000	2.26848000
C	5.04954100	1.11578100	1.79766400
C	3.54732600	1.35781400	1.80494900
C	3.15842800	2.73836600	1.29203300
C	1.65140700	2.93898000	1.22692800
C	1.21231400	4.18234400	0.46317900
C	-0.29982700	4.28179100	0.27535300
C	-0.87426700	3.20487300	-0.63671200
C	-2.39022300	3.23411800	-0.76931400
C	-2.93451300	2.01326700	-1.49665400
C	-4.44732800	2.00892600	-1.65105900
C	-4.96954000	0.76016500	-2.34707600
C	-6.48586000	0.72124400	-2.45729200
H	-6.86361600	1.57716100	-3.02498700
H	-6.95453400	0.75262900	-1.46821400
H	-6.83123400	-0.18708300	-2.95879300
H	-4.61862200	-0.12115000	-1.79655200
H	-4.52521400	0.69195600	-3.34898200
H	-4.76492100	2.90026000	-2.20742400
H	-4.90801700	2.08916200	-0.65797000
H	-2.63142200	1.11087500	-0.94654400
H	-2.46788100	1.94089500	-2.49040700
H	-2.70309900	4.15008500	-1.28562800
H	-2.83831400	3.28317900	0.23146800
H	-0.58881200	2.21397500	-0.26325600
H	-0.42813900	3.30406500	-1.63923100
H	-0.55405100	5.26853500	-0.12786600
H	-0.78947100	4.21821000	1.25546600
H	1.70161700	4.20125900	-0.52319300
H	1.57814500	5.07322000	0.98594800
H	1.24723000	2.98055500	2.24540700
H	1.19696800	2.04589900	0.77977600
H	3.59822700	2.89452600	0.29455000
H	3.61405100	3.51001300	1.92371900
H	3.15879900	1.22281400	2.82245100
H	3.03684500	0.59122700	1.20224800
H	5.44269700	1.30196300	0.78813100
H	5.53189700	1.86257300	2.43810700
H	6.55531600	-0.30815400	2.36232100
H	5.06926500	-0.44789400	3.27663200
H	3.92698300	-1.49888700	1.33423700
H	5.35978700	-2.36507100	1.81095000
H	6.41308700	-0.62895300	-0.12403400

conf_4 5.8 kJ mol^{-1}

C	-2.11340200	0.97868500	-2.93353600
C	-1.03260400	1.57317000	-2.09037000
O	-0.71513500	1.18243100	-0.99168400
O	-0.42172200	2.62740500	-2.66583400
C	0.39783300	3.44685800	-1.83764200
C	1.68909100	2.83197400	-1.30338000
O	1.49577300	2.35261100	0.06911100
C	1.58327200	1.07571800	0.08163500
O	1.99220800	0.53706100	-1.00802400
C	2.22452400	1.57728000	-1.99651700
H	3.29712300	1.60576800	-2.18493100
H	1.67622700	1.30124400	-2.89553000
C	1.31188200	0.27854900	1.27829600
C	0.13962500	0.78697700	2.11793600
C	-0.05988500	-0.06040700	3.37092500
C	-0.26099300	-1.55265800	3.11287100
C	-1.37806200	-1.87247600	2.12881200
C	-1.64150100	-3.36458400	1.98786100
C	-2.78166800	-3.68025600	1.01764900
C	-4.10797300	-3.19824900	1.51741100
C	-4.97216900	-2.38686100	0.90604200
C	-4.83823400	-1.75362400	-0.44262200
C	-5.03912700	-0.23679600	-0.41171900
C	-3.99543300	0.49986000	0.41571800
C	-4.19171300	2.00912700	0.43526900
C	-3.12447300	2.74414800	1.23463900
C	-3.34195200	4.25048800	1.29938300
C	-2.29808300	4.99829500	2.12408800
C	-0.90306400	4.99065300	1.51204200
H	-0.19372600	5.55169500	2.12674500
H	-0.51227900	3.97331400	1.40612400
H	-0.91738900	5.45946700	0.52024700
H	-2.62080500	6.03659400	2.25405300
H	-2.25776000	4.56615000	3.13174500
H	-4.33502000	4.44481400	1.72100700
H	-3.36387500	4.66249600	0.28023000
H	-3.10040500	2.34246100	2.25732300
H	-2.14331500	2.52105400	0.79867700
H	-5.18182400	2.24216500	0.84753100
H	-4.20427500	2.39623700	-0.59433100
H	-4.01885100	0.11804600	1.44380300
H	-2.99251400	0.26672300	0.03421600
H	-6.03874400	-0.01257600	-0.01782300
H	-5.03174600	0.14527300	-1.44118000
H	-3.86442600	-1.98663900	-0.88359500
H	-5.59098600	-2.19124800	-1.11147100
H	-5.89934900	-2.15577500	1.43105900
H	-4.38592500	-3.57519700	2.50199600
H	-2.55304200	-3.25677100	0.03357400
H	-2.83305700	-4.76788800	0.87920200
H	-1.87738600	-3.78720500	2.97286200
H	-0.72818800	-3.87036700	1.65106600
H	-2.30081600	-1.37079300	2.44525800
H	-1.14562400	-1.46225300	1.13534700
H	-0.47705800	-2.03840900	4.07091900
H	0.67717900	-2.00801500	2.76705900
H	0.79134100	0.08069200	4.04801100
H	-0.93639000	0.33503400	3.89587000
H	0.31718200	1.82835700	2.40142400
H	-0.76048100	0.77374500	1.50047800
H	2.24473400	0.30263000	1.86266300
H	1.18321000	-0.75494700	0.95106200
H	2.44372800	3.61398400	-1.21881600
H	0.65954700	4.28813000	-2.47981600
H	-0.17833600	3.82030300	-0.98653900
C	-2.12271300	-0.54298200	-2.83930200
C	-1.06347100	-1.20785100	-3.71338500
C	-0.77447400	-2.64581300	-3.28458100
C	0.28719300	-2.72342000	-2.19316600
C	0.42893800	-4.10990400	-1.57111600
C	1.77815800	-4.35417000	-0.90048300
C	2.18451900	-3.29428800	0.11476600
C	3.50431900	-3.60982100	0.81058100
C	4.04090500	-2.47696600	1.68171300
C	4.51512200	-1.26618700	0.88647700
C	5.04349900	-0.13019500	1.75786100
C	5.13925300	1.21620300	1.04535700
C	5.96396400	1.21018700	-0.23527800
C	6.04204800	2.58237000	-0.88825900
H	6.51139400	3.31283700	-0.22287300
H	5.04248500	2.96912200	-1.12891800
H	6.61940700	2.55972800	-1.81589400
H	5.54656500	0.48432400	-0.94416100
H	6.97624200	0.85495000	-0.00948800
H	5.55234600	1.96232400	1.73455600
H	4.12516300	1.57699200	0.80767700
H	6.02871000	-0.40381200	2.15372100
H	4.40029900	-0.01711500	2.64188400
H	3.69682100	-0.89429400	0.25785700
H	5.29312500	-1.58748100	0.18386600
H	4.87353700	-2.84860800	2.29003300
H	3.26598300	-2.16608800	2.39782300
H	4.25761500	-3.86552300	0.05356600
H	3.37582200	-4.51026100	1.42170000
H	1.39277300	-3.18521400	0.86985000
H	2.26060400	-2.32934900	-0.40075500
H	2.55503500	-4.40649300	-1.67506600
H	1.76904800	-5.33788100	-0.41645400
H	0.28703000	-4.86862600	-2.34986000
H	-0.38307600	-4.26553500	-0.84993400
H	0.06525000	-1.98471000	-1.41070000
H	1.24912400	-2.41862500	-2.63040500
H	-0.43978900	-3.23862600	-4.14232000
H	-1.70437200	-3.11618200	-2.93859400
H	-0.12469900	-0.63441300	-3.67133600
H	-1.38819600	-1.16954100	-4.75858100
H	-1.97799900	-0.82163200	-1.79097000
H	-3.11124100	-0.91403200	-3.12365700
H	-2.02280900	1.33718800	-3.96139800

conf_31 10.7 kJ mol^{-1}

C	5.52410300	-2.09762300	0.50543600
C	4.53600600	-0.99608200	0.72766800
O	4.58274900	0.09938600	0.22025000

O 3.56688400 -1.33192200 1.60567800
C 2.78480900 -0.29307800 2.18270000
C 1.84874500 0.48981400 1.27147600
O 2.49171800 1.73752300 0.83549600
C 2.66830800 1.69417500 -0.42858500
O 2.11295700 0.71551700 -1.03914200
C 1.44438300 -0.13638600 -0.06366400
H 0.37696700 -0.07886500 -0.26871200
H 1.81181800 -1.14872500 -0.21217500
C 3.37263200 2.74845200 -1.16560700
C 2.36476500 3.60587800 -1.95701000
C 1.40370100 4.37004700 -1.05820800
C 0.28085500 5.04910100 -1.82690100
C -0.67008700 5.81611700 -0.91094700
C -2.05428800 6.03729600 -1.51200900
C -2.88879000 4.75065100 -1.59230100
C -3.20440400 4.19189100 -0.24088600
C -2.74488100 3.06124000 0.29814500
C -1.82200100 2.07150000 -0.34991700
C -2.55081900 0.88783900 -0.99642700
C -3.46703000 0.12780100 -0.04794000
C -4.11773700 -1.08728100 -0.69172300
C -5.02175100 -1.85496200 0.26496100
C -5.53815200 -3.17851400 -0.28921900
C -6.42959500 -3.04460100 -1.51820200
C -6.98925800 -4.38052700 -1.98418900
H -7.59052400 -4.85165500 -1.20011900
H -7.62536900 -4.26483400 -2.86567900
H -6.18467600 -5.07623200 -2.24454500
H -7.25466200 -2.35812300 -1.28821900
H -5.86789900 -2.58156500 -2.33786700
H -6.09721800 -3.70012100 0.49735400
H -4.68497900 -3.82800300 -0.53324700
H -4.47016500 -2.04872300 1.19475700
H -5.87183600 -1.22069000 0.54871700
H -3.33834000 -1.76173300 -1.07198200
H -4.68945100 -0.76404800 -1.56944200
H -2.90071600 -0.19017400 0.83827800
H -4.24686200 0.80407600 0.32143400
H -1.81179100 0.19396900 -1.42447000
H -3.13819700 1.26061500 -1.84386800
H -1.20042000 2.56547400 -1.10428000
H -1.13793200 1.68964700 0.42336700
H -3.08676600 2.80482300 1.29856000
H -3.87551300 4.79914800 0.36591600
H -3.83041600 4.97852900 -2.10502600
H -2.38276600 4.00553700 -2.21418800
H -1.96357100 6.47192400 -2.51440900
H -2.59713800 6.77188800 -0.90686400
H -0.78706800 5.26715100 0.03239700
H -0.22012900 6.77969000 -0.64900800
H -0.27043400 4.28015500 -2.38270900
H 0.69629900 5.72448300 -2.58456900
H 0.94430700 3.68924300 -0.32719500
H 1.95969800 5.10899600 -0.46763100
H 2.94494600 4.29713200 -2.57429000
H 1.81215800 2.95920200 -2.64725500
H 4.07377200 2.24732800 -1.83892300
H 3.93475600 3.34721000 -0.44767700
H 0.98840700 0.81151900 1.85755400
H 2.17256600 -0.80737900 2.92369600
H 3.43213600 0.42146900 2.70007700
C 5.92286100 -2.28243100 -0.96171900
C 4.99654100 -3.22498700 -1.72419000
C 3.52726800 -2.83236100 -1.67441700
C 2.61676500 -3.77180400 -2.45592100
C 1.13227400 -3.45802000 -2.29312300
C 0.58516400 -3.73471800 -0.89717700
C -0.88546400 -3.36596300 -0.74646700
C -1.40722200 -3.42570000 0.68587200
C -0.84914700 -2.32577200 1.58157300
C -1.48471700 -2.27565500 2.96532200
C -0.90660300 -1.18958700 3.86810200
C -1.21422200 0.23286900 3.41506400
C -0.47662500 1.30508700 4.20604700
C -0.74416400 2.71427700 3.69771900
H -0.44780600 2.83032300 2.64812900
H -0.20193000 3.46349900 4.27989100
H -1.80903300 2.95748200 3.75619200
H 0.60456000 1.10012600 4.18356100
H -0.75825100 1.23264700 5.26266100
H -2.29515900 0.40913300 3.48363800
H -0.98145600 0.34822100 2.34685600
H 0.18299100 -1.32721700 3.93892600
H -1.28231500 -1.32296800 4.88860400
H -1.35886400 -3.25133000 3.44819000
H -2.56710500 -2.12673000 2.86086500
H -1.00758700 -1.36532500 1.07245500
H 0.23745200 -2.45106600 1.69865700
H -1.18427900 -4.40695500 1.12343500
H -2.49920300 -3.34074900 0.67283900
H -1.48417700 -4.02683500 -1.38363300
H -1.04806600 -2.35178300 -1.14052900
H 1.17776200 -3.19723100 -0.14617800
H 0.71870200 -4.79776600 -0.65937600
H 0.55569900 -4.04261600 -3.01888100
H 0.95311400 -2.40426000 -2.55497300
H 2.80518200 -4.80494000 -2.13672800
H 2.88674100 -3.72795300 -3.51702500
H 3.40692800 -1.80807600 -2.05703700
H 3.20526200 -2.82402200 -0.62496100
H 5.10396700 -4.23917900 -1.31857100
H 5.32797300 -3.27961600 -2.76721600
H 5.95572500 -1.29901800 -1.44257400
H 6.94135700 -2.67741500 -0.99705700
H 5.15032900 -3.02249800 0.95065500

conf_3 15.8 kJ mol^{-1}

C -2.85185600 -3.20444200 -2.72123400
C -2.48070600 -1.87531000 -2.14998900
O -1.36796100 -1.40750600 -2.09444300
O -3.55788400 -1.18108000 -1.71764900
C -3.32625400 -0.09040800 -0.83650300
C -2.76812900 1.18935100 -1.44354500
O -1.31016000 1.23474300 -1.27351400
C -0.75336500 1.10543500 -2.41600700

O	-1.53819500	1.18092000	-3.42847200
C	-2.90439700	1.36737500	-2.95511500
H	-3.21202500	2.36754200	-3.25715500
H	-3.52159300	0.60753100	-3.43067500
C	0.68647700	0.89038900	-2.56174400
C	1.54842800	1.67289800	-1.56839700
C	2.98749300	1.18286700	-1.60251900
C	3.91882300	1.99460500	-0.71164300
C	5.27416000	1.32043200	-0.56092300
C	6.31056600	2.12449900	0.20891600
C	7.51384900	1.25659500	0.60059900
C	7.19896900	0.33389100	1.73866800
C	7.01979900	-0.98804400	1.70824600
C	7.08124000	-1.91199100	0.53024400
C	5.92431000	-2.91619200	0.52526200
C	4.55988100	-2.26159200	0.35883500
C	3.39024000	-3.22103200	0.52614900
C	2.02349300	-2.57569800	0.31041900
C	1.72716800	-1.41059200	1.24863400
C	0.28063300	-0.94029000	1.18317800
C	0.00373200	0.27097300	2.06014700
H	-1.04837700	0.57047600	2.02871700
H	0.24860900	0.06245100	3.10596600
H	0.60244500	1.13596700	1.75429800
H	0.01798300	-0.71986200	0.14021800
H	-0.37352800	-1.76716200	1.48479400
H	2.39194900	-0.56659900	1.02435400
H	1.96143500	-1.70656100	2.27995300
H	1.94014700	-2.23353200	-0.73179200
H	1.24456800	-3.33880000	0.43560300
H	3.42486200	-3.65487200	1.53434700
H	3.50485900	-4.06179100	-0.16896800
H	4.47359500	-1.44814100	1.08885600
H	4.50596400	-1.79188800	-0.63359400
H	5.94119500	-3.47922100	1.46795000
H	6.08210000	-3.65178600	-0.27193700
H	7.09222000	-1.35005700	-0.40978800
H	8.02556800	-2.47087800	0.56653200
H	6.80721000	-1.48103700	2.65792100
H	7.10397800	0.82420400	2.70749200
H	7.86244100	0.70063000	-0.27611000
H	8.34330200	1.90592500	0.90250000
H	5.85871500	2.54314800	1.11778800
H	6.64491400	2.97758500	-0.39265200
H	5.12754800	0.36303400	-0.05184800
H	5.67594200	1.07986800	-1.55468900
H	3.46596400	2.11665800	0.28156900
H	4.03540600	3.00566900	-1.12288900
H	3.00845600	0.13186600	-1.28673300
H	3.36501200	1.20068800	-2.63288900
H	1.50062500	2.74065400	-1.80693100
H	1.14478800	1.54952000	-0.55906900
H	0.95930100	1.07827800	-3.60210400
H	0.79992100	-0.19051900	-2.38541500
H	-3.13824600	2.04369500	-0.87730000
H	-4.30613200	0.12984900	-0.41305600
H	-2.66314700	-0.39921700	-0.02495100
C	-3.69698200	-4.10594000	-1.81326100
C	-2.96569000	-4.65101900	-0.58704900
C	-2.64878000	-3.64040300	0.51407700
C	-3.87565200	-3.03869800	1.18744000
C	-3.54275900	-2.00911500	2.26018400
C	-4.78192300	-1.34208200	2.85468600
C	-4.51725200	0.00774600	3.51810200
C	-4.22585200	1.11728500	2.51404700
C	-4.08071600	2.50412600	3.12715600
C	-3.78683100	3.58926500	2.09318500
C	-2.36282900	3.55538900	1.55073700
C	-2.13215600	4.47498800	0.36007700
C	-0.68360500	4.48786900	-0.11206400
C	-0.49054600	5.17329700	-1.45611300
H	-0.84385700	6.20827400	-1.42930200
H	-1.05251000	4.66458800	-2.25009500
H	0.56142600	5.19576100	-1.75464600
H	-0.31705000	3.45573600	-0.16272300
H	-0.06552900	4.98215400	0.64607300
H	-2.44909100	5.49524700	0.60876600
H	-2.78748100	4.17095700	-0.47283300
H	-2.08807900	2.53111300	1.26440000
H	-1.66629700	3.82198400	2.35562500
H	-3.97293200	4.57680900	2.52944200
H	-4.50275000	3.49335200	1.26282400
H	-3.28619900	2.49309100	3.88444800
H	-5.00548200	2.75019400	3.66083200
H	-5.04475600	1.14710000	1.77893000
H	-3.31374200	0.87104000	1.95587000
H	-3.68279000	-0.07859700	4.22598400
H	-5.39199500	0.29487100	4.11270100
H	-5.24493400	-2.02540200	3.57446700
H	-5.52987000	-1.19728100	2.06199200
H	-2.87905600	-1.24722000	1.83213200
H	-2.95645500	-2.47781400	3.05999800
H	-4.48020100	-3.84297600	1.62575600
H	-4.51859100	-2.56310400	0.43569700
H	-2.00865300	-2.83825300	0.12263700
H	-2.03474400	-4.14002400	1.27286100
H	-2.03498600	-5.12938300	-0.91550500
H	-3.57863300	-5.45172200	-0.15749700
H	-4.60836900	-3.57580500	-1.52212200
H	-4.02200200	-4.95075400	-2.42745700
H	-1.92023400	-3.69375500	-3.01291000

conf_32   28.3 kJ mol^{-1}

C	7.18887800	-1.03783700	-1.86526700
C	5.73263500	-0.93570300	-1.54899100
O	5.05268000	0.06055300	-1.62040900
O	5.20827100	-2.13010900	-1.19151200
C	3.96980100	-2.16277900	-0.49335100
C	2.72114500	-1.68405500	-1.21947400
O	2.47704600	-0.26697400	-0.94356900
C	2.58114500	0.39737100	-2.03068400
O	2.68225000	-0.29786100	-3.10286400
C	2.73145800	-1.70884600	-2.75011300
H	1.85461500	-2.18019800	-3.19106200
H	3.64715100	-2.11787800	-3.17389900
C	2.46886300	1.85528100	-2.05142400

C	1.02101400	2.27301400	-1.72566600
C	0.86608300	3.78796300	-1.71448600
C	-0.56998300	4.21437800	-1.42232100
C	-1.03041600	3.93450800	0.00291400
C	-2.50836600	4.23413100	0.20739100
C	-2.99043100	4.01209200	1.64448600
C	-2.71849700	2.63071500	2.17394000
C	-3.35764600	1.51799400	1.80935000
C	-4.47041600	1.40991800	0.81519300
C	-5.52744500	0.38470900	1.21270900
C	-6.61952800	0.21678500	0.16689200
C	-7.67044000	-0.81534400	0.54969100
C	-8.76037400	-0.98695300	-0.49899900
C	-9.81087900	-2.01993700	-0.11678400
C	-10.90227600	-2.19278900	-1.16464100
C	-11.94643300	-3.22736600	-0.77178400
H	-12.71802000	-3.33229600	-1.53934800
H	-11.49042300	-4.21156600	-0.62244700
H	-12.44404000	-2.95009700	0.16315100
H	-11.38967800	-1.22532400	-1.33939400
H	-10.44274300	-2.47695700	-2.11996200
H	-10.26953400	-1.73485500	0.83965500
H	-9.32225100	-2.98792300	0.05997700
H	-9.24987000	-0.01968100	-0.67497300
H	-8.30238200	-1.27222900	-1.45605000
H	-8.12812600	-0.52877400	1.50572300
H	-7.18160600	-1.78299800	0.72713800
H	-7.10744300	1.18484400	-0.00820200
H	-6.16364500	-0.06790800	-0.79209200
H	-5.97374800	0.67752400	2.17092500
H	-5.04123400	-0.58475800	1.39016600
H	-4.94038900	2.38784200	0.65934400
H	-4.05650400	1.12130800	-0.16299600
H	-3.05541300	0.58191300	2.27877500
H	-1.92586000	2.53625900	2.91402500
H	-2.51344000	4.74864600	2.29943300
H	-4.06604600	4.21997900	1.68598400
H	-3.09337300	3.60099100	-0.47096100
H	-2.71807100	5.27106000	-0.08199100
H	-0.85379300	2.88453700	0.26632100
H	-0.42939900	4.53132000	0.70235300
H	-1.24391500	3.71304100	-2.13040000
H	-0.66686200	5.28641100	-1.62617800
H	1.54217500	4.22115500	-0.96635000
H	1.17744700	4.18780100	-2.68644000
H	0.34421300	1.84259700	-2.47160500
H	0.73961500	1.84959400	-0.75798700
H	2.77991300	2.21227600	-3.03416500
H	3.15748600	2.24184600	-1.29453400
H	1.85804500	-2.19800000	-0.79416800
H	3.83267800	-3.21798600	-0.25298100
H	4.05122700	-1.60705300	0.44431100
C	8.08053300	-1.39926000	-0.66509100
C	8.34006600	-0.25980200	0.32009700
C	7.12205700	0.29095600	1.06010900
C	6.39941400	-0.73217400	1.92763200
C	5.08597600	-0.22520500	2.50981000
C	4.40962400	-1.25283900	3.41153500
C	2.97764900	-0.91310600	3.81476700
C	1.95118200	-1.13295000	2.70797100
C	0.51663200	-0.88687900	3.16512600
C	-0.54750600	-1.35582700	2.17861100
C	-0.56840800	-0.59601900	0.85962900
C	-1.64051300	-1.08817600	-0.10144300
C	-1.68026600	-0.31923000	-1.41429600
C	-2.78654600	-0.78341600	-2.34923000
H	-2.67442700	-1.84241500	-2.60295900
H	-2.79639400	-0.21450600	-3.28294500
H	-3.76771100	-0.66408100	-1.87943300
H	-0.70687200	-0.41424300	-1.92052700
H	-1.80174500	0.74967800	-1.19628400
H	-2.62122500	-1.01254400	0.38405500
H	-1.49392900	-2.15801500	-0.30680500
H	0.41375200	-0.66416000	0.37498600
H	-0.73271900	0.47046400	1.06296200
H	-1.53419300	-1.26796200	2.64869800
H	-0.40773400	-2.42814900	1.98120500
H	0.37780000	0.18135900	3.37706300
H	0.36345300	-1.40568800	4.11863100
H	2.03444800	-2.17401900	2.35774000
H	2.18256500	-0.49084000	1.84835600
H	2.92729600	0.12637600	4.16401600
H	2.69267700	-1.53272800	4.67266400
H	5.02312000	-1.37856400	4.31054300
H	4.41424600	-2.23564600	2.91640900
H	4.41595200	0.07047600	1.68902300
H	5.26362400	0.69511900	3.07989500
H	7.06125500	-1.05028000	2.74315700
H	6.19919200	-1.64556300	1.35087200
H	6.41818800	0.73501800	0.34704300
H	7.45187300	1.12172600	1.69414300
H	8.83028500	0.56137100	-0.21628100
H	9.07195400	-0.61709000	1.05409500
H	7.66235200	-2.27412000	-0.15795800
H	9.04218300	-1.72327500	-1.07308700
H	7.49256100	-0.08744600	-2.30855800

DIOXANE

conf_0 15.8 kJ mol^{-1}

C	0.31803000	0.87872700	2.09577600
C	-0.07227100	-0.03696100	0.98497200
O	-1.07806100	-0.70298000	0.92403000
O	0.82300300	0.01338500	-0.02585700
C	0.59829400	-0.65922500	-1.26042700
H	1.44248800	-0.32115800	-1.86675900
C	-0.66551300	-0.26233200	-1.99242800
O	-1.80910300	-1.09889500	-1.66481600
C	-1.68653700	-2.17736900	-0.98782300
O	-0.57519600	-2.77771900	-0.79162800
C	0.68218100	-2.16442000	-1.18627900
H	1.41638400	-2.50941200	-0.46029300
H	0.93325400	-2.57996300	-2.16451000
C	-2.91096700	-2.79893100	-0.46161800
C	-4.03717200	-2.95118200	-1.49135900
C	-5.34230300	-3.37658000	-0.82009800
C	-5.96672600	-2.30607600	0.07184700

C	-6.52986600	-1.11888800	-0.70140200
C	-7.01304000	0.02800300	0.18240900
C	-5.92057600	0.72665000	0.99200200
C	-4.76724600	1.25492600	0.14716900
C	-3.83849000	2.19704300	0.90051800
C	-2.65329200	2.64995500	0.06075100
C	-1.77540000	3.70493000	0.71816500
C	-0.57135400	4.07813900	-0.13876800
C	0.37367200	5.09724800	0.48973900
C	1.21808800	4.54225800	1.63111800
C	2.19485900	5.56268500	2.19582600
H	2.88406400	5.91649700	1.42243100
H	2.79378200	5.14224500	3.00821800
H	1.66721400	6.43619400	2.59055400
H	1.77524100	3.66793300	1.26568800
H	0.56636500	4.18148600	2.43700700
H	1.04730500	5.48757500	-0.28298500
H	-0.20305300	5.96041200	0.84617900
H	-0.93627100	4.46261000	-1.09915200
H	0.00137800	3.16941900	-0.38005600
H	-2.37246300	4.60488900	0.91046200
H	-1.45336300	3.34768100	1.70424300
H	-3.01498800	3.03448100	-0.90270100
H	-2.04021700	1.76771000	-0.17549900
H	-4.40708600	3.07506000	1.23177000
H	-3.47451200	1.70752300	1.81366500
H	-5.17151000	1.77911100	-0.72999700
H	-4.17766200	0.41654800	-0.25000400
H	-6.37745100	1.56346000	1.53332500
H	-5.52692900	0.05691400	1.76722900
H	-7.78709000	-0.34133000	0.86571300
H	-7.50318100	0.77313700	-0.45548800
H	-7.36446500	-1.46987100	-1.31973500
H	-5.78245900	-0.73349700	-1.40532800
H	-6.77222800	-2.76190200	0.65816200
H	-5.23592500	-1.95904200	0.81425200
H	-6.05489800	-3.64992800	-1.60609000
H	-5.16551800	-4.29054000	-0.24076700
H	-4.17542600	-2.00632700	-2.02369700
H	-3.74078100	-3.69785500	-2.23485300
H	-2.63967100	-3.75332000	-0.00750100
H	-3.22512800	-2.12312600	0.34472000
H	-0.98314800	0.75203500	-1.75395100
H	-0.52803400	-0.35614400	-3.07054600
C	1.73815100	0.72204700	2.65354200
C	1.85692600	-0.34655200	3.73515100
C	1.63424400	-1.78683300	3.28511100
C	2.70164600	-2.32334500	2.33751000
C	2.63780600	-3.83748200	2.18305800
C	3.70442800	-4.40662700	1.24332700
C	3.39453700	-4.18355600	-0.20229100
C	4.09292100	-3.48424400	-1.10011300
C	5.37653600	-2.73987700	-0.90458200
C	5.35969700	-1.36292200	-1.56902200
C	4.46950200	-0.34981500	-0.86137100
C	4.22188400	0.90436100	-1.68859400
C	3.48557600	2.00440000	-0.93210900
C	3.02746000	3.16823100	-1.80507400
C	1.91214800	2.81239500	-2.78308800
C	1.39536700	4.01615600	-3.55662500
H	1.00316100	4.77957300	-2.87768500
H	0.59497600	3.74289700	-4.25002100
H	2.19619300	4.47744300	-4.14208500
H	1.08353900	2.36006600	-2.21846500
H	2.26216700	2.05095900	-3.49111100
H	2.67614100	3.98166600	-1.15947400
H	3.88127500	3.57384700	-2.36296700
H	2.61288700	1.57848900	-0.42194300
H	4.14151600	2.38138700	-0.13825600
H	5.17947100	1.29799400	-2.05269300
H	3.66400100	0.62265600	-2.59273900
H	3.50791200	-0.81826700	-0.60836900
H	4.92221900	-0.07813600	0.10128400
H	5.02891600	-1.47677100	-2.61079800
H	6.38024100	-0.96897000	-1.62115000
H	5.61707300	-2.64038200	0.15846400
H	6.18571200	-3.33393000	-1.34891600
H	3.72288800	-3.47793900	-2.12796300
H	2.49971200	-4.70056300	-0.55556700
H	4.68220400	-3.99198600	1.50603500
H	3.77722800	-5.48849700	1.40793000
H	1.63989700	-4.13892400	1.83291100
H	2.75495400	-4.29268700	3.17289600
H	2.63308900	-1.83721100	1.35564900
H	3.69264900	-2.05323700	2.72616600
H	0.63843600	-1.90178400	2.83518100
H	1.61852500	-2.41997100	4.18009600
H	2.85384500	-0.27161800	4.18526000
H	1.14737700	-0.10791900	4.53663200
H	2.02313000	1.68616600	3.08455800
H	2.44047600	0.53961600	1.83457600
H	-0.43347300	0.77155800	2.88067200

conf_3 17.4 kJ mol^{-1}

C	0.77712400	0.85140600	2.40293500
C	0.23751400	0.09863400	1.23050000
O	-0.76303100	-0.57865100	1.22837800
O	0.96821700	0.31187700	0.11639600
C	0.60775400	-0.27932000	-1.12812900
H	1.38454100	0.09787100	-1.79674100
C	-0.72040600	0.16797100	-1.69628900
O	-1.83216600	-0.67429600	-1.28868600
C	-1.65115200	-1.81912000	-0.74769600
O	-0.53453900	-2.43921400	-0.73219900
C	0.69246200	-1.78690700	-1.16077100
H	1.47233200	-2.17438300	-0.50613100
H	0.87809200	-2.13374700	-2.17908300
C	-2.83669500	-2.50990100	-0.21883400
C	-3.74925000	-3.01828100	-1.35197900
C	-5.01535400	-3.67208800	-0.79946100
C	-5.94612600	-2.72482100	-0.04681900
C	-6.60522100	-1.67226200	-0.93203400
C	-7.37765300	-0.60377400	-0.16056500
C	-6.52986000	0.23626600	0.79531900
C	-5.32563000	0.89621100	0.13754600
C	-4.52451900	1.79053600	1.07329700

C -3.24024000 2.29754900 0.43358000
C -2.40065900 3.19508700 1.32957500
C -1.09242700 3.62172500 0.67928300
C -0.19895700 4.47155200 1.57200100
C 1.17955300 4.71876100 0.97550500
C 2.08690700 5.54418700 1.87410300
H 2.24334300 5.05003800 2.83868500
H 1.65389100 6.52845700 2.07623700
H 3.06853600 5.70134200 1.41895800
H 1.06501100 5.21837300 0.00532600
H 1.65533000 3.75210400 0.76090200
H -0.69151800 5.43017200 1.77651000
H -0.08851500 3.98628200 2.55273300
H -1.30139800 4.16659000 -0.25106800
H -0.53262500 2.72409400 0.37485200
H -2.97850400 4.08456300 1.60879900
H -2.18582500 2.66815900 2.26939500
H -3.48065900 2.83078500 -0.49650700
H -2.63904500 1.42447500 0.14363800
H -5.14176500 2.63942600 1.39215600
H -4.27615300 1.23489500 1.98760400
H -5.65749200 1.48002100 -0.73194200
H -4.65383000 0.12541600 -0.26477000
H -7.16693100 1.01030200 1.23864100
H -6.18752300 -0.37770600 1.63829800
H -8.18904100 -1.07792500 0.40421800
H -7.86173700 0.06291200 -0.88397600
H -7.28712000 -2.17497600 -1.62789100
H -5.85390700 -1.18004900 -1.56095100
H -6.72687400 -3.31771300 0.44241500
H -5.40130900 -2.24151500 0.77381200
H -5.55536200 -4.11707700 -1.64293700
H -4.72840200 -4.50607800 -0.14829200
H -4.00651900 -2.18240400 -2.00932000
H -3.19620200 -3.74529800 -1.95553800
H -2.49863100 -3.33392100 0.41116600
H -3.36822100 -1.78240200 0.40045500
H -0.99410700 1.17043100 -1.37085200
H -0.70064300 0.13264800 -2.78673400
C 2.29782800 0.92600800 2.54187400
C 2.93346300 -0.32186400 3.14232200
C 2.82333200 -1.60362300 2.32287000
C 3.49990700 -2.76288800 3.04619000
C 3.45409300 -4.12015000 2.35376000
C 4.25264400 -4.21416600 1.04684300
C 3.47825300 -3.90254900 -0.19440900
C 3.88832900 -3.22372500 -1.26855200
C 5.20555000 -2.55005000 -1.49608500
C 5.05583300 -1.21813000 -2.22993700
C 4.42288700 -0.12260000 -1.38213900
C 4.00284000 1.09727600 -2.19081200
C 3.54935200 2.27219200 -1.33093800
C 2.87611900 3.39703900 -2.11223600
C 1.44771400 3.07394100 -2.53755900
C 0.76556100 4.21957100 -3.26961800
H 1.30636400 4.48071400 -4.18400000
H 0.72918300 5.11584100 -2.64271500
H -0.26174600 3.97063500 -3.55166500
H 0.87144600 2.82179300 -1.63668500
H 1.44464600 2.18236400 -3.18069600
H 2.85447500 4.30339600 -1.49708200
H 3.47552000 3.64636100 -2.99724200
H 2.85702500 1.91546800 -0.55707300
H 4.42088300 2.66496200 -0.79495700
H 4.83633800 1.42016900 -2.82715900
H 3.20749000 0.80475200 -2.89304700
H 3.55224200 -0.52583800 -0.84551800
H 5.12898800 0.18150700 -0.59840500
H 4.45099900 -1.37930100 -3.13369300
H 6.03534300 -0.87911300 -2.58341300
H 5.73914200 -2.40221600 -0.55119900
H 5.83568400 -3.21750100 -2.09846300
H 3.21271700 -3.18469700 -2.12587900
H 2.49316400 -4.37201600 -0.24060900
H 5.15350700 -3.59494700 1.11374100
H 4.61343300 -5.24577300 0.93886500
H 2.41120800 -4.41187900 2.16927700
H 3.84897800 -4.86144700 3.05528800
H 4.54969100 -2.49791500 3.22944200
H 3.04218800 -2.86282000 4.03815300
H 3.27624100 -1.45400100 1.33490900
H 1.76753300 -1.86310800 2.15431500
H 3.99547100 -0.11333300 3.31672300
H 2.49869800 -0.49843900 4.13512500
H 2.52330100 1.77698200 3.19167900
H 2.74626100 1.15682800 1.57126400
H 0.30698600 0.42793600 3.29372900

conf_5 19.0 kJ mol^{-1}

C 0.38989300 0.61039100 2.23968700
C -0.02546400 -0.10461500 0.99757300
O -0.97814700 -0.83853000 0.88351500
O 0.77142500 0.21249000 -0.04502600
C 0.55292800 -0.33099100 -1.34262900
H 1.37532300 0.10275000 -1.91702200
C -0.73075800 0.10309600 -2.01366600
O -1.85604000 -0.76395200 -1.70743300
C -1.68552000 -1.94048600 -1.23500300
O -0.55807000 -2.53971900 -1.18936200
C 0.68933200 -1.83273700 -1.43651300
H 1.39854800 -2.23445000 -0.71293600
H 1.00241300 -2.12406600 -2.44065300
C -2.87487300 -2.67463200 -0.77527600
C -4.04796000 -2.67479000 -1.76191700
C -5.29673200 -3.28664400 -1.12734000
C -5.93647100 -2.41751200 -0.04670500
C -6.65283500 -1.19176000 -0.60314200
C -7.12218600 -0.20196200 0.45997600
C -6.00420300 0.44772900 1.27624100
C -4.91876300 1.09465300 0.42546200
C -3.96088700 1.97965700 1.21098900
C -2.80486700 2.47701600 0.35570500
C -1.89950500 3.49920000 1.02686700
C -0.72400800 3.89936400 0.14255000
C 0.23336700 4.91327800 0.76036500
C 1.11889100 4.34219600 1.86154400

C	2.11553500	5.35494500	2.40486500
H	2.77667000	5.71970000	1.61208900
H	2.74330200	4.92315000	3.18898600
H	1.60322900	6.22301800	2.83056400
H	1.66163800	3.47393100	1.46326100
H	0.49709300	3.96964800	2.68553400
H	0.87806700	5.32288100	-0.02655500
H	-0.33777500	5.76486700	1.15178600
H	-1.12020800	4.30110700	-0.79871900
H	-0.15146100	2.99951700	-0.12948100
H	-2.48337700	4.39357800	1.27689800
H	-1.54515100	3.10011000	1.98565500
H	-3.20105000	2.90811900	-0.57426000
H	-2.20596200	1.60494700	0.05638000
H	-4.51092200	2.83510900	1.62239500
H	-3.56602000	1.42687800	2.07379400
H	-5.38659700	1.69329100	-0.36837800
H	-4.33786900	0.31722400	-0.09067100
H	-6.45036900	1.20788100	1.92819600
H	-5.54562100	-0.28507900	1.95234400
H	-7.82159200	-0.70160700	1.14079000
H	-7.69659400	0.58904100	-0.03666600
H	-7.51873100	-1.52824300	-1.18522300
H	-6.00534400	-0.66672500	-1.31590300
H	-6.65304900	-3.02248400	0.51951300
H	-5.17947600	-2.10992900	0.68684700
H	-6.02925400	-3.46933300	-1.92117100
H	-5.04270100	-4.27166600	-0.71821200
H	-4.25072500	-1.65140900	-2.08976200
H	-3.76423900	-3.24646000	-2.65132000
H	-2.56396700	-3.68670200	-0.51068700
H	-3.15945700	-2.16298100	0.15389800
H	-1.04786700	1.09594000	-1.69837200
H	-0.61815900	0.08302500	-3.09918100
C	1.88103200	0.57220900	2.58261700
C	2.35347800	-0.74132300	3.19297000
C	2.32342000	-1.95865000	2.27504500
C	2.82941800	-3.20085700	2.99935800
C	2.85731600	-4.49139300	2.18814900
C	3.86387300	-4.50720900	1.02983100
C	3.33086800	-4.02876800	-0.28320400
C	3.92481300	-3.21757600	-1.16181700
C	5.25157700	-2.53335800	-1.04127900
C	5.24601700	-1.14168400	-1.67118900
C	4.40885300	-0.12583000	-0.90500700
C	4.16605200	1.15962600	-1.68437300
C	3.47055100	2.24497300	-0.87055600
C	3.03149800	3.45909600	-1.68240900
C	1.87386400	3.18230200	-2.63647900
C	1.37359000	4.43289900	-3.34389600
H	1.02277700	5.17760100	-2.62260900
H	0.54639200	4.21301300	-4.02470300
H	2.17130100	4.89598600	-3.93215500
H	1.04888500	2.73655300	-2.06231300
H	2.17523700	2.43928000	-3.38587600
H	2.73057900	4.25732900	-0.99390800
H	3.88305400	3.85577500	-2.25000000
H	2.59221100	1.82052000	-0.36821500
H	4.14690500	2.56748100	-0.06992200
H	5.12192700	1.54769500	-2.05863300
H	3.58063700	0.92105700	-2.58392200
H	3.44384000	-0.57485600	-0.63014300
H	4.90240800	0.10612700	0.04807600
H	4.87066400	-1.22268700	-2.70109300
H	6.27313100	-0.77140400	-1.75623600
H	5.56555300	-2.47191300	0.00633000
H	6.00724700	-3.14804900	-1.54779300
H	3.42238900	-3.05717600	-2.11836600
H	2.37765200	-4.47650000	-0.57262300
H	4.76545700	-3.95371900	1.31255300
H	4.19210500	-5.54350700	0.87424000
H	1.85220200	-4.71625400	1.80590300
H	3.10400600	-5.30856800	2.87300000
H	3.84146400	-3.00171700	3.37612400
H	2.20729000	-3.35946500	3.88887300
H	2.93331800	-1.76398200	1.38443500
H	1.29902200	-2.15231500	1.92547400
H	3.38166400	-0.60164300	3.54676900
H	1.75683300	-0.95426500	4.08983500
H	2.06480000	1.37858100	3.29890900
H	2.46913000	0.81525100	1.69260700
H	-0.22199000	0.21716500	3.05435700

Open Chain

conf_0 1.5 kJ mol^{-1}

C	-1.07690300	-0.81034900	-1.85333900
C	0.26250600	-0.21333500	-2.09577900
O	1.19413500	-1.02571300	-2.63649800
C	2.15795600	-0.19101200	-3.29342600
C	1.63579200	1.20571900	-2.99100900
O	0.27344900	0.98588800	-2.77705800
O	2.19351800	1.62210200	-1.67681600
C	1.67483500	1.10277600	-0.61437300
O	0.79955200	0.18843400	-0.66851000
C	2.08683800	1.63007600	0.69953300
C	3.43744500	2.33493600	0.72752200
C	4.62780600	1.39883200	0.54479600
C	4.76348600	0.35276800	1.64651700
C	6.11095100	-0.36452100	1.63456300
C	6.42104100	-1.20451500	0.39697500
C	5.54948800	-2.45291600	0.22721700
C	4.20428100	-2.23369300	-0.39240600
C	3.03235400	-2.71544100	0.02828500
C	2.79135800	-3.53425000	1.26277000
C	1.38976000	-3.35074100	1.84008500
C	1.09153100	-1.91527200	2.24789700
C	-0.32171900	-1.70048400	2.77037300
C	-0.64350600	-0.23942300	3.05216700
C	-2.06806800	-0.00844400	3.53487100
C	-2.35614700	1.44046000	3.90431100
C	-3.81179900	1.67969000	4.27474800
H	-3.99319500	2.72376600	4.54353800
H	-4.47563100	1.42863800	3.44090600
H	-4.10865300	1.06196600	5.12798500
H	-2.08014600	2.09047700	3.06425000
H	-1.70747600	1.73386300	4.73903300

H	-2.77162700	-0.33275600	2.75521100
H	-2.26808100	-0.65016800	4.40251800
H	0.06208200	0.15229200	3.79778300
H	-0.47912000	0.34927300	2.13701300
H	-1.04465600	-2.09522300	2.04367900
H	-0.46810900	-2.28939300	3.68429500
H	1.25280000	-1.26592800	1.37771900
H	1.81830300	-1.59373100	3.00668700
H	1.26381600	-4.01304300	2.70349500
H	0.64924200	-3.67839600	1.09788000
H	3.53746900	-3.29556400	2.02900600
H	2.93903500	-4.59525300	1.02181900
H	2.15794700	-2.53732300	-0.59750700
H	4.22157800	-1.68594000	-1.33689400
H	5.45141500	-2.96350100	1.19069500
H	6.08699900	-3.15545200	-0.42402000
H	6.36795000	-0.58975000	-0.51116300
H	7.46461000	-1.52669800	0.46996600
H	6.90332900	0.38421500	1.75750100
H	6.17263500	-1.01313300	2.51657800
H	3.95613800	-0.38449100	1.57366400
H	4.64663300	0.84948500	2.61838600
H	4.57169700	0.90894800	-0.43557900
H	5.53647000	2.01111900	0.51828600
H	3.44947800	3.12747500	-0.02620800
H	3.51475200	2.83413500	1.69843500
H	1.27726500	2.32494600	0.96934200
H	2.01518700	0.80643400	1.41457500
C	1.90401500	2.31739400	-3.94748600
H	1.46890500	2.06933000	-4.91653800
H	1.45532300	3.23990800	-3.57669900
H	2.97995100	2.46077500	-4.06078900
H	3.15240300	-0.37423200	-2.88622800
H	2.13731000	-0.40032700	-4.36411300
C	-1.00893700	-2.11835300	-1.07042300
C	-2.38944900	-2.60361000	-0.64514700
C	-3.29296600	-3.01858300	-1.80016600
C	-4.65168400	-3.55486800	-1.34914000
C	-5.49265400	-2.56224700	-0.54898600
C	-5.93009600	-1.33283000	-1.34154500
C	-6.44111200	-0.19093100	-0.46498800
C	-5.35200200	0.52418500	0.33370300
C	-4.37415900	1.32245300	-0.52072900
C	-3.27917200	1.98899700	0.29925000
C	-2.32870600	2.85215900	-0.51858700
C	-1.30476700	3.58701600	0.33543700
C	-0.26535000	4.35157200	-0.47364000
C	0.68347100	5.17704300	0.38321500
H	1.43676400	5.68914900	-0.22153900
H	0.13597700	5.94026300	0.94336700
H	1.21145900	4.56075100	1.11993100
H	-0.77959800	5.01057600	-1.18331900
H	0.30387500	3.64457300	-1.09471100
H	-1.82581000	4.28477300	1.00286400
H	-0.80637600	2.86912400	1.00449800
H	-2.90658500	3.58512600	-1.09569100
H	-1.80955600	2.23476100	-1.26561700
H	-3.74086600	2.60646400	1.08039400
H	-2.70524000	1.21931400	0.83465800
H	-4.92721500	2.08724200	-1.08158600
H	-3.91634700	0.67217700	-1.27802800
H	-5.82554800	1.20615400	1.05005900
H	-4.79464500	-0.20016100	0.94266300
H	-7.19657400	-0.58210800	0.22687300
H	-6.95736700	0.54618300	-1.09182400
H	-5.10294000	-0.96612900	-1.96043700
H	-6.71313400	-1.62920000	-2.04876900
H	-4.93818000	-2.25557500	0.34534400
H	-6.38522000	-3.07480500	-0.17294000
H	-4.49075300	-4.45626200	-0.74534800
H	-5.21459300	-3.87785300	-2.23287000
H	-3.44420800	-2.17324200	-2.48267300
H	-2.78338400	-3.79165600	-2.38881100
H	-2.86927900	-1.81496600	-0.05258600
H	-2.26376800	-3.45621100	0.03272200
H	-0.50745000	-2.88049500	-1.67630100
H	-0.39363100	-1.96455200	-0.17992000
H	-1.67680100	-0.06610900	-1.32152100

conf_3 4.4 kJ mol^{-1}

C	-1.19163400	-1.30874800	-0.50252600
C	0.05688400	-1.01440400	-1.25247700
O	0.85023500	-2.07075700	-1.52871300
C	1.66819400	-1.70592800	-2.64841400
C	1.20738800	-0.28345500	-2.93735800
O	-0.07983500	-0.25915900	-2.39994300
O	2.01629400	0.64050100	-2.09252100
C	1.72852900	0.69973900	-0.83677100
O	0.87683700	-0.07437200	-0.30312700
C	2.36523200	1.73480300	-0.00229500
C	3.59706400	2.40953000	-0.58962800
C	4.84302300	1.52973400	-0.60611300
C	5.28642800	1.07177400	0.77990900
C	6.70122200	0.49991600	0.80917200
C	6.94175200	-0.75156600	-0.03211900
C	6.16198600	-1.99154300	0.41529100
C	4.75018200	-2.07634100	-0.07549500
C	3.67672600	-2.47087500	0.61360200
C	3.63091700	-2.87885200	2.05725200
C	2.26671100	-2.63369900	2.69704000
C	1.89883200	-1.15912600	2.78476700
C	0.46115900	-0.91342300	3.21931200
C	0.04904600	0.55170600	3.18468500
C	-1.41230000	0.78102400	3.54361900
C	-1.82002900	2.24809500	3.53143400
C	-3.28890000	2.45933500	3.86540200
H	-3.55725500	3.51901900	3.84689700
H	-3.93563100	1.93951400	3.15127100
H	-3.52490700	2.07573100	4.86278000
H	-1.60190400	2.67665300	2.54441600
H	-1.19577000	2.80033100	4.24470300
H	-2.05063900	0.22080200	2.84476200
H	-1.61460800	0.35681500	4.53552700
H	0.68753600	1.12776700	3.86746600
H	0.23586500	0.95570900	2.17953400
H	-0.21290400	-1.49166000	2.57174900

H	0.30872900	-1.31225500	4.23000200
H	2.05641600	-0.70287900	1.80018300
H	2.58806000	-0.64922600	3.47084400
H	2.24405200	-3.08025000	3.69684700
H	1.50181400	-3.16272400	2.11151800
H	4.40921100	-2.36087600	2.62917800
H	3.86982900	-3.94816100	2.12847000
H	2.72962200	-2.56187900	0.08132400
H	4.62903800	-1.85965700	-1.13929100
H	6.19752400	-2.07721700	1.50653500
H	6.68534700	-2.87880800	0.03365000
H	6.73423400	-0.54962800	-1.09111700
H	8.01057300	-0.98203400	0.01702400
H	7.39999400	1.28026500	0.48324800
H	6.96639300	0.27476100	1.84910700
H	4.58564800	0.32742100	1.17570600
H	5.24926600	1.92965200	1.46392300
H	4.68092600	0.66170500	-1.25617300
H	5.65250200	2.10389000	-1.07126700
H	3.36771600	2.77587700	-1.59455400
H	3.79123600	3.29678800	0.02110900
H	1.55760200	2.46348700	0.16355700
H	2.54831400	1.28536600	0.97847700
C	1.26913500	0.24305300	-4.33062500
H	0.89824700	1.26855100	-4.35486600
H	2.29880100	0.22387200	-4.69183400
H	0.64860600	-0.37994100	-4.97624600
H	2.72273300	-1.76890800	-2.37813700
H	1.44519400	-2.37275900	-3.48275500
C	-2.18371900	-2.14718600	-1.29964200
C	-3.41818200	-2.44298300	-0.45934800
C	-4.52383200	-3.13988800	-1.23928500
C	-5.78938600	-3.38553900	-0.41557500
C	-6.34144100	-2.14373100	0.28313100
C	-6.70552400	-0.99781300	-0.65801100
C	-6.88101100	0.34389600	0.05110200
C	-5.58225300	0.96776900	0.56289000
C	-4.60654300	1.37374400	-0.53620300
C	-3.36866100	2.08426300	-0.00676500
C	-2.39605600	2.50732200	-1.09962700
C	-1.25203900	3.37347100	-0.59054900
C	-0.26628400	3.78823300	-1.67638000
C	0.72590300	4.84868200	-1.22046700
H	1.28232100	4.53798700	-0.32882900
H	1.45395300	5.08824900	-2.00017700
H	0.20653200	5.77470100	-0.95836700
H	-0.82648800	4.17001800	-2.53825200
H	0.26693200	2.90132300	-2.04891200
H	-1.66953200	4.27557900	-0.12572800
H	-0.72274200	2.84937700	0.21999300
H	-2.94423100	3.06570400	-1.86937900
H	-1.99500700	1.61988800	-1.60974000
H	-3.67897500	2.97131800	0.56031100
H	-2.85446700	1.44014000	0.72127800
H	-5.12132800	2.03288900	-1.24776100
H	-4.29784900	0.49422900	-1.11651700
H	-5.83036500	1.85736600	1.15449400
H	-5.08149100	0.28259200	1.26031500
H	-7.57331300	0.21813200	0.89226300
H	-7.36147800	1.05395800	-0.63288900
H	-5.94373400	-0.89048300	-1.43849200
H	-7.62999700	-1.25375700	-1.18807700
H	-5.61431000	-1.79857800	1.02732000
H	-7.22946700	-2.42612300	0.85977700
H	-5.58071700	-4.15154200	0.34107500
H	-6.55685000	-3.81031900	-1.07306800
H	-4.76634100	-2.53848800	-2.12402300
H	-4.15479800	-4.09876100	-1.62241600
H	-3.79598100	-1.49631500	-0.05700900
H	-3.13555300	-3.05146700	0.40990200
H	-2.47700200	-1.60260900	-2.20436400
H	-1.70909200	-3.07934300	-1.62405500
H	-0.88976400	-1.82313700	0.41459500

conf_16   6.6 kJ mol^{-1}

C	1.13031200	-0.38334000	1.62019200
C	-0.21571600	0.23970000	1.69150900
O	-1.17140100	-0.43042600	2.35466200
C	-2.17728300	0.52777700	2.71841500
C	-1.59839200	1.83445800	2.18550000
O	-0.23185400	1.55520800	2.09439200
O	-2.06063600	2.01483100	0.79132100
C	-1.54367800	1.24478800	-0.10875500
O	-0.72151900	0.32630700	0.17639000
C	-1.88134400	1.50342000	-1.52222800
C	-3.23965000	2.14822100	-1.78822800
C	-4.42301700	1.19137400	-1.68715100
C	-4.66478400	0.58959400	-0.30846300
C	-5.98307300	-0.17371400	-0.23623200
C	-6.28022700	-0.84171700	1.10826900
C	-5.69626800	-2.25023600	1.27871000
C	-4.20966700	-2.32002900	1.45080900
C	-3.30759600	-2.76388500	0.57205700
C	-3.57015500	-3.22922000	-0.82628500
C	-2.64040500	-2.56040300	-1.84418800
C	-1.22537300	-3.12371100	-1.84416900
C	-0.24168000	-2.29366900	-2.65663500
C	1.13504600	-2.93674000	-2.76749700
C	2.14660800	-2.13364700	-3.57765700
C	2.54256400	-0.79800500	-2.95852300
C	3.62903000	-0.08600400	-3.75073700
H	3.30139300	0.11074500	-4.77632900
H	3.90186300	0.87077200	-3.29839200
H	4.53555400	-0.69647200	-3.80769500
H	2.88840400	-0.97145800	-1.93015500
H	1.66335300	-0.14554000	-2.87827500
H	3.05267900	-2.73694100	-3.71026900
H	1.75154000	-1.96066800	-4.58760300
H	1.53697300	-3.11235200	-1.76045200
H	1.02032400	-3.92860100	-3.22044500
H	-0.64464200	-2.12959500	-3.66549800
H	-0.15232000	-1.29954000	-2.19774400
H	-1.25301200	-4.14783800	-2.23685900
H	-0.85650700	-3.21310400	-0.81454700
H	-2.61054400	-1.48222000	-1.63690600
H	-3.06468900	-2.66374000	-2.84942000

H	-4.61150200	-3.04177900	-1.09784000
H	-3.42795400	-4.31706100	-0.88501200
H	-2.26648300	-2.78425300	0.89239500
H	-3.84225900	-2.01439300	2.43158700
H	-6.01571100	-2.86499700	0.43193600
H	-6.15479500	-2.70297000	2.16586100
H	-5.94418400	-0.19248200	1.92930200
H	-7.36564700	-0.92231500	1.22378200
H	-6.78966700	0.53046200	-0.47140100
H	-6.00977700	-0.93085400	-1.03031100
H	-4.68178100	1.39229900	0.44178600
H	-3.84629700	-0.09032500	-0.03592700
H	-5.32050400	1.73868700	-1.99663700
H	-4.29915200	0.38166300	-2.41765200
H	-3.37594200	3.00206900	-1.11705500
H	-3.19878200	2.56128600	-2.79971700
H	-1.07351600	2.17238900	-1.85685900
H	-1.74107700	0.56650800	-2.06868800
C	-1.88598700	3.10793600	2.90628100
H	-1.39350100	3.93897800	2.39967900
H	-2.96229200	3.28765600	2.92951200
H	-1.51073800	3.03457000	3.92786200
H	-3.12726300	0.25911400	2.25530200
H	-2.26732000	0.54516000	3.80531400
C	1.11842400	-1.82779300	1.13390900
C	2.53229200	-2.37548000	0.97425800
C	3.30085000	-2.54223600	2.27950700
C	4.73852500	-3.02199500	2.07914400
C	5.61620500	-2.07864100	1.25784500
C	5.84363300	-0.71348700	1.90160700
C	6.46308500	0.31130200	0.95252500
C	5.50040100	0.84755500	-0.10661300
C	4.41454000	1.76318300	0.44813900
C	3.43630300	2.23569000	-0.61775300
C	2.36553600	3.18821600	-0.10503500
C	1.44005600	3.68704400	-1.20669600
C	0.29567800	4.55707900	-0.70337700
C	-0.56539900	5.13262000	-1.81842700
H	-0.99315800	4.34680100	-2.45183600
H	-1.39376100	5.72856600	-1.42595100
H	0.02586100	5.77973600	-2.47242800
H	0.70854500	5.37761300	-0.10443000
H	-0.32769100	3.97390000	-0.01109900
H	2.02582200	4.25352100	-1.94133900
H	1.03980800	2.82461700	-1.76211200
H	2.84275400	4.04862200	0.38106700
H	1.77373400	2.69625900	0.67953700
H	3.99494400	2.72691100	-1.42508300
H	2.95407700	1.36383500	-1.08086300
H	4.88547800	2.63329700	0.92386400
H	3.86178400	1.25182200	1.24753800
H	6.07044600	1.40143500	-0.86184500
H	5.03222400	0.01361500	-0.64649600
H	7.33026100	-0.14145700	0.45723900
H	6.85312200	1.15703300	1.53160000
H	4.89948700	-0.31488400	2.29139400
H	6.49049100	-0.84086600	2.77731200
H	5.18447900	-1.95417300	0.25788600
H	6.58938800	-2.55522000	1.09380400
H	4.71898800	-4.00553600	1.59415000
H	5.19769600	-3.17777100	3.06248300
H	3.31236600	-1.59792500	2.83687100
H	2.76897200	-3.25903400	2.91697900
H	3.08321000	-1.71415700	0.29525200
H	2.47701600	-3.34635300	0.46874500
H	0.54513800	-2.44652700	1.83270600
H	0.60442000	-1.87091900	0.16962800
H	1.74617100	0.24390400	0.96882200

PC(16:0/18:1) + Na − 59]$^+$
Figure S10

conf_0 0.0 kJ mol^{-1}

C	-0.41277000	-3.51448200	-2.65335800
C	-1.12343100	-2.35085100	-2.03918800
O	-1.10052200	-2.06503300	-0.86266700
O	-1.84349100	-1.67514200	-2.94776700
C	-2.62523600	-0.56694900	-2.50167500
C	-2.07389800	0.74164000	-3.08771300
O	-2.49975700	1.88970400	-2.32676700
C	-2.25434200	2.03675800	-1.01840700
O	-1.70422600	1.20135400	-0.33361900
C	-2.68459400	3.38369700	-0.52776800
C	-3.27312600	3.38046300	0.88159600
C	-4.75297200	3.02215000	0.93307600
C	-5.10424200	1.62964500	0.42498100
C	-6.58638000	1.32464600	0.60850300
C	-7.07029600	-0.00208400	0.03347100
C	-6.46984900	-1.25234400	0.68381400
C	-5.12176600	-1.64113600	0.16604500
C	-4.07363400	-2.07300400	0.86957400
C	-3.99729200	-2.21699400	2.36118300
C	-2.56956500	-2.19255400	2.90470900
C	-1.72983000	-3.39185400	2.47119500
C	-0.33542500	-3.44691000	3.08705500
C	0.60087600	-2.33410600	2.63398200
C	2.00820800	-2.44941600	3.20388900
C	2.88949600	-1.25003400	2.88593200
C	4.31146900	-1.41308100	3.39828300
H	4.80113900	-2.27656100	2.93660300
H	4.32667900	-1.56731300	4.48178400
H	4.92017000	-0.53223500	3.17988400
H	2.89813500	-1.07763200	1.80340900
H	2.44187500	-0.35230800	3.33145500
H	2.47615400	-3.36435300	2.81809400
H	1.94547800	-2.57742800	4.29202300
H	0.67404900	-2.35254600	1.53485800
H	0.19066400	-1.35918000	2.94079100
H	0.12483200	-4.41033700	2.83898300
H	-0.41558800	-3.42228500	4.18111600
H	-2.27690800	-4.29985000	2.75075500
H	-1.63957700	-3.42342300	1.37817000
H	-2.60323100	-2.16511000	4.00016200
H	-2.09629300	-1.24029500	2.61601700

H	-4.58867700	-1.43255700	2.84583800
H	-4.46147900	-3.16671800	2.66218100
H	-3.19161800	-2.38083400	0.31234900
H	-5.02566300	-1.61409400	-0.91999600
H	-6.45222000	-1.13394800	1.77297400
H	-7.14565500	-2.09655800	0.49000900
H	-8.15847800	-0.04124100	0.14598200
H	-6.88010900	-0.02932900	-1.04832500
H	-6.82337200	1.35449700	1.68040100
H	-7.16887900	2.13483500	0.15216000
H	-4.85605500	1.54076100	-0.64290400
H	-4.49633400	0.87944100	0.94335200
H	-5.32435500	3.76773100	0.36445300
H	-5.09243900	3.11494700	1.97154700
H	-2.69310500	2.69333700	1.50517900
H	-3.13373000	4.37745600	1.30919000
H	-3.36263700	3.83713800	-1.25415600
H	-1.76046300	3.97721200	-0.54215000
H	-2.55230200	0.92870500	-4.05290600
C	-0.58749000	0.75802500	-3.38653600
O	0.17217500	0.20766000	-2.31487500
P	1.28810300	0.91516500	-1.44672800
O	1.35086100	0.25082800	-0.13217400
O	0.96494100	2.49064200	-1.51130900
C	2.17343000	3.21414200	-1.82945200
C	2.96593100	2.27306300	-2.73132800
O	2.66911200	0.95027300	-2.25239000
H	4.04234000	2.42292200	-2.65523500
H	2.65511300	2.34492600	-3.77729200
H	1.88797200	4.14210400	-2.32266000
H	2.70423400	3.43031700	-0.89921400
H	-0.39912600	0.13552900	-4.26303800
H	-0.28223200	1.78588300	-3.59887000
H	-2.63839300	-0.53589100	-1.41694700
H	-3.63796300	-0.73413200	-2.86946300
C	0.83135000	-3.96110300	-1.89750700
C	1.98800200	-2.96892500	-1.96813900
C	3.31187500	-3.58212600	-1.52397100
C	4.51600900	-2.65724200	-1.68445000
C	4.61893300	-1.55940400	-0.63350500
C	5.79285800	-0.61835300	-0.88366300
C	6.05477800	0.37641800	0.24346700
C	4.92789800	1.37640500	0.46300800
C	5.15525000	2.30462300	1.64946300
C	4.04053300	3.32639100	1.85753000
C	2.66992900	2.69140600	2.04893900
C	1.55661200	3.68399500	2.36024700
C	0.15930700	3.10775300	2.14254300
C	-0.10826900	1.82245900	2.91511700
H	-1.13579500	1.47209700	2.75949400
H	0.58714200	1.03212800	2.61241500
H	0.02103600	1.96349700	3.99276400
H	0.02287200	2.91228100	1.07202800
H	-0.58884500	3.85916800	2.41848500
H	1.65780500	4.04296100	3.39213600
H	1.66994400	4.57098500	1.72298300
H	2.39450900	2.12119500	1.15555400
H	2.73940200	1.94584300	2.85076400
H	4.00836100	4.01495000	0.99941400
H	4.27844000	3.95183300	2.72626500
H	5.25712300	1.70005200	2.56020000
H	6.11144700	2.82723000	1.52690500
H	3.98623100	0.83531800	0.59515800
H	4.80971100	1.98224600	-0.44844400
H	6.22668700	-0.17885300	1.17465000
H	6.98568500	0.91981500	0.04094400
H	5.62355200	-0.07526500	-1.82407000
H	6.69767400	-1.21782000	-1.04242100
H	3.68560200	-0.98956800	-0.59353000
H	4.73674400	-2.02497200	0.35451600
H	5.43312800	-3.25714500	-1.65203200
H	4.48929600	-2.20442800	-2.68533600
H	3.48081500	-4.49238400	-2.11206700
H	3.23554300	-3.90811500	-0.47760300
H	2.09738800	-2.61616900	-3.00325200
H	1.75693600	-2.08757300	-1.36040800
H	0.57205300	-4.16647300	-0.85332000
H	1.15100900	-4.91560200	-2.32927900
H	-0.18842600	-3.27508800	-3.69656600
H	-1.15457900	-4.32392000	-2.68434800
NA	-0.60770600	-0.49944800	0.71084000

conf_53 4.4 kJ mol^{-1}

C	-2.90196200	-3.06421400	-2.70055100
C	-3.11371900	-1.78895300	-1.94489000
O	-2.82596000	-1.61115500	-0.78186800
O	-3.69427700	-0.84713900	-2.70393300
C	-3.92247600	0.43341400	-2.11593100
C	-3.05429800	1.48967700	-2.81535600
O	-2.87215500	2.66104000	-1.99286700
C	-2.29954400	2.61235100	-0.78351700
O	-1.91247100	1.58624700	-0.26717800
C	-2.18769300	3.96375200	-0.15487200
C	-0.74360000	4.29607100	0.28657600
C	-0.55861200	4.38576700	1.79930400
C	-0.66010600	3.06501700	2.55518500
C	0.45452000	2.07488900	2.23760700
C	0.48311100	0.89202900	3.19812200
C	1.49639400	-0.18803600	2.81416800
C	1.64252400	-1.24147700	3.86642500
C	1.41839900	-2.55219900	3.75713200
C	0.94433400	-3.30893100	2.55778600
C	-0.27990100	-4.18303100	2.85795500
C	-1.45038800	-3.43251800	3.48654900
C	-1.92245800	-2.24247700	2.66505100
C	-3.11071800	-1.50093100	3.26461100
C	-3.51484400	-0.27470700	2.45700900
C	-4.70923700	0.48812700	3.01259100
C	-5.04479400	1.72313600	2.18975200
H	-5.91025300	2.25418000	2.59307600
H	-5.27480800	1.45659200	1.15189200
H	-4.20294900	2.42407300	2.17682700
H	-5.57660600	-0.18081500	3.05152500
H	-4.50106200	0.77904900	4.04866100
H	-3.74652400	-0.57904200	1.42558500
H	-2.66658900	0.42803500	2.41277700

H -3.96457900 -2.18439200 3.34164100
H -2.86578600 -1.19771800 4.28981900
H -2.17490400 -2.58551900 1.64975100
H -1.07132200 -1.54691000 2.59685600
H -2.28658400 -4.12490800 3.63587100
H -1.16211700 -3.07434300 4.48175100
H 0.01866800 -4.99432100 3.53125500
H -0.60584000 -4.66462600 1.92764900
H 0.73711100 -2.63021900 1.72436800
H 1.75147400 -3.96241600 2.20591600
H 1.59139200 -3.16236600 4.64372600
H 1.98036600 -0.87235700 4.83448900
H 1.24730200 -0.62050100 1.83925500
H 2.47129500 0.29585200 2.66934600
H 0.70952000 1.25537400 4.20807400
H -0.51823600 0.44530200 3.27962500
H 0.36378900 1.71605500 1.20542300
H 1.42217300 2.59008100 2.28619100
H -0.64313900 3.28303600 3.62965300
H -1.63361700 2.59342300 2.36252800
H -1.29366800 5.09358800 2.20154000
H 0.42440300 4.82675800 2.00146900
H -0.04916400 3.56360300 -0.13461100
H -0.47250700 5.25816100 -0.15672700
H -2.85085200 3.94721300 0.71861100
H -2.58310200 4.70990200 -0.84347800
H -3.61256500 1.90244700 -3.66016700
C -1.76397400 0.98527200 -3.43483700
O -1.01553100 0.14446900 -2.55923500
P 0.43154600 0.43293300 -1.97013700
O 0.54176100 -0.20897800 -0.64791300
O 0.63471000 2.02107100 -2.10239600
C 1.82745600 2.32609000 -2.85775100
C 2.06272800 1.10908000 -3.75266500
O 1.54767100 -0.01559900 -3.02141300
H 3.12034100 0.92635700 -3.93901400
H 1.52846000 1.18448400 -4.70399600
H 1.63870000 3.23825000 -3.42283100
H 2.64531900 2.48355300 -2.15477300
H -2.01737300 0.38355000 -4.30944400
H -1.17219600 1.84839800 -3.74937500
H -3.70374300 0.39168700 -1.05316400
H -4.97796900 0.66436300 -2.26224700
C -1.86998100 -3.98079800 -2.05739200
C -0.46526800 -3.39338700 -2.06084200
C 0.57521200 -4.26897400 -1.37617900
C 1.88349900 -3.52685500 -1.13776100
C 2.96005100 -4.38846600 -0.48806700
C 4.12812600 -3.62127800 0.12929100
C 4.97530300 -2.80494700 -0.85277300
C 4.48267800 -1.38628500 -1.14020400
C 4.64101700 -0.41621300 0.02563600
C 4.04881600 0.94948100 -0.28988300
C 4.28780600 2.02143900 0.76268500
C 3.66283100 3.35828300 0.38798200
C 3.81481600 4.43972200 1.44823800
C 3.24745100 5.78108000 1.00844200
H 2.18769800 5.69583000 0.74391200
H 3.33368800 6.53478700 1.79536600
H 3.77493500 6.16123700 0.12768600
H 4.87598600 4.55337400 1.69969000
H 3.32179700 4.11071400 2.37224300
H 4.11230300 3.71589700 -0.55007400
H 2.59252100 3.21152500 0.17985700
H 3.88185400 1.68660800 1.72584900
H 5.36575400 2.15225700 0.91953300
H 2.96716500 0.82751500 -0.43207700
H 4.46122700 1.29732500 -1.24944400
H 4.15628500 -0.81733300 0.92414300
H 5.70548200 -0.31202400 0.27280400
H 3.43300800 -1.40066000 -1.45018100
H 5.04534600 -0.98850800 -1.99561500
H 5.99963300 -2.73422800 -0.46721800
H 5.05248000 -3.36002800 -1.79627000
H 4.77658400 -4.34866200 0.62951600
H 3.74825700 -2.96855200 0.92621900
H 2.49412400 -4.99605800 0.29888000
H 3.34587900 -5.10587000 -1.22414400
H 2.24783100 -3.12379300 -2.09083700
H 1.67996100 -2.65403700 -0.50469700
H 0.75426000 -5.17501700 -1.96884900
H 0.18111000 -4.61229500 -0.40998600
H -0.15251600 -3.18992700 -3.09383400
H -0.48137100 -2.42362200 -1.55340900
H -2.17470300 -4.19767700 -1.02778500
H -1.87844900 -4.93647700 -2.59147200
H -2.63731400 -2.80989500 -3.73151400
H -3.88345500 -3.55224200 -2.75721000
NA -1.35307000 -0.46515600 0.53475700

DAG(16:0/18:1) + H − 282]$^+$
Figure S11

DIOXOLANE
conf_0 0.0 kJ mol^{-1}
O -1.03449000 -2.06773500 0.89800400
C -1.48179800 -2.07403900 -0.29988200
O -0.71292800 -1.57000300 -1.18962100
C 0.58519200 -1.24310100 -0.57057000
C 0.25333500 -1.38422500 0.91556500
H 0.09897600 -0.43475800 1.42741600
H 0.94728500 -2.02346200 1.45624900
C 1.59391800 -2.23872100 -1.07852200
O 1.16448400 -3.49786400 -0.62143200
H 1.78035400 -4.18065600 -0.89591500
H 2.57436000 -1.95434000 -0.67482900
H 1.63768200 -2.18337100 -2.17289000
H 0.82470900 -0.22403900 -0.86946700
C -2.78693500 -2.65678500 -0.63629600
C -3.91729700 -2.27364900 0.33009200
C -4.37059100 -0.82401000 0.19795800
C -3.29142800 0.20168300 0.51569400
C -3.78365800 1.64280000 0.48179500
C -2.70654800 2.66028600 0.84395700

C	-1.55592800	2.75888800	-0.15201700
C	-0.52304900	3.80172400	0.25590200
C	0.55904100	4.07095900	-0.78595300
C	1.41302700	2.86808800	-1.17954200
C	2.10252700	2.17606900	-0.00914400
C	3.20714500	1.21960600	-0.44195700
C	3.74085500	0.34784800	0.68515200
C	4.90526600	-0.54525400	0.27783500
C	5.34286500	-1.49674600	1.38088200
H	5.64576800	-0.94761600	2.27732200
H	4.53074700	-2.17334200	1.67155200
H	6.19010400	-2.11213600	1.06891300
H	5.74844300	0.08684500	-0.02295900
H	4.63706500	-1.11615900	-0.62269500
H	4.04963500	0.98456700	1.52355200
H	2.92666400	-0.27656400	1.08923500
H	4.03129200	1.80074200	-0.87255600
H	2.85408600	0.58317200	-1.26860200
H	2.52982800	2.92965100	0.66408300
H	1.35891800	1.63928900	0.60010300
H	2.17646900	3.20480800	-1.89076500
H	0.80360300	2.13784200	-1.73198800
H	0.09258700	4.48288400	-1.68870700
H	1.22159700	4.85656500	-0.40384600
H	-0.06325300	3.50253100	1.20684000
H	-1.04120500	4.74475900	0.46509400
H	-1.06361700	1.78188400	-0.26127700
H	-1.95576600	3.00610800	-1.14493100
H	-2.30805900	2.42710400	1.84121000
H	-3.17116800	3.64841600	0.93398500
H	-4.18412200	1.86791200	-0.51492000
H	-4.62352400	1.74275100	1.17868700
H	-2.47063900	0.11243000	-0.21045200
H	-2.86686000	-0.00794500	1.50760900
H	-4.75552100	-0.65009900	-0.81476500
H	-5.21974600	-0.67378000	0.87310600
H	-3.60083100	-2.48615400	1.35638100
H	-4.75284500	-2.94488500	0.11733500
H	-2.62132500	-3.74326500	-0.60705100
H	-3.01385000	-2.39140100	-1.67135700

conf_25 6.4 kJ mol^{-1}

O	-0.49388800	-1.68158900	-1.35716500
C	-1.45994500	-2.18523600	-0.68571000
O	-1.18918600	-2.52085100	0.51816600
C	0.23717000	-2.25299500	0.79470500
C	0.67186700	-1.54087200	-0.48830300
H	1.51457100	-2.00257100	-0.99738200
H	0.82833400	-0.47326700	-0.34517900
C	0.35796800	-1.43638300	2.05437800
O	-0.20209800	-0.17890400	1.79336200
H	-0.06566900	0.40868000	2.54009500
H	1.42765600	-1.38023700	2.30069400
H	-0.15291300	-1.96479500	2.86885200
H	0.69673800	-3.23385300	0.92499100
C	-2.77231200	-2.44623700	-1.29238300
C	-3.97723100	-2.05375100	-0.42148700
C	-4.25770200	-0.55634500	-0.41694000
C	-3.11561200	0.28960200	0.12447800
C	-3.46649900	1.76827200	0.24472300
C	-2.33844600	2.62702400	0.80781000
C	-1.13436300	2.78154400	-0.11366500
C	-0.03022400	3.63332600	0.50095400
C	1.09310200	4.00721300	-0.46350900
C	1.81652700	2.83291600	-1.11721400
C	2.46715300	1.86867500	-0.13397700
C	3.28871600	0.78462500	-0.81981000
C	3.82992700	-0.27062800	0.13388600
C	4.56626200	-1.41143600	-0.55483900
C	5.02631800	-2.49450000	0.40907300
H	5.70207000	-2.08687100	1.16675200
H	4.17833400	-2.94608700	0.93843400
H	5.55645300	-3.29759100	-0.10850400
H	5.42929000	-1.00593500	-1.09482200
H	3.92107100	-1.85145800	-1.32917100
H	4.49570200	0.20396800	0.86551200
H	3.00221400	-0.68430000	0.73319400
H	4.12415900	1.24713100	-1.35868200
H	2.68035700	0.30229600	-1.59978300
H	3.10939500	2.42720800	0.55938900
H	1.69311000	1.40180900	0.49277500
H	2.58716500	3.22845800	-1.78925600
H	1.12122900	2.27760300	-1.76141500
H	0.68105200	4.64740400	-1.25261600
H	1.82626800	4.62264500	0.07152300
H	0.38329800	3.11401100	1.37626000
H	-0.47143700	4.55818500	0.89100700
H	-0.73184800	1.79437800	-0.37343700
H	-1.46204800	3.23675200	-1.05822400
H	-2.01420500	2.20841900	1.77085600
H	-2.73328500	3.62380500	1.03464500
H	-3.76473800	2.15455200	-0.73854400
H	-4.34817500	1.86356400	0.88916800
H	-2.23848500	0.19692100	-0.53194100
H	-2.80616900	-0.08549100	1.10800600
H	-4.51225200	-0.22957600	-1.43308700
H	-5.15511100	-0.38563000	0.18773200
H	-3.82148300	-2.42229500	0.59766400
H	-4.84015100	-2.59341900	-0.81892700
H	-2.78308600	-3.53229200	-1.46420800
H	-2.79208900	-1.96025500	-2.27029300

conf_46 8.7 kJ mol^{-1}

O	2.61719800	-0.11854000	1.44882000
C	2.87599100	-1.11570000	0.69178900
O	2.18017500	-2.16884400	0.89496500
C	1.15973400	-1.89205600	1.92494600
C	1.53467200	-0.47022900	2.35971800
H	1.93429600	-0.39786300	3.36961200
H	0.73686900	0.25107300	2.18915100
C	-0.20082300	-2.00909900	1.29032000
O	-0.28343200	-0.99867700	0.32453100
H	-1.17143700	-0.97046300	-0.04758800
H	-0.94655200	-1.88893500	2.08981200
H	-0.31859100	-3.01111900	0.86015800
H	1.30599800	-2.64274200	2.70097000

C	3.89516600	-1.04197500	-0.36072600
C	3.22849700	-0.93354000	-1.75579500
C	2.19128300	0.17746600	-1.86180200
C	2.71815600	1.58551000	-1.60037600
C	1.60572500	2.62874400	-1.53120300
C	0.75522000	2.53558800	-0.26828500
C	-0.39351900	3.53468900	-0.25192200
C	-1.22834600	3.51172400	1.02614000
C	-1.88500400	2.17149600	1.35450900
C	-2.74906500	1.60553500	0.23538900
C	-3.59150400	0.40995400	0.66724100
C	-4.33007000	-0.26507700	-0.48853700
C	-3.44629100	-1.15781200	-1.35919600
C	-3.15132000	-2.51970200	-0.73519800
C	-2.16091000	-3.35159100	-1.53799100
H	-1.99522600	-4.33294600	-1.08437500
H	-2.52889200	-3.52030100	-2.55403900
H	-1.18960800	-2.85071200	-1.62961400
H	-4.09483000	-3.06779900	-0.63615200
H	-2.79415400	-2.39747300	0.29831800
H	-3.92652000	-1.32240200	-2.32988300
H	-2.50670000	-0.63478900	-1.60204600
H	-4.79017900	0.51305800	-1.10761900
H	-5.15763100	-0.86853200	-0.09856200
H	-2.95882600	-0.32415200	1.19100500
H	-4.31415800	0.74505000	1.41937400
H	-2.11396400	1.33400700	-0.61813800
H	-3.42083200	2.38916400	-0.13797500
H	-1.11836400	1.42776600	1.61548800
H	-2.49843100	2.29526000	2.25518600
H	-2.01298600	4.27281400	0.94391200
H	-0.60109500	3.81839200	1.87266600
H	0.00816400	4.54530500	-0.38971500
H	-1.03865000	3.35186300	-1.12022100
H	1.39772200	2.70692900	0.60822600
H	0.35293600	1.52063200	-0.16384800
H	2.04637200	3.62996700	-1.58854700
H	0.96090100	2.53175400	-2.41451000
H	3.43084200	1.85079900	-2.38970200
H	3.28090600	1.62430200	-0.65816000
H	1.76536600	0.14101800	-2.87037600
H	1.35659300	-0.04792000	-1.18760400
H	2.77225000	-1.89768500	-1.99706600
H	4.04553200	-0.77883800	-2.46675200
H	4.49950400	-1.95186600	-0.30861700
H	4.52630800	-0.17767200	-0.15341500

conf_139 12.7 kJ mol^{-1}

O	-1.41098000	-1.02822500	-1.17171000
C	-2.50569000	-1.66458200	-1.00184100
O	-2.46301500	-2.64117100	-0.17584400
C	-1.13271600	-2.68767300	0.45830200
C	-0.37136300	-1.62036300	-0.33567300
H	0.38808700	-2.01456600	-1.00876300
H	0.02588200	-0.82017300	0.28564600
C	-1.29539700	-2.37689800	1.92339800
O	-1.76625200	-1.05672300	2.00248900
H	-1.87184700	-0.79330200	2.91963100
H	-0.31155500	-2.50230000	2.39760800
H	-1.98900900	-3.09840300	2.37146200
H	-0.75928000	-3.69955000	0.30422400
C	-3.74001100	-1.29510700	-1.70398900
C	-4.78012300	-0.69911500	-0.72583400
C	-4.29610100	0.54376100	0.01082000
C	-3.99449000	1.73902100	-0.88634000
C	-3.54160200	2.97772600	-0.11241200
C	-2.28069500	2.78293600	0.72837600
C	-1.04226300	2.40843700	-0.07609300
C	0.13461200	1.98635300	0.79188400
C	1.36806000	1.58888100	-0.00926100
C	2.44820300	0.91755900	0.82720900
C	3.66407400	0.48218200	0.02217300
C	4.72975900	-0.20356300	0.86810600
C	5.91869700	-0.73466500	0.07355000
C	6.74819100	0.34112000	-0.61814100
C	7.97672200	-0.22440600	-1.31534400
H	7.69534000	-0.95534400	-2.08051300
H	8.55758000	0.56117300	-1.80553000
H	8.63661500	-0.73032800	-0.60349700
H	7.05643500	1.08752500	0.12531600
H	6.13206100	0.87661000	-1.34983400
H	6.57108600	-1.30087000	0.74950700
H	5.56433600	-1.45584200	-0.67658700
H	4.26702900	-1.03466500	1.41745400
H	5.08903000	0.49853800	1.63208600
H	3.34754400	-0.19822100	-0.78258600
H	4.08982900	1.35883800	-0.47902800
H	2.02325000	0.04169400	1.34427100
H	2.76386200	1.59871500	1.62754100
H	1.07367600	0.91842800	-0.83173700
H	1.78553900	2.47435600	-0.50323200
H	-0.18209700	1.14347300	1.42487600
H	0.39577500	2.79237100	1.48852800
H	-1.27572800	1.58467200	-0.76246200
H	-0.75139200	3.25160500	-0.71534800
H	-2.45939000	2.01484600	1.49235400
H	-2.08251800	3.70592000	1.28440600
H	-3.37855100	3.79444600	-0.82474300
H	-4.35874800	3.30481400	0.54127500
H	-4.89360300	1.98922700	-1.46212600
H	-3.22828900	1.47856100	-1.62700100
H	-5.07161600	0.82683000	0.73162700
H	-3.41640800	0.28679100	0.61348300
H	-5.07123000	-1.47605500	-0.01307900
H	-5.66494400	-0.47251700	-1.32812800
H	-4.14209300	-2.20758100	-2.15582800
H	-3.47988600	-0.59038900	-2.49409200

DIOXANE

conf_10 11.8 kJ mol^{-1}

O	0.40256100	-2.52568200	-1.12938600
C	-0.97184900	-2.10680600	-0.87133600
C	-1.06001300	-1.50895300	0.51664300
O	-0.27031400	-0.35648000	0.62730900
H	-0.73320100	0.40611500	0.26174800
C	-0.49158900	-2.51432700	1.48432300

O	0.85113500	-2.90196000	1.04978300
C	1.19619900	-2.83816500	-0.17707400
C	2.59999400	-3.12182400	-0.52695800
C	3.29874600	-1.83812500	-1.02028200
C	3.32349600	-0.72819900	0.02025900
C	4.03898000	0.51615400	-0.48898600
C	4.16121400	1.62456300	0.55326200
C	2.83841200	2.15259600	1.10449100
C	1.88804600	2.69469500	0.04362700
C	0.70933100	3.45530900	0.64064800
C	-0.28197500	3.98147300	-0.39631200
C	-1.15938100	2.91409600	-1.04765800
C	-2.22411800	2.34525700	-0.11577700
C	-3.06411900	1.24330800	-0.74918400
C	-4.06471400	0.60427700	0.20327700
C	-4.80385200	-0.58609700	-0.39347200
C	-5.78155900	-1.23243200	0.57607100
H	-5.27334100	-1.59123800	1.47792100
H	-6.54452000	-0.51734200	0.89660200
H	-6.29659300	-2.08264000	0.12219600
H	-4.07464200	-1.33615800	-0.73812600
H	-5.33517700	-0.26364900	-1.29638000
H	-3.55315200	0.29629700	1.12874600
H	-4.79255400	1.35969200	0.52258000
H	-3.59231700	1.64432500	-1.62226400
H	-2.40192200	0.46460000	-1.16416100
H	-1.76533400	1.97600000	0.81511300
H	-2.88474200	3.15672600	0.21202000
H	-0.52890100	2.10132000	-1.43920900
H	-1.65792400	3.34033000	-1.92549100
H	0.27606100	4.51300900	-1.17574900
H	-0.93407000	4.72950100	0.06975300
H	1.10136100	4.30201100	1.21537400
H	0.19542600	2.82093700	1.37592400
H	2.43759300	3.36627800	-0.62977100
H	1.52767200	1.87060800	-0.58640900
H	3.06252800	2.95162800	1.82088900
H	2.32731300	1.37308400	1.68479700
H	4.77763200	1.26535000	1.38630000
H	4.71501800	2.45800800	0.10587800
H	3.51656700	0.89394500	-1.37679500
H	5.04432500	0.23811900	-0.82725300
H	2.29802800	-0.46453400	0.31052800
H	3.81927900	-1.08970500	0.93104500
H	2.80655400	-1.49491400	-1.93616500
H	4.31715700	-2.12328900	-1.29985300
H	3.09446600	-3.52840200	0.35644900
H	2.59848600	-3.87649400	-1.31987200
H	-0.33953500	-2.08895000	2.47357800
H	-1.08033200	-3.43049200	1.54534100
H	-2.11368700	-1.33501200	0.76528500
H	-1.18403100	-1.38492800	-1.65758000
H	-1.60404100	-2.98797300	-0.99800600

conf_0 13.2kJ mol^{-1}

O	1.02592000	-1.99611700	-0.92950900
C	-0.36748100	-1.58238400	-0.78221200
C	-0.67403600	-1.28186700	0.66981200
O	0.03496800	-0.17066500	1.13826800
H	-0.31567600	0.63844200	0.74845200
C	-0.20475900	-2.45316600	1.49237600
O	1.20391900	-2.71413700	1.20132100
C	1.69850000	-2.46337200	0.04988100
C	3.11352500	-2.82642400	-0.17523100
C	3.89815000	-1.93482100	-1.14095100
C	4.31382700	-0.59397600	-0.54591900
C	3.15472700	0.32809900	-0.19924800
C	3.60139400	1.68645700	0.32879300
C	2.44412000	2.61480300	0.68826300
C	1.66884500	3.13627700	-0.51835300
C	0.51189000	4.06671600	-0.15646400
C	-0.68082900	3.39019000	0.51711000
C	-1.46505200	2.46003600	-0.40287800
C	-2.64845200	1.77801600	0.27420100
C	-3.39621600	0.81024500	-0.63324800
C	-4.47717900	0.01096800	0.07977900
C	-5.18054300	-0.99865100	-0.81718600
C	-6.25767900	-1.79177600	-0.09290000
H	-5.84110800	-2.35166800	0.75114200
H	-7.03305500	-1.12971200	0.30380600
H	-6.74446900	-2.50824800	-0.75908000
H	-4.43644200	-1.68759400	-1.24190100
H	-5.62133100	-0.47280800	-1.67244600
H	-4.04149400	-0.51295500	0.94498900
H	-5.21728600	0.70090600	0.50367000
H	-3.84077600	1.36392400	-1.46895200
H	-2.68292200	0.11428300	-1.10333900
H	-2.31300300	1.25076600	1.18121000
H	-3.34241200	2.54334100	0.64047400
H	-0.79437300	1.69880500	-0.83760200
H	-1.82215400	3.02838600	-1.27040500
H	-1.36487000	4.16103400	0.88901300
H	-0.34954000	2.84521400	1.41268000
H	0.15909400	4.57250000	-1.06308000
H	0.89076700	4.85730700	0.50137000
H	2.36605400	3.67927800	-1.16727200
H	1.29775900	2.30119600	-1.12662400
H	2.83628300	3.47506100	1.24276500
H	1.77464400	2.09232600	1.38398200
H	4.22826800	1.53035800	1.21454700
H	4.24310100	2.17295400	-0.41703300
H	2.51116200	-0.12755700	0.56699100
H	2.52763400	0.46050500	-1.09040300
H	4.92928900	-0.76581100	0.34656500
H	4.96731600	-0.09411100	-1.26912500
H	3.31678000	-1.78629600	-2.05658000
H	4.78908100	-2.49794900	-1.43051300
H	3.59027900	-2.89977000	0.80574100
H	3.06552600	-3.85236700	-0.57152600
H	-0.22297900	-2.23548800	2.55751900
H	-0.74992400	-3.37354300	1.27801900
H	-1.76108700	-1.17855800	0.78469300
H	-0.45429800	-0.70487300	-1.42081800
H	-0.97880800	-2.39215000	-1.18532500

conf_37 15.9 kJ mol^{-1}

O	3.11820100	0.43417200	1.50806000
C	2.16973600	1.38893800	2.08274600
C	1.21577100	1.86332000	1.01634900
O	0.42814700	0.76173400	0.65747800
H	-0.37556900	1.05121900	0.21091200
C	2.04205500	2.36220200	-0.15101500
O	2.97928900	1.32050000	-0.56155500
C	3.40618300	0.44360500	0.26461300
C	4.26333300	-0.63448600	-0.26261100
C	3.42726700	-1.60131600	-1.12946100
C	2.26031500	-2.21497300	-0.37144500
C	1.45104700	-3.19160000	-1.21585700
C	0.23681600	-3.75497200	-0.48198700
C	-0.81905300	-2.70782300	-0.14991900
C	-2.02448900	-3.26815300	0.59530800
C	-3.03506300	-2.19340000	0.98681800
C	-3.83824200	-1.64381200	-0.18768300
C	-4.69918300	-0.43184900	0.16032500
C	-3.92129800	0.82583300	0.54626000
C	-2.98651200	1.33144600	-0.54539700
C	-2.33568100	2.66960000	-0.21827200
C	-1.43562500	3.19607800	-1.32992400
C	-0.75736100	4.51549400	-0.99139400
H	-0.15521200	4.44526700	-0.07666700
H	-0.10607000	4.85993900	-1.79960200
H	-1.49863400	5.29948900	-0.81444100
H	-2.03277900	3.31860000	-2.24013800
H	-0.68609400	2.43280400	-1.59322200
H	-3.11673500	3.41060800	-0.01039000
H	-1.77117400	2.59095200	0.72649200
H	-2.21165500	0.57663200	-0.75204800
H	-3.54497100	1.42954500	-1.48532200
H	-4.63859200	1.61892800	0.78859400
H	-3.34899700	0.65199100	1.46656300
H	-5.37582900	-0.69454700	0.98214300
H	-5.34181300	-0.19449800	-0.69614500
H	-4.48722800	-2.44034000	-0.56965400
H	-3.16922900	-1.39016900	-1.01847200
H	-2.49611200	-1.38010500	1.49167500
H	-3.73358600	-2.59532500	1.72992400
H	-2.51654400	-4.03585900	-0.01567000
H	-1.67092400	-3.77932100	1.49888700
H	-1.14248600	-2.22770800	-1.08309900
H	-0.38066800	-1.91300800	0.46731000
H	-0.21757700	-4.53906000	-1.09781500
H	0.56636500	-4.24938200	0.44150900
H	2.10122700	-4.01411900	-1.53601200
H	1.11818300	-2.68826600	-2.13356400
H	2.63258800	-2.73081100	0.52375100
H	1.59787500	-1.41697700	-0.01468200
H	4.11253100	-2.37631600	-1.48508800
H	3.06958200	-1.06564600	-2.01479500
H	5.05219600	-0.17367300	-0.86440200
H	4.71045500	-1.15529400	0.58544500
H	1.44549900	2.55450000	-1.03981600
H	2.63517800	3.24554000	0.09419400
H	0.62748000	2.69670300	1.41943600
H	1.66239400	0.82523100	2.86234200
H	2.76173300	2.19643300	2.51529500

conf_84 20.0 kJ mol^{-1}

O	-2.61854000	1.68114600	1.09719100
C	-1.16625600	1.81490700	1.10972600
C	-0.67267800	2.15758100	-0.27966200
O	-1.16432000	3.40034800	-0.70894900
H	-0.67988800	4.11968800	-0.29519100
C	-1.22113400	1.12726200	-1.23091900
O	-2.66690100	1.00991400	-1.05537300
C	-3.23940800	1.29737200	0.04636900
C	-4.71410400	1.22103400	0.08995500
C	-5.30511200	-0.06665000	-0.50949700
C	-5.25628200	-1.26514700	0.43574800
C	-3.86675400	-1.66630200	0.91914900
C	-2.92157100	-2.19061000	-0.15706600
C	-1.48350600	-2.28610100	0.33493600
C	-0.48839300	-2.74673700	-0.72391400
C	0.97285100	-2.61916300	-0.29774000
C	1.45592400	-1.17781000	-0.18910200
C	2.90756000	-1.02501700	0.23879900
C	3.32868100	0.43442500	0.36287800
C	4.75822900	0.64400100	0.85299300
C	5.82939200	0.11463500	-0.09239300
C	7.24457900	0.43307700	0.37240900
C	8.31300100	-0.10396100	-0.56788000
H	8.20467500	0.31848900	-1.57206700
H	9.31779900	0.13928500	-0.21316300
H	8.24848700	-1.19300900	-0.65888100
H	7.39478100	0.01816600	1.37695600
H	7.35430500	1.52007200	0.47411100
H	5.73029700	-0.97214000	-0.20471500
H	5.67259100	0.54010100	-1.09369800
H	4.92561000	1.71654100	1.01102300
H	4.87534100	0.17197300	1.83742000
H	3.20729700	0.92888100	-0.61234600
H	2.64011100	0.94003700	1.05697100
H	3.54923800	-1.54128800	-0.48331200
H	3.05975100	-1.53059700	1.20104400
H	0.83033400	-0.63013200	0.53213800
H	1.32371300	-0.68422300	-1.16432700
H	1.60579900	-3.14684100	-1.01938900
H	1.12045300	-3.12710000	0.66367500
H	-0.70629000	-3.78858100	-0.98206100
H	-0.64425400	-2.17764500	-1.65300900
H	-1.43116800	-2.95957500	1.19918800
H	-1.18416500	-1.30177400	0.72627500
H	-3.26565400	-3.17446400	-0.49625300
H	-2.95350800	-1.54876600	-1.04689800
H	-3.97430100	-2.42853400	1.69827100
H	-3.39792800	-0.81659300	1.43976100
H	-5.88620900	-1.04908100	1.30633100
H	-5.72645500	-2.11452800	-0.07191700
H	-4.80880300	-0.28298800	-1.45965200
H	-6.34734800	0.14893200	-0.75642500
H	-5.03129500	1.38177900	1.12225900
H	-5.05291400	2.08494500	-0.49841600
H	-1.09969700	1.42904400	-2.26871800

H	-0.80468800	0.13454200	-1.06371700
H	0.42279300	2.09286500	-0.29600300
H	-0.97671400	2.60272200	1.83673400
H	-0.76413300	0.86775500	1.47631700

DAG(16:0/18:1) + H − 256]⁺

Figure S12

DIOXOLANE

conf_0 0.0 kJ mol^{-1}

O	-0.59338700	-3.04086700	-0.54247600
C	-0.73355500	-1.94128300	0.09911300
O	-0.27070100	-0.89808700	-0.47291400
C	0.16895100	-1.24175300	-1.83279300
C	0.12855200	-2.77139800	-1.78240000
H	1.10755700	-3.23898900	-1.68225300
H	-0.44827800	-3.21820600	-2.58911800
C	-0.81566300	-0.62206400	-2.78907300
O	-2.04649900	-1.25726700	-2.55004500
H	-2.75363300	-0.81054300	-3.02051100
H	-0.44892800	-0.78858900	-3.81171200
H	-0.85749100	0.45810100	-2.60574000
H	1.16864600	-0.81968000	-1.93548800
C	-1.41673800	-1.85838100	1.39345600
C	-0.74994000	-0.89687900	2.38848600
C	0.47268600	-1.49449900	3.07890800
C	1.62838900	-1.88392000	2.16242800
C	2.33827800	-0.72295700	1.47725100
C	3.49306400	-1.18341600	0.59677200
C	4.15645100	-0.04267700	-0.18099600
C	3.32481700	0.45570600	-1.32285000
C	2.69688400	1.63047400	-1.42347700
C	2.69866800	2.74845700	-0.42747700
C	1.36574600	3.48664900	-0.32253900
C	0.23048100	2.65366700	0.25712200
C	-1.05106100	3.46003900	0.43441400
C	-2.21754000	2.68594400	1.03997200
C	-2.82411900	1.63579300	0.11796200
C	-3.99463000	0.88772100	0.74316000
C	-4.63258800	-0.12712800	-0.19481200
H	-5.04714300	0.36851900	-1.07875600
H	-5.45025100	-0.66861300	0.28771900
H	-3.90425400	-0.86559900	-0.55103600
H	-4.75270600	1.61031400	1.06592600
H	-3.65801600	0.39305400	1.66546900
H	-2.05646400	0.91406200	-0.18890100
H	-3.16390900	2.12562100	-0.80529300
H	-3.00526900	3.39301400	1.32435300
H	-1.89274600	2.21068400	1.97626800
H	-1.35678100	3.87666400	-0.53479400
H	-0.82850900	4.32316700	1.07238600
H	0.54203600	2.25341800	1.23277700
H	0.05120300	1.78191700	-0.38486300
H	1.07647900	3.85771100	-1.31470800
H	1.50709600	4.37676200	0.30055100
H	2.99796400	2.38667500	0.56136500
H	3.46854200	3.46961000	-0.73330500
H	2.17113200	1.84597400	-2.35590100
H	3.28109600	-0.21653700	-2.18303400
H	4.40122900	0.77053300	0.50836000
H	5.11157900	-0.39777700	-0.58388100
H	3.13944300	-1.94753500	-0.11277300
H	4.24407100	-1.68339800	1.21836100
H	1.63842600	-0.12831600	0.87971900
H	2.72587900	-0.04268000	2.24614100
H	1.28687900	-2.61209400	1.40802800
H	2.36104200	-2.44342300	2.75487000
H	0.83090100	-0.77035400	3.81881200
H	0.15550300	-2.37511600	3.64962400
H	-1.50256500	-0.64757300	3.14038500
H	-0.50632600	0.03919000	1.87786500
H	-2.42172900	-1.48909700	1.14079700
H	-1.52981400	-2.86967600	1.78998300

conf_1 2.0 kJ mol^{-1}

O	-0.06669800	-2.95528000	1.22801500
C	0.21013700	-2.07919100	0.33531400
O	-0.12940300	-0.87973100	0.60632000
C	-0.62429000	-0.81018700	1.98726500
C	-0.75658900	-2.29642600	2.33301800
H	-1.78145700	-2.66592600	2.32742700
H	-0.23510500	-2.57797900	3.24526100
C	0.41175300	-0.06948900	2.79205800
O	1.57865600	-0.85233100	2.74121000
H	2.30220000	-0.40579000	3.18617000
H	0.02729800	0.04755700	3.81495900
H	0.55492200	0.92656000	2.35669800
H	-1.56971100	-0.26903900	1.93950400
C	0.91139600	-2.40741600	-0.90962600
C	0.36552800	-1.67866100	-2.14663300
C	-0.95416800	-2.24439600	-2.66113000
C	-2.15628300	-2.08051200	-1.73556700
C	-2.58952400	-0.64129000	-1.48423200
C	-3.87662800	-0.54771400	-0.67415200
C	-4.23142700	0.88598800	-0.26768000
C	-3.42029100	1.38989500	0.88555600
C	-2.55783200	2.40980600	0.90692600
C	-2.16935700	3.31655100	-0.21991400
C	-0.66594300	3.59636200	-0.25784100
C	0.15616100	2.35466300	-0.56741700
C	1.66054300	2.54078900	-0.42887200
C	2.43528100	1.24804600	-0.64717400
C	3.94293700	1.37741500	-0.49502300
C	4.67033400	0.04913800	-0.65995500
C	6.18047600	0.16683800	-0.51917500
H	6.67363200	-0.80115700	-0.63946300
H	6.45589400	0.56102900	0.46403800
H	6.59273100	0.84492100	-1.27230500
H	4.42587100	-0.37357900	-1.64382700
H	4.28731000	-0.66358300	0.08504800
H	4.17776100	1.80067200	0.49099200
H	4.32598000	2.09673300	-1.22954600
H	2.20917000	0.85818300	-1.64948300
H	2.07299700	0.49262500	0.06481500

H	1.88804800	2.93627600	0.57087500
H	2.00812600	3.30342900	-1.13639900
H	-0.07981300	2.01051000	-1.58419400
H	-0.16743800	1.54623300	0.09878800
H	-0.35457500	4.00875600	0.71140700
H	-0.45833200	4.37539300	-0.99892000
H	-2.49607700	2.90737100	-1.18124200
H	-2.70185500	4.26811500	-0.09393800
H	-2.10215000	2.66038000	1.86735600
H	-3.61726000	0.88010900	1.83182500
H	-4.13584500	1.54611900	-1.13476100
H	-5.28744400	0.91927000	0.02482700
H	-3.79619000	-1.16957100	0.23038100
H	-4.69914500	-0.97738600	-1.25621000
H	-1.79967200	-0.07396100	-0.97988000
H	-2.74235000	-0.14394000	-2.45056700
H	-1.97585000	-2.59104200	-0.77465200
H	-2.99735300	-2.63082800	-2.17253100
H	-1.17743200	-1.75619000	-3.61605700
H	-0.81757700	-3.30727900	-2.89287900
H	1.12771200	-1.77467000	-2.92379200
H	0.28329200	-0.61055300	-1.92770600
H	1.94804300	-2.08702600	-0.72491200
H	0.91791600	-3.49325200	-1.02594700

conf_33 5.8 kJ mol^{-1}

O	0.88785600	-1.83802100	-1.31091100
C	1.66312900	-1.99828500	-0.30575400
O	1.34475600	-1.36447000	0.75534300
C	0.03789200	-0.70608700	0.55527300
C	-0.16339600	-0.89054600	-0.94846500
H	0.01185900	0.01315600	-1.53097100
H	-1.11276600	-1.35422800	-1.20451300
C	-0.96397600	-1.42596700	1.41517800
O	-1.05590400	-2.73852000	0.91848900
H	-1.81318200	-3.18546800	1.30405400
H	-1.91396400	-0.88419600	1.33897100
H	-0.62846000	-1.39944100	2.45925200
H	0.17303700	0.33678900	0.84987600
C	2.83596200	-2.88074000	-0.34419000
C	4.09479400	-2.27427700	0.29943300
C	4.83014900	-1.29267000	-0.60877500
C	4.03915900	-0.06248300	-1.04177600
C	3.67096200	0.91055300	0.07229800
C	2.90050000	2.11511700	-0.45475100
C	2.43412500	3.09859400	0.62460100
C	1.36862000	2.54671300	1.52189600
C	0.06000500	2.82200300	1.46250900
C	-0.61609600	3.74651300	0.49584600
C	-1.30477300	3.03829400	-0.67686400
C	-2.46807600	2.13871100	-0.28025900
C	-3.17794600	1.52263000	-1.48089100
C	-4.41990200	0.70380400	-1.14024200
C	-4.14602600	-0.50141300	-0.25149500
C	-5.34436300	-1.41537300	-0.04020500
C	-5.01234100	-2.61916700	0.82962700
H	-4.23440400	-3.23555600	0.36268000
H	-5.88282200	-3.26083900	0.98448900
H	-4.65872400	-2.30629700	1.81919800
H	-5.71781300	-1.75483200	-1.01326400
H	-6.15958400	-0.84232200	0.41627000
H	-3.79530000	-0.15843900	0.73150100
H	-3.32837200	-1.09465900	-0.69089900
H	-4.88429800	0.36093400	-2.07206000
H	-5.16029000	1.35064500	-0.65322800
H	-2.47267700	0.89302300	-2.04401300
H	-3.46137300	2.32791800	-2.16830500
H	-3.19139600	2.72357200	0.30330500
H	-2.11461400	1.35112400	0.39872200
H	-0.55450500	2.45862500	-1.23369600
H	-1.67149700	3.79954200	-1.37494700
H	-1.36660300	4.33451800	1.03733800
H	0.10373200	4.46437300	0.09440900
H	-0.58503700	2.35042600	2.20440500
H	1.70433300	1.86960800	2.30756600
H	3.29823200	3.39251300	1.23251100
H	2.07891900	4.00855900	0.13499700
H	2.02248500	1.77475500	-1.02276600
H	3.53162000	2.65086900	-1.17302900
H	3.08925100	0.40603000	0.85199400
H	4.58866500	1.25653900	0.56388400
H	3.13064900	-0.36745100	-1.58525900
H	4.62919400	0.47475400	-1.79281600
H	5.73966400	-0.97108600	-0.08963600
H	5.16864100	-1.83077900	-1.50186900
H	4.75971900	-3.10742100	0.53870800
H	3.82723900	-1.81327600	1.25473500
H	2.52525100	-3.77136500	0.21994100
H	3.00112600	-3.18805900	-1.37887500

conf_4 6.9 kJ mol^{-1}

O	0.06853700	0.79206300	-0.37245400
C	0.40337700	1.86703300	0.22756300
O	0.26939200	2.94930800	-0.44529000
C	-0.33024600	2.63796500	-1.75446500
C	-0.37588100	1.10534400	-1.72206900
H	0.32442500	0.62232600	-2.40186000
H	-1.37632600	0.68915500	-1.82179900
C	-1.67713500	3.30837300	-1.82550100
O	-2.47280800	2.71267700	-0.83416700
H	-3.33021000	3.14207700	-0.79091900
H	-2.07570900	3.14974200	-2.83772800
H	-1.54952700	4.38635900	-1.67069600
H	0.35372500	3.03718200	-2.50309100
C	1.00902700	1.86053600	1.56332000
C	0.65850000	0.66796900	2.45474700
C	-0.68062600	0.81449400	3.17279100
C	-1.91312300	0.82806500	2.27475100
C	-2.18603900	-0.48891600	1.55931900
C	-3.46255000	-0.45916400	0.72903300
C	-3.63919200	-1.71083700	-0.13608300
C	-2.70921600	-1.74446700	-1.30996700
C	-1.67783700	-2.56884700	-1.50863300
C	-1.21323300	-3.67323000	-0.60803900
C	0.30064000	-3.87428800	-0.62465500
C	1.08777900	-2.69088700	-0.07929200

C	2.59305100	-2.92968400	-0.08636400
C	3.42622200	-1.76262100	0.43475100
C	3.39981300	-0.53042900	-0.46087100
C	4.26933700	0.61335300	0.04355400
C	4.17623800	1.86334300	-0.81949600
H	4.80977500	2.66955600	-0.44135800
H	3.14914100	2.25087900	-0.86578400
H	4.48611900	1.65396300	-1.84775700
H	5.31247400	0.28118500	0.08775300
H	4.00058100	0.84811500	1.08368600
H	2.36572300	-0.17857200	-0.57040300
H	3.72726100	-0.81141400	-1.47081700
H	4.46647100	-2.08834200	0.54982000
H	3.08446000	-1.49032600	1.44337800
H	2.91588900	-3.17489300	-1.10695900
H	2.80858000	-3.81899300	0.51701000
H	0.75755300	-2.48304300	0.94886900
H	0.83989000	-1.79711500	-0.66499900
H	0.62603000	-4.08168000	-1.65264500
H	0.54703800	-4.77018700	-0.04395000
H	-1.55171500	-3.50198900	0.41915700
H	-1.69033600	-4.60726200	-0.93359600
H	-1.13295100	-2.48122400	-2.45042400
H	-2.94505000	-1.02991800	-2.10137600
H	-3.51806400	-2.60432600	0.48365000
H	-4.66762200	-1.74003200	-0.51415400
H	-3.45725400	0.42899200	0.08303000
H	-4.32652800	-0.35056000	1.39444800
H	-1.34552000	-0.75931200	0.91089200
H	-2.26107500	-1.29119600	2.30504800
H	-1.85039400	1.64253900	1.53641100
H	-2.78192900	1.08346400	2.89246600
H	-0.76978000	-0.01087000	3.88759500
H	-0.65573300	1.73097600	3.77472300
H	1.45578800	0.58784300	3.19803100
H	0.69777600	-0.25277700	1.86576400
H	2.08898600	1.86397200	1.34440400
H	0.79363600	2.82469200	2.03310200

DIOXANE

conf_1 13.4 kJ mol^{-1}

O	1.51111800	-2.19239900	0.84173800
C	0.15389700	-1.99667400	1.35908900
C	-0.08940800	-0.52738600	1.58394900
O	-0.08883000	0.06784700	0.32504000
H	-0.29939200	1.01905700	0.39690500
C	1.03333500	0.00364200	2.44825100
O	2.31452500	-0.33056900	1.83423500
C	2.44560800	-1.35882500	1.08599500
C	3.79039300	-1.64288700	0.54592500
C	4.54356000	-0.44022800	-0.04488000
C	4.22318800	-0.18558500	-1.51426700
C	2.79440200	0.24198800	-1.82900600
C	2.38754200	1.59583000	-1.25856400
C	1.08229700	2.09660900	-1.86578300
C	0.51664900	3.36170400	-1.21120600
C	0.10109000	3.18967700	0.21982000
C	-1.16086800	3.12562200	0.66793300
C	-2.40661000	3.22544100	-0.16455200
C	-3.65281800	2.66535900	0.51950100
C	-3.58407600	1.18066500	0.86120600
C	-3.38824700	0.25929900	-0.33539700
C	-3.40849000	-1.21589500	0.04424200
C	-2.91902600	-2.13656300	-1.06378200
C	-2.94094700	-3.61249100	-0.68804700
C	-2.38419600	-4.51556100	-1.77838000
H	-2.41480700	-5.56803500	-1.48531600
H	-2.95945900	-4.41309400	-2.70331600
H	-1.34390500	-4.26360900	-2.01227000
H	-3.96962200	-3.90873300	-0.45149800
H	-2.37459000	-3.76488500	0.24308100
H	-3.53167600	-1.98480300	-1.96139000
H	-1.89922000	-1.84116600	-1.35185400
H	-4.42260100	-1.50541800	0.34653700
H	-2.78827000	-1.36909800	0.94082300
H	-4.17097900	0.45467800	-1.07912800
H	-2.43590300	0.48199900	-0.83010800
H	-2.77026400	1.01175900	1.58334800
H	-4.50261600	0.89814600	1.38880000
H	-3.84083100	3.23625600	1.43663600
H	-4.51650100	2.84424500	-0.13016800
H	-2.25729700	2.74352800	-1.13589800
H	-2.58216900	4.28653500	-0.38645800
H	-1.31493400	3.07282000	1.74740800
H	0.90233100	3.17943800	0.95930600
H	1.27399300	4.15428900	-1.25510900
H	-0.33162500	3.71721700	-1.80133100
H	0.33085000	1.29904200	-1.82583000
H	1.24720800	2.30117700	-2.92957500
H	2.29462800	1.53151300	-0.16781200
H	3.18061300	2.32993200	-1.45203700
H	2.07219400	-0.51930300	-1.49943900
H	2.68870600	0.27524800	-2.91953900
H	4.91135400	0.58658800	-1.87619900
H	4.46380700	-1.09090700	-2.08436400
H	5.61000000	-0.65775800	0.05107200
H	4.35860100	0.44778500	0.56642600
H	3.69880900	-2.46419400	-0.16775300
H	4.34627300	-2.02940200	1.41256300
H	1.03928300	1.09055600	2.50661300
H	1.04322900	-0.42217400	3.45375600
H	-1.03974000	-0.40856500	2.12216300
H	-0.49201100	-2.39875200	0.58159200
H	0.07837800	-2.59338000	2.26898700

conf_10 14.6 kJ mol^{-1}

O	1.27903200	-2.68086300	-0.53408800
C	-0.10949600	-2.21401300	-0.50232800
C	-0.19717300	-0.91684400	0.25570300
O	0.45686300	0.04351900	-0.51466000
H	0.43847600	0.90781300	-0.06387900
C	0.48138300	-1.11013200	1.59354000
O	1.83022300	-1.63111600	1.38680700
C	2.11841000	-2.34477700	0.36632200
C	3.48809200	-2.88978800	0.27586000
C	4.62930100	-1.90207800	0.56836600

C 5.08906400 -1.12635300 -0.66198700
C 4.07369200 -0.16346900 -1.26617400
C 3.69080300 1.00567800 -0.36717400
C 2.87324800 2.05373500 -1.11165100
C 2.34191400 3.19126100 -0.23314900
C 1.45534700 2.75575700 0.89487300
C 0.14059400 2.98802000 1.00900100
C -0.73695300 3.70853100 0.02597900
C -2.21179700 3.32786600 0.14326300
C -2.45745800 1.83664700 -0.03119100
C -3.92345800 1.43205200 -0.04678500
C -4.11475500 -0.07641800 -0.13032900
C -5.57021500 -0.51547100 -0.19785600
C -5.74286900 -2.02685900 -0.26852600
C -7.20024300 -2.45771000 -0.34004900
H -7.29620500 -3.54542100 -0.38962000
H -7.75620200 -2.11473100 0.53799800
H -7.69107700 -2.04049300 -1.22467700
H -5.26752700 -2.48510800 0.60928100
H -5.20318400 -2.41013100 -1.14473400
H -6.10701000 -0.12831500 0.67781600
H -6.04574800 -0.05413400 -1.07280600
H -3.64400000 -0.54847800 0.74671100
H -3.58291100 -0.45999700 -1.01393800
H -4.42115800 1.81366300 0.85357600
H -4.42361700 1.91111000 -0.89738000
H -1.97597600 1.49457300 -0.95831600
H -1.95883200 1.30541500 0.79344500
H -2.59592400 3.64726500 1.12027400
H -2.78346000 3.88529100 -0.60627600
H -0.39080000 3.52653400 -0.99771100
H -0.63309300 4.78887000 0.19129500
H -0.34501500 2.68482200 1.93836500
H 1.95152400 2.26658000 1.73316700
H 3.19389800 3.72897500 0.20254900
H 1.81783000 3.91327400 -0.86437300
H 2.03576700 1.56446700 -1.62170000
H 3.49420900 2.49002600 -1.90215200
H 3.12797200 0.64079000 0.50043400
H 4.59998100 1.47094900 0.03605200
H 3.16189900 -0.69608900 -1.57475700
H 4.49653200 0.23121100 -2.19711500
H 5.98878400 -0.56527600 -0.38539500
H 5.40810100 -1.84452100 -1.42676300
H 5.46753500 -2.49141300 0.94757500
H 4.33800400 -1.23087900 1.38140500
H 3.59542600 -3.36565200 -0.70099700
H 3.50067100 -3.69164300 1.02841800
H 0.63490700 -0.17138100 2.12315700
H -0.03551200 -1.82185800 2.24064900
H -1.25816300 -0.68770100 0.42368500
H -0.37348000 -2.08886800 -1.54994300
H -0.69615500 -3.01565900 -0.05202300

conf_0 15.7 kJ mol^{-1}

O -1.24916000 -1.24078700 -0.74088300
C 0.03281800 -0.60241400 -0.43846400
C 0.67111500 -1.30704500 0.73705700
O 0.97193100 -2.64318600 0.43097100
H 1.80233600 -2.69583500 -0.05382200
C -0.33874300 -1.34593600 1.85345300
O -1.56533400 -1.97453700 1.36926400
C -1.90757700 -1.88479600 0.14160800
C -3.12264100 -2.60072700 -0.29924400
C -4.10293300 -1.75008600 -1.12413800
C -4.98848100 -0.84182500 -0.27500600
C -4.25423000 0.18684800 0.57875700
C -3.49616200 1.25383300 -0.20192700
C -2.66403500 2.15187500 0.70459000
C -1.97146100 3.31440900 -0.01027900
C -1.00853600 2.89770400 -1.07907300
C 0.30846100 3.12370700 -1.09129000
C 1.11775000 3.82866000 -0.04304700
C 2.57111500 3.36530700 0.00850000
C 2.73652500 1.88768000 0.33643800
C 4.19527000 1.46944700 0.47600200
C 4.40749800 -0.02032700 0.72605800
C 4.10017700 -0.90882000 -0.47320800
C 4.32511300 -2.39153600 -0.20355600
C 3.97842800 -3.28023300 -1.39106800
H 2.93509500 -3.15985900 -1.71562900
H 4.59858100 -3.03345200 -2.25734300
H 4.13035800 -4.33784600 -1.16345000
H 3.75508300 -2.69792100 0.68641000
H 5.37389300 -2.54909400 0.07056200
H 3.06500400 -0.74920700 -0.81296200
H 4.72410100 -0.59801100 -1.32050000
H 3.80560000 -0.33804700 1.59138600
H 5.44885200 -0.18995600 1.02174600
H 4.74265800 1.76346500 -0.42874800
H 4.64376900 2.03987000 1.29705700
H 2.20420700 1.66538800 1.27464800
H 2.24967900 1.29663500 -0.45118800
H 3.05169100 3.57251500 -0.95606900
H 3.11096800 3.96322600 0.75090800
H 0.65856900 3.71063200 0.94502700
H 1.10280000 4.90614200 -0.25373900
H 0.86358400 2.81233200 -1.97744400
H -1.44197100 2.41301600 -1.95330900
H -2.74372000 3.94600100 -0.46891500
H -1.47032800 3.94267100 0.73017000
H -1.90391800 1.54671800 1.22319800
H -3.30722800 2.55726300 1.49434700
H -2.85236700 0.79228200 -0.95952000
H -4.21498600 1.86684900 -0.75972300
H -3.57172700 -0.32228100 1.27579600
H -4.98367700 0.68032000 1.23032500
H -5.68390000 -0.32507700 -0.94568600
H -5.60864500 -1.46844400 0.37636700
H -4.73611000 -2.44642900 -1.67917100
H -3.54661500 -1.18231000 -1.87548300
H -3.59746700 -3.03479900 0.58271800
H -2.74776200 -3.42805100 -0.91661500
H -0.01803200 -1.98103100 2.67568100
H -0.61337100 -0.35290900 2.21330700
H 1.54367000 -0.73223200 1.06749500

H	0.60566800	-0.71728400	-1.35730100
H	-0.17065700	0.45848700	-0.26833000

conf_102 21.2 kJ mol^{-1}

O	2.58894200	1.90348700	-1.19678600
C	3.25266500	0.69778100	-0.70823300
C	2.94416400	0.51598700	0.76298300
O	1.57520500	0.32285400	0.97993100
H	1.31514200	-0.56363800	0.69727900
C	3.31639200	1.79195300	1.47149000
O	2.66352600	2.92398700	0.81475400
C	2.31951000	2.87659300	-0.41376100
C	1.54932000	4.01217700	-0.95305200
C	0.14727500	4.06681600	-0.31250400
C	-0.66847400	2.80197800	-0.53728500
C	-2.08268300	2.92032900	0.01304000
C	-2.90269900	1.65116200	-0.16789700
C	-4.33880800	1.80319700	0.31296000
C	-5.15776100	0.51856300	0.17403200
C	-4.71666600	-0.56129200	1.11139500
C	-4.26044700	-1.77386700	0.79532800
C	-4.06340900	-2.34592400	-0.57593000
C	-2.83559800	-3.24973700	-0.66573200
C	-1.52016700	-2.53589500	-0.38268000
C	-0.32380700	-3.47419200	-0.45319100
C	1.00295600	-2.81441200	-0.10422700
C	2.19854600	-3.75370800	-0.18128300
C	3.52512900	-3.10128000	0.18327200
C	4.70328100	-4.06242700	0.12725300
H	4.81812100	-4.48375800	-0.87565600
H	5.64281900	-3.57017300	0.39203200
H	4.55874000	-4.89581500	0.82053900
H	3.44933000	-2.67607400	1.19468900
H	3.71305200	-2.26144500	-0.50310100
H	2.02417100	-4.60660400	0.48564500
H	2.26578800	-4.17035100	-1.19373900
H	0.93095900	-2.41643000	0.92224100
H	1.16224700	-1.96468800	-0.79003400
H	-0.48536800	-4.31846200	0.22853100
H	-0.25449500	-3.90678900	-1.45912600
H	-1.39133600	-1.71266300	-1.10005400
H	-1.56813300	-2.06993800	0.61077600
H	-2.95061400	-4.07902300	0.04455200
H	-2.79474600	-3.70615400	-1.66159800
H	-3.99053400	-1.54795900	-1.32272400
H	-4.94769100	-2.93735600	-0.84790500
H	-4.02279000	-2.45097600	1.61673600
H	-4.80742900	-0.31710400	2.16998600
H	-6.20882200	0.74957700	0.38720100
H	-5.12775500	0.17485200	-0.86487800
H	-4.82761600	2.60507400	-0.25291200
H	-4.34126000	2.12283400	1.36339800
H	-2.90806500	1.36866000	-1.22970500
H	-2.42515400	0.82059600	0.36755100
H	-2.59231600	3.75864200	-0.47949800
H	-2.03835000	3.17510300	1.07987800
H	-0.71290600	2.57979100	-1.61185900
H	-0.17098700	1.94543600	-0.06165400
H	-0.36083800	4.93181300	-0.74892400
H	0.25169200	4.26986100	0.75800200
H	1.48580300	3.89335900	-2.03567300
H	2.10282700	4.92797500	-0.72235900
H	2.93717800	1.81750900	2.49054700
H	4.38814200	1.99356300	1.45475100
H	3.56243500	-0.30039300	1.15612900
H	2.84599300	-0.09994200	-1.32690200
H	4.31906000	0.81565300	-0.91120800

conf_174 28.3 kJ mol^{-1}

O	4.08362600	-0.21540500	0.95914300
C	3.87877100	1.20806300	1.22153300
C	2.72209700	1.71706700	0.40118900
O	1.57264500	1.10247600	0.90972200
H	0.78131200	1.53887300	0.57458000
C	2.97601900	1.34539200	-1.04452500
O	3.30821500	-0.07184000	-1.15423900
C	3.78697300	-0.73154100	-0.17155800
C	4.10695200	-2.15920400	-0.37699200
C	3.13950900	-2.93964700	-1.27083000
C	1.84139500	-3.32604100	-0.57254300
C	0.98744500	-2.15175300	-0.11781600
C	-0.33665800	-2.58451900	0.49611600
C	-1.18140200	-1.41416500	0.98057800
C	-2.51578000	-1.83108300	1.60944200
C	-3.42017200	-2.54433300	0.65210100
C	-4.56702900	-2.09583900	0.13820600
C	-5.24649700	-0.78798600	0.39169500
C	-5.28800200	0.10299900	-0.85639200
C	-3.92329500	0.60245000	-1.31998700
C	-3.24936900	1.54807000	-0.33398000
C	-1.96671100	2.16775400	-0.87033600
C	-1.27829600	3.07647000	0.13895300
C	-0.07724300	3.82881300	-0.42156400
C	0.64388600	4.67556300	0.61745500
H	1.50866400	5.19585800	0.19465200
H	-0.02467900	5.43918500	1.02399500
H	0.98278000	4.07306900	1.46905000
H	-0.41001500	4.46571100	-1.24848700
H	0.62168300	3.11633100	-0.89031900
H	-2.00088500	3.80826700	0.52016100
H	-0.98522900	2.49006400	1.02536100
H	-2.19225100	2.74057900	-1.77891500
H	-1.27842700	1.36797200	-1.18514800
H	-3.94790200	2.35298900	-0.06960700
H	-3.02840400	1.02166600	0.60256000
H	-3.26711100	-0.25651500	-1.51340200
H	-4.04473900	1.11911900	-2.27991500
H	-5.76230300	-0.45431300	-1.67250800
H	-5.93747100	0.96360500	-0.65600100
H	-4.78056700	-0.25098600	1.22260100
H	-6.27864600	-0.98960900	0.70220700
H	-5.08683200	-2.75002000	-0.56186900
H	-3.09721700	-3.53829400	0.34700400
H	-2.30905200	-2.48633300	2.46608800
H	-3.00975100	-0.94466800	2.01619900
H	-1.38378400	-0.74177500	0.13678000

H	-0.60582300	-0.83651700	1.71579200
H	-0.89576900	-3.16351800	-0.24745100
H	-0.14345800	-3.26593100	1.33506200
H	0.78923400	-1.48922700	-0.97193200
H	1.52396100	-1.54340800	0.62528500
H	1.26017600	-3.94749100	-1.26239200
H	2.06979100	-3.96659800	0.28893800
H	3.66524600	-3.84239900	-1.59199500
H	2.93459000	-2.36295100	-2.17833500
H	4.22546100	-2.61499400	0.60923800
H	5.10764700	-2.14385100	-0.83542600
H	2.09245100	1.46169300	-1.66993000
H	3.81182300	1.89526900	-1.48191000
H	2.69241400	2.81114000	0.48174200
H	3.67192000	1.25811700	2.28784400
H	4.81969900	1.70885900	0.98891300

Dehydroxyl-DAG(18:1/18:1) + H − 282]$^+$

Figure S13

DIOXOLANE

conf_0 0.0 kJ mol^{-1}

O	0.88271500	-3.03617300	0.84630900
C	0.91000800	-1.98488800	0.10940900
O	0.39131900	-0.93696700	0.61284000
C	-0.15353000	-1.23669400	1.95466300
C	0.25312000	-2.70632800	2.12205700
H	-0.58387900	-3.39030400	2.25051600
H	1.00405600	-2.86754200	2.89565700
C	0.42479500	-0.27191400	2.94843100
H	1.51400700	-0.35000100	2.99427800
H	0.01599100	-0.49410200	3.93810000
H	0.14570000	0.75011000	2.68815200
H	-1.22979100	-1.10285200	1.83950700
C	1.56156100	-1.96942600	-1.20440400
C	0.83254300	-1.13167700	-2.26270600
C	-0.38599400	-1.83558100	-2.85226300
C	-1.51792500	-2.12961400	-1.87281000
C	-2.23088200	-0.90021300	-1.32492200
C	-3.39298800	-1.25147400	-0.40517800
C	-4.05287400	-0.02247900	0.22734400
C	-3.21532100	0.62273400	1.28930900
C	-2.58415100	1.79786400	1.22299400
C	-2.57316100	2.76116400	0.07561300
C	-1.24481400	3.49738800	-0.08696100
C	-0.07826300	2.59650800	-0.46810200
C	1.22881500	3.36416200	-0.63002000
C	2.43386500	2.50193200	-0.99356700
C	2.87158000	1.55309700	0.11475000
C	4.08646200	0.70763800	-0.24287500
C	4.46565200	-0.28309000	0.84854000
H	4.70652300	0.23460200	1.78191000
H	5.33432600	-0.88584400	0.57199800
H	3.64227500	-0.97602900	1.07135000
H	3.89911700	0.17884100	-1.18882900
H	4.93953200	1.36381800	-0.44831900
H	2.03939800	0.89145300	0.38280900
H	3.09360800	2.13474900	1.01969300
H	2.21065900	1.92642100	-1.90320300
H	3.27608600	3.15379200	-1.25248200
H	1.44431100	3.91037900	0.29796800
H	1.09049300	4.12972000	-1.40212800
H	0.03146100	1.81041800	0.28945800
H	-0.31577000	2.08079700	-1.41003400
H	-1.00665500	4.02170300	0.84793600
H	-1.36148000	4.27714900	-0.84765000
H	-2.83242400	2.25420600	-0.85953000
H	-3.36253200	3.50511400	0.24748100
H	-2.06134100	2.14188100	2.11718900
H	-3.17000300	0.07961300	2.23562500
H	-5.00427600	-0.32517100	0.67880300
H	-4.30482800	0.69522300	-0.55889300
H	-4.14339600	-1.81561500	-0.97008600
H	-3.05027700	-1.93123600	0.39047800
H	-2.61256200	-0.31115500	-2.16857900
H	-1.53290800	-0.24167600	-0.79552300
H	-2.25122900	-2.76760500	-2.37878700
H	-1.15300300	-2.75564800	-1.04055400
H	-0.05825400	-2.77219400	-3.31842700
H	-0.77405500	-1.21471800	-3.66702600
H	0.56883900	-0.15953900	-1.83638100
H	1.55458100	-0.93166400	-3.05813400
H	1.72238800	-3.00369200	-1.51750700
H	2.55205000	-1.53292600	-1.00583500

conf_1 3.4 kJ mol^{-1}

O	-0.23549100	-0.95689300	-0.63874700
C	-0.49666100	-2.04221700	-0.01410500
O	-0.09619800	-3.11995200	-0.57241400
C	0.60122300	-2.79169900	-1.84035500
C	0.51243100	-1.25991300	-1.85148200
H	-0.05570200	-0.84994500	-2.68482100
H	1.47168100	-0.75042800	-1.75454800
C	1.98839200	-3.36576700	-1.80660200
H	2.56976300	-2.94414900	-0.98309300
H	2.49424800	-3.12268000	-2.74525900
H	1.95811800	-4.45204500	-1.71045900
H	-0.01387000	-3.24850700	-2.61644600
C	-1.27082400	-2.03641400	1.23144500
C	-0.90623100	-0.90761800	2.20543000
C	0.35786100	-1.19290600	3.01003300
C	1.64481700	-1.31000900	2.19978800
C	2.10247600	-0.02002900	1.53151400
C	3.44146200	-0.16523000	0.82044200
C	3.85824000	1.09481100	0.05664500
C	3.03127700	1.33952000	-1.16841000
C	2.13378000	2.30944900	-1.36234200
C	1.76178300	3.40383600	-0.40957000
C	0.28476500	3.78898600	-0.46092400
C	-0.66276900	2.69430100	0.01020400
C	-2.12007600	3.14224700	0.00699700
C	-3.11569800	2.08196200	0.46728600
C	-3.27296300	0.91532600	-0.49959400
C	-4.27348600	-0.13401000	-0.03423100

C	-4.40209900	-1.30657500	-0.99611700
H	-4.74940400	-0.97161800	-1.97787700
H	-5.10896500	-2.05767600	-0.63443800
H	-3.43835300	-1.80946200	-1.15662300
H	-3.99085900	-0.49016200	0.96726200
H	-5.25549300	0.33401600	0.09745600
H	-2.29930900	0.43752100	-0.66592000
H	-3.58681600	1.30050500	-1.47890900
H	-2.81629700	1.70426100	1.45528500
H	-4.09592900	2.55037700	0.61262600
H	-2.39316100	3.47967800	-1.00164000
H	-2.21335100	4.02327100	0.65244400
H	-0.53282500	1.80735600	-0.62279400
H	-0.38036600	2.38910300	1.02846700
H	0.02152800	4.07956600	-1.48638900
H	0.13281800	4.68333900	0.15358500
H	2.03753000	3.13676900	0.61576900
H	2.35843600	4.29036200	-0.66334400
H	1.64997500	2.36394500	-2.33904800
H	3.23289500	0.66363600	-2.00243600
H	4.90584900	0.99348700	-0.24832700
H	3.82010000	1.95490600	0.73138000
H	4.21535700	-0.41928900	1.55326600
H	3.40406000	-1.01453900	0.12151800
H	2.19082600	0.76089700	2.29756100
H	1.35111100	0.34557700	0.82215500
H	2.43847500	-1.66340900	2.86770800
H	1.55220600	-2.11187300	1.44787400
H	0.20678000	-2.11378200	3.58548700
H	0.47577000	-0.39311600	3.74924800
H	-0.83124500	0.03561800	1.65691300
H	-1.75190400	-0.79728400	2.88859500
H	-1.19487000	-3.02774000	1.68389100
H	-2.31149700	-1.91002000	0.89685700

conf_67 12.1 kJ mol^{-1}

O	-0.51243000	-3.23591100	-0.67200100
C	-1.14170900	-2.18336500	-1.04487500
O	-0.44996300	-1.30594800	-1.65849100
C	0.97325300	-1.72268900	-1.68763100
C	0.88145700	-3.13264700	-1.09600700
H	1.49485600	-3.27880700	-0.20908900
H	1.05004900	-3.92999600	-1.82049300
C	1.49650900	-1.62783200	-3.09018200
H	0.94815000	-2.28452900	-3.77030600
H	2.54883400	-1.92579800	-3.09442600
H	1.43749500	-0.60044400	-3.45218800
H	1.47315200	-1.02720700	-1.00900700
C	-2.57460100	-1.98596900	-0.79771300
C	-2.85605000	-0.74330000	0.08199100
C	-2.84989000	-1.02782200	1.57956300
C	-1.57165400	-1.63042500	2.15322000
C	-0.31335100	-0.78487900	1.99146200
C	0.87240800	-1.37019500	2.74866300
C	2.16886100	-0.57477900	2.60036200
C	2.79344300	-0.69259500	1.24544100
C	3.30664200	0.28236800	0.48932000
C	3.42627100	1.73567800	0.82660700
C	3.16595800	2.70680900	-0.33020200
C	1.69876300	3.04023900	-0.57950600
C	0.84792100	1.87683700	-1.07324000
C	-0.58331700	2.26611700	-1.43015900
C	-1.41586200	2.73222000	-0.24189300
C	-2.87508500	2.99155000	-0.59191100
C	-3.72024500	3.35947100	0.61802900
H	-3.71588300	2.55689000	1.36419500
H	-4.76029400	3.54953000	0.34119800
H	-3.33848700	4.26090400	1.10686100
H	-2.92796900	3.79267700	-1.33853600
H	-3.29618000	2.10298300	-1.08156200
H	-0.98575200	3.64584500	0.18392500
H	-1.35883100	1.98087700	0.56033800
H	-0.56686500	3.05438700	-2.19361200
H	-1.08391800	1.40875500	-1.90028700
H	0.82976600	1.09054800	-0.30762700
H	1.33608000	1.44308200	-1.95764300
H	1.27065300	3.44521200	0.34527600
H	1.64263200	3.85014100	-1.31744100
H	3.62058000	2.31200900	-1.24897600
H	3.69334400	3.64117000	-0.11503500
H	2.78932000	1.99628500	1.67836600
H	4.45777900	1.88890600	1.17186600
H	3.77842300	-0.00475600	-0.45245300
H	2.89884300	-1.71800900	0.88250100
H	2.88870700	-0.94686900	3.34084900
H	1.98921500	0.47379000	2.85664100
H	0.61087900	-1.43225900	3.81061700
H	1.04239400	-2.40781400	2.42601800
H	-0.50797700	0.23166000	2.35704000
H	-0.04766800	-0.65785800	0.93205200
H	-1.73595800	-1.80090900	3.22290500
H	-1.40206200	-2.63246000	1.73558400
H	-3.68938400	-1.69249100	1.81384500
H	-3.06465300	-0.08229500	2.09060800
H	-2.14720200	0.04688100	-0.17778800
H	-3.84170500	-0.37024700	-0.20467100
H	-2.98696800	-2.89982700	-0.36701700
H	-3.02258600	-1.83384400	-1.78663400

conf_31 12.2 kJ mol^{-1}

O	-0.41370200	-3.21621700	0.82657700
C	0.27070200	-2.28593700	0.26390900
O	0.36533000	-1.19452000	0.91379300
C	-0.50651800	-1.25535600	2.10892900
C	-0.86027500	-2.74300400	2.13508300
H	-1.92662000	-2.94748500	2.20516700
H	-0.30767200	-3.31457400	2.88224200
C	0.23272800	-0.72093000	3.29944200
H	0.51418900	0.32067500	3.13644400
H	1.12986000	-1.30807400	3.50997500
H	-0.42298200	-0.76057800	4.17364300
H	-1.35895300	-0.62958700	1.83547900
C	0.94323100	-2.48202700	-1.02527400
C	0.79185400	-1.29459200	-1.98843300
C	-0.61183900	-1.13135600	-2.55690800
C	-1.68358400	-0.80227600	-1.52559800

C	-3.04308600	-0.53254600	-2.15786500
C	-4.17334300	-0.25886100	-1.17015300
C	-4.05447200	1.05495500	-0.38854800
C	-3.17874300	1.01371300	0.82563700
C	-2.22791300	1.88608500	1.17209900
C	-1.78886400	3.09744300	0.40789900
C	-0.30376600	3.42130200	0.55140000
C	0.62995600	2.39148800	-0.07166000
C	2.09053900	2.82958900	-0.02926200
C	3.07853700	1.82658600	-0.61930000
C	3.31989000	0.60837600	0.26607700
C	4.18006600	-0.47798600	-0.37609500
C	5.60636800	-0.04182300	-0.68340000
H	6.10682300	0.31982600	0.22037800
H	5.63323400	0.76304300	-1.42231400
H	6.19766900	-0.87050800	-1.08096800
H	4.21199500	-1.34870300	0.29244400
H	3.70058000	-0.81504000	-1.30642300
H	2.35649300	0.17648300	0.55799100
H	3.79610600	0.93749000	1.19956100
H	2.72082200	1.50090600	-1.60713900
H	4.03136600	2.33324900	-0.80123600
H	2.37594200	3.05003000	1.00830200
H	2.17731400	3.77915500	-0.56963100
H	0.50183700	1.42616200	0.43518700
H	0.33599400	2.22666300	-1.11838500
H	-0.05780400	3.54092900	1.61512600
H	-0.11348600	4.39671900	0.09000800
H	-2.04115800	2.99567300	-0.65318000
H	-2.36774700	3.95716300	0.77120900
H	-1.74854800	1.75860100	2.14516200
H	-3.42748600	0.21973000	1.53412400
H	-5.05851500	1.33451700	-0.04334400
H	-3.74356800	1.85640400	-1.06594900
H	-5.11170500	-0.23760200	-1.73263100
H	-4.26749200	-1.09952000	-0.46831000
H	-3.31847100	-1.39115400	-2.78224100
H	-2.95209700	0.32057100	-2.84237200
H	-1.81265900	-1.64515000	-0.82624800
H	-1.37031000	0.05946000	-0.92671700
H	-0.89484600	-2.03568200	-3.11052900
H	-0.57727100	-0.32528900	-3.29809100
H	1.10874700	-0.37977700	-1.47904100
H	1.50425000	-1.46233300	-2.80008200
H	0.58772700	-3.42201300	-1.45312600
H	2.00663000	-2.61152400	-0.77530500

DIOXANE

conf_0 11.7 kJ mol^{-1}

O	0.62862800	-0.88292700	-0.55707000
C	0.02314900	-0.97693200	-1.88699400
C	-0.31416500	-2.41269000	-2.18614400
C	0.90043100	-3.26362900	-1.92227700
O	1.40771200	-3.00056100	-0.57356600
C	1.23585500	-1.86161500	-0.01414600
C	1.84120200	-1.64654200	1.31447700
C	1.02716200	-0.77345500	2.27336200
C	-0.11726800	-1.52399500	2.94819500
C	-1.23087300	-2.00936400	2.02516100
C	-2.06105000	-0.90155300	1.38958800
C	-3.24028000	-1.43149200	0.58477500
C	-4.04229100	-0.32871600	-0.11185300
C	-3.32399200	0.28999600	-1.27145200
C	-2.83460200	1.53041300	-1.35519000
C	-2.89403200	2.60322000	-0.31176700
C	-1.64094400	3.47525400	-0.26260900
C	-0.38150800	2.73291400	0.16256900
C	0.83592000	3.64663900	0.24750900
C	2.12758700	2.95098800	0.66562500
C	2.66961200	1.97549200	-0.37109800
C	3.95669100	1.28197200	0.05410000
C	4.44207900	0.25319200	-0.95695800
H	4.64824400	0.71996500	-1.92471700
H	5.35808900	-0.24477400	-0.62880500
H	3.68737100	-0.52585400	-1.13370100
H	3.80833600	0.80999400	1.03585200
H	4.74008600	2.03158300	0.21239600
H	1.91103600	1.21488200	-0.59098000
H	2.84311700	2.51069700	-1.31421800
H	1.96626500	2.42328400	1.61635300
H	2.89278700	3.70847300	0.87074200
H	0.98801500	4.13795700	-0.72266300
H	0.61705700	4.45109700	0.95915300
H	-0.55243600	2.26315400	1.14205300
H	-0.19283500	1.91170600	-0.54111800
H	-1.47760600	3.92997900	-1.24844300
H	-1.81706800	4.30786500	0.42747300
H	-3.08312700	2.17276200	0.67688700
H	-3.75541400	3.24734200	-0.53355700
H	-2.38470100	1.83453200	-2.30227300
H	-3.24302800	-0.34683900	-2.15485800
H	-4.98168900	-0.75576000	-0.48103600
H	-4.32273000	0.43148800	0.62296600
H	-3.90449400	-1.99535400	1.24920300
H	-2.88926300	-2.15469400	-0.16645000
H	-2.44231500	-0.24904100	2.18564400
H	-1.44002500	-0.25917500	0.75464700
H	-1.89589000	-2.65904500	2.60541600
H	-0.82253500	-2.67114300	1.24313200
H	0.29911000	-2.37913500	3.49367800
H	-0.55278100	-0.86687000	3.70889600
H	0.66968400	0.11505000	1.74538800
H	1.71944500	-0.41469600	3.03926900
H	2.79894000	-1.15412400	1.08839300
H	2.08154500	-2.62502500	1.73698000
H	0.69640400	-4.33223600	-1.92315400
H	1.72919700	-3.04955800	-2.60036400
H	-0.61349300	-2.51157800	-3.23226700
H	-1.15485200	-2.74131400	-1.56843500
H	-0.84947300	-0.32670500	-1.81819200
H	0.75821900	-0.55157900	-2.57344500

conf_1 13.6 kJ mol^{-1}

O	1.28491900	-3.04927100	-0.40505600
C	0.70862700	-3.37440600	-1.71024600
C	0.76323400	-2.16471800	-2.60622800

C 0.11094100 -1.01002100 -1.89386500
O 0.66609900 -0.88359200 -0.54601100
C 1.20665000 -1.86119600 0.06650000
C 1.83409000 -1.58466500 1.37312900
C 1.00524900 -0.68510300 2.29921800
C -0.13586900 -1.42415200 2.99125000
C -1.21764100 -1.98355400 2.07246800
C -2.06806300 -0.93642200 1.36533700
C -3.17040200 -1.55101500 0.51304600
C -4.02019200 -0.51042800 -0.22218300
C -3.29879900 0.15223400 -1.35553000
C -2.86010700 1.41207400 -1.41334000
C -2.99729100 2.47224500 -0.36400000
C -1.78790800 3.40160000 -0.28020800
C -0.50561400 2.71356700 0.16703400
C 0.67684700 3.67200800 0.25058000
C 1.98948200 3.02757300 0.68534400
C 2.57022400 2.05679000 -0.33473000
C 3.87443200 1.40847100 0.10904600
C 4.40143700 0.38661100 -0.88816300
H 4.60542800 0.85186000 -1.85719400
H 5.32778300 -0.08168700 -0.54560400
H 3.67069400 -0.41428700 -1.06636900
H 3.73057600 0.93995800 1.09306700
H 4.63209100 2.18389000 0.26834300
H 1.83842600 1.26898200 -0.55000100
H 2.73460200 2.58557600 -1.28316700
H 1.84122900 2.50803200 1.64262600
H 2.72711500 3.81367300 0.88358400
H 0.81845200 4.15730000 -0.72425400
H 0.42402900 4.47625800 0.95110300
H -0.66886000 2.25031700 1.15089900
H -0.28176100 1.89015200 -0.52371900
H -1.62417400 3.86937300 -1.25991600
H -2.01663700 4.22094000 0.41027200
H -3.18922200 2.02546000 0.61689600
H -3.88259400 3.07694400 -0.60201800
H -2.39197000 1.74224900 -2.34234400
H -3.16531400 -0.46941800 -2.24337400
H -4.91223500 -1.00365400 -0.62435400
H -4.38152600 0.23089900 0.49653800
H -3.81963100 -2.16191100 1.15036500
H -2.73323900 -2.24808100 -0.21921600
H -2.52623100 -0.28794300 2.12299000
H -1.44957200 -0.27544400 0.74700000
H -1.87523000 -2.62573800 2.66918400
H -0.77474500 -2.67103400 1.33242900
H 0.28754000 -2.24044600 3.58821100
H -0.60033000 -0.73811400 3.70810900
H 0.64298000 0.17996200 1.73682600
H 1.69023100 -0.29286000 3.05512500
H 2.77789700 -1.08016600 1.12153000
H 2.08734400 -2.54038800 1.83678300
H 0.31152400 -0.04181500 -2.34912600
H -0.96589800 -1.11954900 -1.75483000
H 0.23042600 -2.37131100 -3.53748400
H 1.79994100 -1.92691400 -2.86278000
H 1.30771200 -4.21082100 -2.06345000
H -0.31096700 -3.71229400 -1.51089700

conf_70 21.7 kJ mol^{-1}

O -0.35130900 -1.63570200 -2.28025200
C 0.52567800 -2.80601500 -2.27806800
C 1.62909900 -2.60631400 -1.27334600
C 2.30183600 -1.28960500 -1.55634200
O 1.29294500 -0.23022400 -1.63513300
C 0.07920200 -0.47922800 -1.93803000
C -0.90927900 0.60924500 -1.86752000
C -0.36128000 1.99332400 -1.54661900
C -1.47766400 3.02619500 -1.35006300
C -1.96437000 3.17156800 0.09314800
C -2.51662200 1.91135800 0.75333800
C -3.81348000 1.38311600 0.15060700
C -4.19471800 0.00146300 0.69612300
C -3.35496300 -1.09638700 0.11923800
C -2.47161800 -1.87925000 0.74572400
C -2.09923900 -1.87817100 2.19524400
C -0.58816400 -1.93086100 2.42615800
C 0.13754300 -0.68217800 1.94914300
C 1.64494800 -0.71689900 2.14973000
C 2.34137800 0.53989000 1.64532800
C 3.86138000 0.47356500 1.72400100
C 4.56869100 1.68672700 1.12643000
C 4.41831900 1.80675300 -0.38511300
H 3.37275300 1.92468200 -0.68886200
H 4.96346600 2.67205900 -0.77005800
H 4.82325600 0.92086300 -0.88945000
H 5.63376400 1.63432700 1.37418000
H 4.19145900 2.59728800 1.60743700
H 4.15702700 0.35825900 2.77295700
H 4.21925600 -0.43663800 1.21758400
H 1.98929500 1.40489200 2.22181800
H 2.02713900 0.73317700 0.61157000
H 1.87018200 -0.86133400 3.21323600
H 2.06671100 -1.59993900 1.64564300
H -0.08608500 -0.51907400 0.88413600
H -0.27575900 0.19325200 2.46580100
H -0.39213200 -2.07733300 3.49376600
H -0.17564000 -2.81782000 1.92289200
H -2.55574200 -2.75652300 2.66940900
H -2.51648900 -1.00373300 2.70348100
H -1.98514800 -2.65627200 0.15115700
H -3.52073400 -1.28432200 -0.94309300
H -4.13847400 0.01116700 1.78860700
H -5.24104300 -0.20556700 0.44390000
H -3.74417700 1.32746100 -0.94418500
H -4.62019100 2.09409200 0.35825200
H -1.75468400 1.12213900 0.75069000
H -2.69356800 2.12488600 1.81406500
H -2.72838400 3.95705300 0.12127600
H -1.12869800 3.54482900 0.69767100
H -1.10422200 4.00115900 -1.67546100
H -2.31554400 2.79751300 -2.01990000
H 0.30073300 2.30674100 -2.35941500
H 0.25726900 1.94468100 -0.64394900
H -1.62917200 0.26005600 -1.11088900

H -1.47031700 0.59278700 -2.81001000
H 2.96786700 -0.95504100 -0.76347600
H 2.82957600 -1.26960400 -2.51180400
H 2.35653700 -3.41757900 -1.35285400
H 1.22556300 -2.61630200 -0.25646700
H -0.13901800 -3.63381400 -2.03958900
H 0.89021800 -2.91484400 -3.30164900

conf_74 22.0 kJ mol^{-1}

O -1.78654900 -1.11865400 -1.49871600
C -0.52488300 -0.40459700 -1.73992300
C 0.63734900 -1.25008700 -1.29433000
C 0.36585800 -1.74115300 0.10174100
O -0.93743800 -2.40916900 0.14647300
C -1.89190700 -2.03407700 -0.61769200
C -3.19164500 -2.72920700 -0.50806100
C -4.39812100 -1.78778100 -0.33947500
C -4.56664900 -1.25020600 1.07831500
C -3.43429100 -0.37137500 1.60078200
C -3.24845400 0.95094300 0.86692900
C -2.09185400 1.76823200 1.42837500
C -1.91168000 3.14258700 0.77538800
C -1.61615200 3.07228500 -0.69158100
C -0.42289900 3.23018700 -1.27268700
C 0.88658700 3.49249300 -0.59741900
C 1.75862800 2.23717400 -0.52826800
C 3.15282600 2.48847400 0.03057400
C 4.02309900 1.23671100 0.09669400
C 3.57580500 0.22081100 1.14314600
C 4.45919000 -1.01967200 1.23489700
C 4.42496400 -1.91807700 0.00361100
C 5.27528400 -3.16994700 0.16125600
H 6.32355700 -2.90870500 0.33309900
H 4.94358500 -3.77023800 1.01438000
H 5.23489200 -3.80183400 -0.72985100
H 4.76078100 -1.36055200 -0.87801600
H 3.38323800 -2.20974000 -0.20108000
H 5.49513400 -0.71003700 1.42250300
H 4.16160400 -1.60949000 2.11089000
H 2.54050600 -0.09332000 0.94543900
H 3.54961900 0.71718100 2.12082100
H 4.05602900 0.77267600 -0.89787000
H 5.05520100 1.52891300 0.32214500
H 3.64800200 3.24021300 -0.59441500
H 3.06985200 2.93148000 1.03144600
H 1.23648600 1.49282700 0.08856300
H 1.85229900 1.80702600 -1.53667900
H 0.73969200 3.88177900 0.41428200
H 1.42721200 4.26754000 -1.15328400
H -0.37946900 3.15277600 -2.35961000
H -2.46768500 2.88295300 -1.34405500
H -2.82849600 3.72484700 0.92684800
H -1.11683800 3.67849700 1.30040300
H -1.15396300 1.20185400 1.32389700
H -2.24072600 1.90507800 2.50582800
H -3.09581900 0.78127200 -0.20588800
H -4.17452600 1.53451700 0.94439400
H -2.48769200 -0.93302300 1.60642300
H -3.62647800 -0.15998900 2.65858400
H -5.50288800 -0.68204600 1.11203800
H -4.70592500 -2.09769500 1.75962300
H -5.28489500 -2.36718500 -0.60779500
H -4.33115600 -0.97810000 -1.07186100
H -3.12168900 -3.45144000 0.30748100
H -3.30048600 -3.28883200 -1.44602100
H 1.06610800 -2.49822000 0.44700800
H 0.31022500 -0.93297100 0.83475500
H 1.54794200 -0.64652100 -1.29594400
H 0.79531300 -2.09424500 -1.97203400
H -0.53902800 -0.20979200 -2.81037600
H -0.61396100 0.54282200 -1.20176800
